# A Facile Oxidative Opening of the C-Ring in Luotonin A and Derivatives

**DOI:** 10.3390/molecules22091540

**Published:** 2017-09-12

**Authors:** Amra Ibric, Kathrin Dutter, Brigitte Marian, Norbert Haider

**Affiliations:** 1 Department of Pharmaceutical Chemistry, University of Vienna, Althanstraße 14, A-1090 Vienna, Austria; amra.ibric@univie.ac.at (A.I.); kathrin_dutter@gmx.at (K.D.); 2 Institute of Cancer Research, Medical University of Vienna, Borschkegasse 8a, A-1090 Vienna, Austria; brigitte.marian@meduniwien.ac.at

**Keywords:** Luotonin A, quinazoline, quinoline, ring opening, oxidation, anticancer activity

## Abstract

An oxidative ring opening reaction of the central ring C in the alkaloid Luotonin A and two of its derivatives was found to occur upon heating with an excess amine and potassium carbonate in dimethylsulfoxide (DMSO) solution in the presence of air oxygen. The structure of the novel amide-type products was elucidated and a possible mechanism for this reaction is proposed. Four of the new compounds show moderate in vitro anticancer activity towards human colon adenocarcinoma cells.

## 1. Introduction

**Figure 1:**
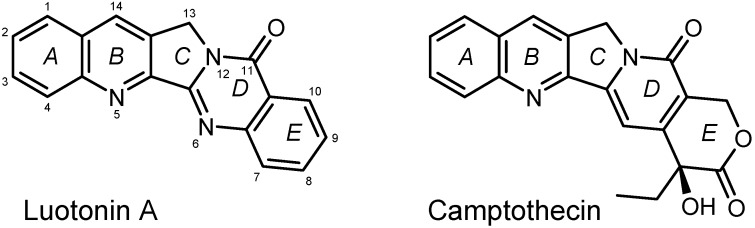
Structures of Luotonin A and Camptothecin (CPT).

In search of new anticancer agents, structural modifications of the alkaloid Luotonin A (first isolated in 1997 [1]) have found considerable interest during the past two decades [2,3,4,5,6,7,8,9,10,11,12]. Like its more prominent (and more potent) naturally occurring relative, Camptothecin (CPT) [13,14,15], Luotonin A can act as a topoisomerase 1 poison by stabilizing the DNA/Topo1 complex [2,16]. Due to its aromatic E-ring (see Figure 1), Luotonin A is principally not associated with bladder toxicity which is a specific adverse effect of CPT-derived drugs like Topotecan or Irinotecan with their labile lactone structure as ring E [14]. On the other hand, the antineoplastic activity of the Luotonin A core structure is markedly lower than that of CPT [16] and thus has stimulated efforts to improve topoisomerase 1 toxicity by various modifications of the lead structure.

Several different strategies have been described for the total synthesis of Luotonin A and related compounds, as summarized in a review article [17]. Among the approaches aiming at variation of the substitution pattern of ring A, there have been some recent investigations of our group in which we made use of two orthogonal cycloaddition routes [18,19,20]: the first route is based on a pathway that was previously explored by Zhou et al. [21] for the synthesis of the parent compound and we found it to be well suited for the introduction of substituents into positions 2 or 4, whereas the second route offers a convenient access to 1- or 3-substituted congeners. Although a number of new Luotonin A derivatives could be made available via these two routes directly, additional modifications by replacement of existing substituents were envisaged in order to further extend our compound library. In particular, nucleophilic substitution of a fluoro functionality with amines appeared quite promising in view of the reported facile transformation of 8-fluoroquinolines into the corresponding 8-(di)alkylaminoquinolines via nucleophilic substitution [22,23]. Herein, we report on the synthesis of a hitherto unknown 4-fluoro derivative of Luotonin A (containing the 8-fluoroquinoline motif) and the observation of an unprecedented oxidative opening of ring C on attempted nucleophilic substitution reactions with primary or secondary amines.

## 2. Results and Discussion

**Scheme 1:**
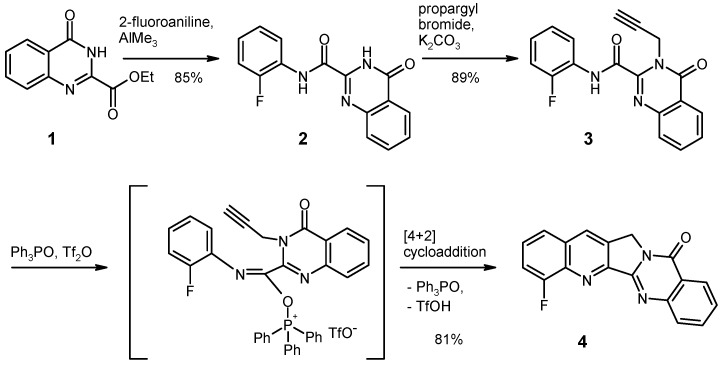
Synthesis of the 4-fluoro derivative (**4**) of Luotonin A.

Among the four possible sites at ring A, position 4 (corresponding to position 8 of the quinoline core) appeared most interesting for the envisaged nucleophilic replacement of a fluorine atom with an amino substituent, as there have been several reports describing the preferential attack of nitrogen nucleophiles at this position in various di- and trifluoroquinolines [24,25,26,27]. We therefore commenced our investigations by preparing the hitherto unknown 4-fluoro derivative of Luotonin A via the pathway depicted in Scheme 1, starting from the quinazolinonecarboxylic acid ester **1** [21,28]. Weinreb amidation [29] with 2-fluoroaniline/trimethylaluminium gave the *N*-arylamide **2** in good yield, selective propargylation at the quinazoline N-3 afforded the key intermediate **3**. On treatment of the latter with bis(triphenyl)oxodiphosphonium triflate (Hendrickson’s reagent [30]) in dry dichloromethane at room temperature, an intramolecular [4+2] cycloaddition smoothly afforded the pentacyclic compound **4** in excellent overall yield.

**Scheme 2:**
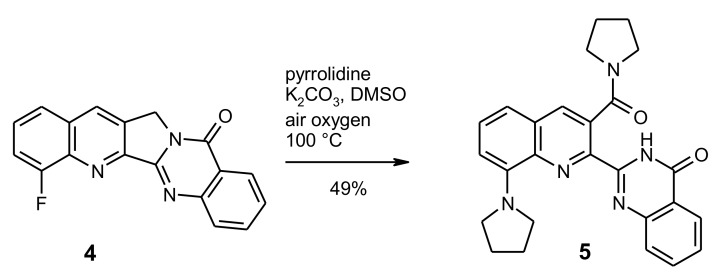
Reaction of the fluoro compound **4** with pyrrolidine: substitution and ring C opening.

In order to achieve the desired nucleophilic fluorine substitution, we chose the conditions that had been described in the patent literature [22,23] for the reaction of 8-fluoroquinoline derivatives with various amines. Thus, the fluoro compound **4** was heated to 100 °C with an excess of pyrrolidine in dimethylsulfoxide (DMSO) solution in the presence of potassium carbonate. As indicated by TLC, the starting material was completely consumed after 24 h and a single new spot was observed. After evaporation of volatiles and extractive work-up, the crude product was purified by medium-pressure liquid chromatography to afford the new compound in 49% yield as red-coloured crystals. ^1^H-NMR-Spectroscopic characterisation surprisingly revealed the presence of two pyrrolidinyl residues in the molecule and the absence of the methylene unit that is part of ring C in the Luotonin A skeleton. On the other hand, the ^13^C-NMR spectrum showed an additional carbonyl resonance (besides the quinazolinone C=O group at 161.4 ppm) at lower field (168.6 ppm). Mass spectrometry (EI-MS (electron-impact ionization) and ESI-TOF (electrospray ionization, time-of-flight) high-resolution MS) indicated a molecular formula of C_26_H_25_N_5_O_2_. Based on the combined spectroscopic evidence, it can be concluded that (a) the expected nucleophilic substitution of the fluorine atom with pyrrolidine has indeed taken place and (b) the C-ring of the pentacyclic scaffold has undergone an oxidative ring opening, involving another pyrrolidine unit that is now attached to the quinoline core via an amide function (structure **5**, Scheme 2).

**Scheme 3:**
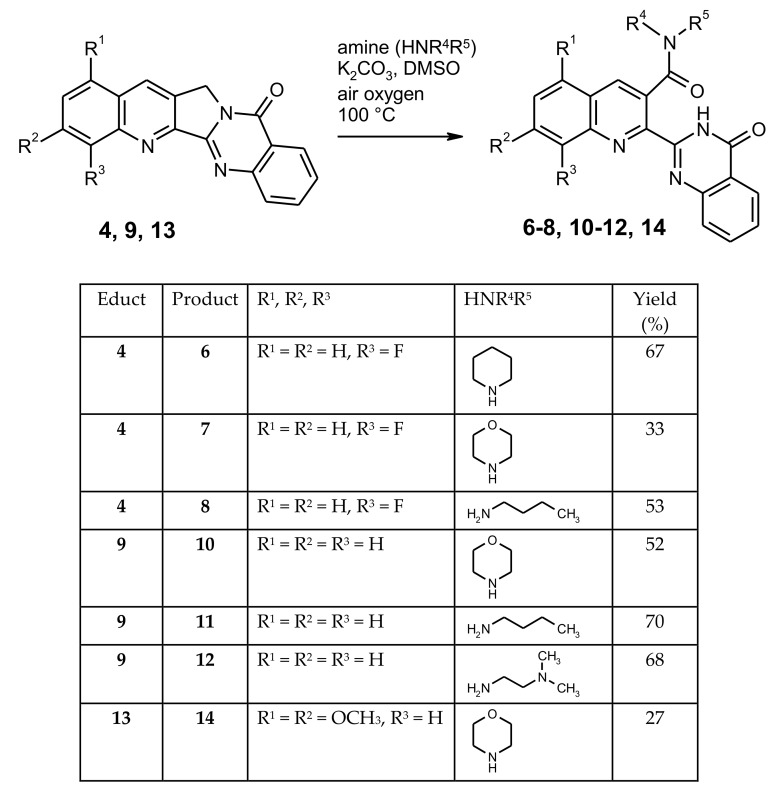
Ring opening reactions of compounds **4**, **9** and **13** with various amines.

To explore the scope of this unexpected reaction, the fluoro compound **4** was reacted with other amines under the same conditions (100 °C, DMSO as solvent, and K_2_CO_3_ as a base). Interestingly, it turned out that only pyrrolidine is reactive enough to effect the fluorine substitution under these conditions, whereas piperidine, morpholine, and *n*-butylamine left the fluoro function untouched, but all led to the formation of ring-opened products with a (tertiary or secondary) carboxamide group (compounds **6**–**8**, Scheme 3). An identical reaction behavior was found with the alkaloid Luotonin A itself (compound **9**), affording the amides **10**–**12** on treatment with morpholine, *n*-butylamine, or *N*,*N*-dimethylethylenediamine, respectively. Also with the electron-rich 1,3-dimethoxy derivative [18] of Luotonin A (compound **13**), an analogous oxidative ring C opening was observed, furnishing the morpholide **14**, albeit in moderate yield. All of the isolated carboxamides share some characteristic NMR properties such as the carbonyl resonance in the ^13^C-NMR spectra at 168–169 ppm (see supplementary material). The position of the secondary or tertiary amide function at C-3 of the quinoline nucleus is evidenced by HMBC (Heteronuclear Multiple Bond Correlation) crosspeaks between this carbonyl signal and the H-4 resonance as well as the NCH_2_ protons in the amide part. The spatial proximity between the latter two kinds of protons is further proven by a corresponding crosspeak in the NOESY (Nuclear Overhauser Effect Spectroscopy) spectrum. Additionally based on HSQC (Heteronuclear Single-Quantum Correlation) and COSY (Correlation Spectroscopy) experiments, all signals in the ^1^H- and ^13^C-NMR spectra could be assigned and are in full agreement with the proposed structures. As with compound **5**, the molecular formulae of all new compounds were confirmed by [M + H]^+^ or [M + Na]^+^ peaks in the high-resolution ESI-TOF mass spectra.

**Scheme 4:**
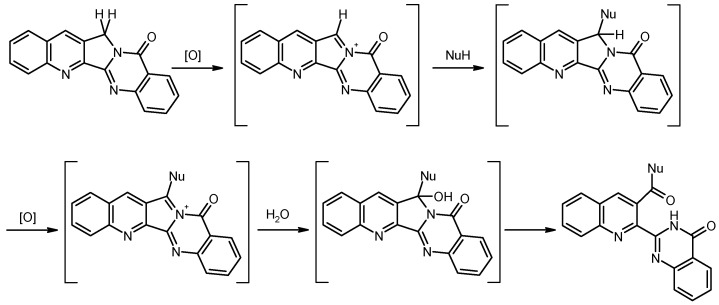
Proposed reaction mechanism for the oxidative/nucleophilic opening of ring C (NuH = primary or secondary amine).

To gain more insight into this remarkable transformation, we varied the solvent and observed essentially the same results when *N*,*N*-dimethylformamide (DMF) was used instead of DMSO. However, with 1-propanol as a protic solvent, a complex reaction mixture was obtained from which no defined product could be isolated. Thus, an aprotic dipolar solvent seems to be favorable in this context. As neither the amine component, nor the inorganic base, nor the solvent have oxidative properties to perform the observed oxidation of the benzylic CH_2_ group of ring C into an amide function, we suspected atmospheric oxygen to play a crucial role in this process. Indeed, when Luotonin A was heated to 100 °C with morpholine/K_2_CO_3_ in DMSO or DMF solution under argon atmosphere, the starting material remained essentially unchanged after several hours [31], whereas a control experiment in the presence of air oxygen showed an almost complete transformation into compound **10** after the same time span. A possible mechanism for this oxidative/nucleophilic ring opening is formulated in Scheme 4: Presumably, the benzylic carbon undergoes a two-step oxidation sequence, involving attack of the amine after the first step and hydrolytic ring cleavage after the second step (promoted by moisture in the solvent or during work-up). To the best of our knowledge, a comparable reaction has not yet been reported for Luotonin A nor for the structurally related CPT.

Although our initial aim, i.e., the synthesis of new A-ring-modified Luotonin A derivatives, was not met, the obtained quinazolinyl-substituted quinoline-3-carboxamides represent interesting and hitherto unknown molecular entities. Therefore, we briefly examined the new compounds for their in vitro growth inhibitory activity towards SW480 human colon adenocarcinoma cells. Indeed, three of the tested compounds (**5**, **6** and **7**) showed moderate activity, slightly exceeding that of the natural lead compound, Luotonin A. Compounds **8**, **11**, **12** and **14** are clearly inferior and compound **10** is approximately equipotent as compared to the reference (Table 1). At present, no valid conclusions can be drawn with respect to structure-activity relationships and the mode of action.

## 3. Experimental

### 3.1. General

Melting points (uncorrected) were determined on a Kofler hot-stage microscope (Leica GmbH, Wetzlar, Germany). ^1^H-NMR and ^13^C-NMR spectra were recorded on a Bruker Avance III 400 spectrometer (Bruker BioSpin GmbH, Rheinstetten, Germany) at 400 MHz and 100 MHz, respectively; chemical shifts (ppm) were referenced to residual amounts of undeuterated solvents. Mass spectra (EI) were obtained on a Shimadzu QP5050A DI 50 instrument (Shimadzu Corp., Kyoto, Japan); high-resolution mass spectra (ESI-TOF) were recorded on a Bruker maXis HD spectrometer (Bruker Daltonics GmbH, Bremen, Germany). Column chromatography was carried out on Merck Kieselgel 60 (Merck, Darmstadt, Germany), 0.063–0.200 mm. Medium-pressure liquid chromato­graphy (MPLC) was performed on a Biotage Isolera One system (Biotage AB, Uppsala, Sweden), using Biotage SNAP cartridges (KP-Sil 10 g). Thin layer chromatography was done on Merck aluminium sheets pre-coated with Kieselgel 60 F_254_ (Merck, Darmstadt, Germany). Microanalyses were performed at the Microanalytical Laboratory, Faculty of Chemistry, University of Vienna. Ethyl 4-oxo-3,4-dihydroquinazoline-2-carboxylate [28] (**1**), Luotonin A (**9**) and its dimethoxy derivative (**13**) were prepared according to literature procedures [18,21]. Amines were purified by distillation prior to use, solvents (analytical grade) were used without further purification.

### 3.2. Procedures

*N-(2-Fluorophenyl)-4-oxo-3,4-dihydroquinazoline-2-carboxamide* (**2**). To a solution of 2-fluoroaniline (0.889 g, 8 mmol) in dry 1,2-dichloroethane (20 mL) under argon was added drop wise a 2 M solution of AlMe_3_ (4.0 mL, 8 mmol) in heptane. The mixture was stirred for 30 min at room temperature, then the ester **1** (1.091 g, 5 mmol) was added in one portion, and the mixture was refluxed for 2 h. After cooling to 0 °C, it was then quenched by slow addition of 2 N HCl (20 mL), followed by water (80 mL). The mixture was exhaustively extracted with CH_2_Cl_2_ and the combined extracts were washed with water and brine, dried over Na_2_SO_4_ and evaporated under reduced pressure. The residue was recrystallized from EtOH to give **2** (1.202 g, 85%) as colorless needles, m.p. 216–217 °C. MS (EI, 70 eV) *m*/*z* = 283 (M^+^, 32%), 236 (18), 146 (91), 119 (100), 118 (18), 91 (15), 90 (31), 83 (12); ^1^H-NMR (DMSO-*d*_6_) δ: 12.62 (br s, 1H, NH), 10.50 (br s, 1H, NH), 8.21 (dd, *J* = 8.0 Hz, 1.2 Hz, 1H, 5-H), 7.97–7.89 (m, 2H, 7-H, phenyl 6′-H), 7.87 (dd, *J* = 8.2 Hz, 0.9 Hz, 1H, 8-H), 7.65 (ddd, *J* = 8.2 Hz, 7.0 Hz, 1.3 Hz, 1H, 6-H), 7.41–7.23 (m, 3H, phenyl 3′-H, 4′-H, 5′-H); ^13^C-NMR (DMSO-*d*_6_) δ: 161.3, 158.0, 154.5 (d, *J*_C–F_ = 246.9 Hz), 146.6, 145.6, 134.8, 128.3, 127.7, 126.9 (d, *J*_C–F_ = 7.7 Hz), 126.2, 124.8, 124.7, 124.6, 122.8, 115.8 (d, *J*_C–F_ = 19.4 Hz). Anal. calcd. for C_15_H_10_FN_3_O_2_·0.2 H_2_O: C, 62.80; H, 3.65; N, 14.65. Found: C, 62.79; H, 3.46; N, 14.66. HRMS (ESI-TOF) *m*/*z* calcd. for C_15_H_11_FN_3_O_2_ ([M + H]^+^): 284.0830. Found: 284.0827.

*N-(2-Fluorophenyl)-4-oxo-3-(prop-2-yn-1-yl)-3,4-dihydroquinazoline-2-carboxamide* (**3**). To a solution of the anilide **2** (0.566 g, 2 mmol) in dry DMF (15 mL) were added K_2_CO_3_ (0.304 g, 2.2 mmol) and propargyl bromide (0.327 g of an 80% solution in toluene, 2.2 mmol). The mixture was stirred at room temperature for 24 h, then it was poured into water (100 mL) and it was extracted with CH_2_Cl_2_ (3 × 100 mL). The combined extracts were washed with water and brine, dried over Na_2_SO_4_ and evaporated under reduced pressure. The residue was recrystallized from EtOH to give **3** (0.527 g, 89%) as colorless crystals, m.p. 153–154 °C. MS (EI, 70 eV) *m*/*z* = 321 (M^+^, 21%), 320 (72), 301 (27), 292 (52), 184 (25), 155 (54), 148 (40), 145 (36), 129 (100), 119 (96), 90 (64), 83 (47), 75 (42), 63 (41); ^1^H-NMR (CDCl_3_) δ: 9.99 (s, 1H, NH), 8.52–8.41 (m, 1H, phenyl 6′-H), 8.37 (ddd, *J* = 8.0 Hz, 1.3 Hz, 0.6 Hz, 1H, 5-H), 7.89–7.79 (m, 2H, 7-H, 8-H), 7.62 (ddd, *J* = 8.2 Hz, 6.5 Hz, 1.9 Hz, 1H, 6-H), 7.24–7.14 (m, 3H, phenyl 3′-H, 4′-H, 5′-H), 5.60 (d, *J* = 2.5 Hz, 2H, CH_2_), 2.27 (t, *J* = 2.5 Hz, 1H, acetylene-H); ^13^C-NMR (CDCl_3_) δ: 161.4, 158.3, 153.1 (d, *J*_C–F_ = 244.0 Hz), 145.0, 144.9, 135.1, 129.3, 128.2, 127.6, 125.7, 125.6 (d, *J*_C–F_ = 7.5 Hz), 124.9 (d, *J*_C–F_ = 3.8 Hz), 121.9, 121.7, 115.3 (d, *J*_C–F_ = 19.1 Hz), 78.9, 72.2, 33.8. Anal. calcd. for C_18_H_12_FN_3_O_2_·0.2 H_2_O: C, 66.54; H, 3.85; N, 12.93. Found: C, 66.53; H, 3.64; N, 12.97. HRMS (ESI-TOF) *m*/*z* calcd. for C_18_H_13_FN_3_O_2_ ([M + H]^+^): 322.0986. Found: 322.0988.

*4-Fluoroquinolino[2′,3′:3,4]pyrrolo[2,1-b]quinazolin-11(13H)-one* (**4**). To a solution of triphenyl­phosphine oxide (0.835 g, 3 mmol) in dry CH_2_Cl_2_ (22 mL) was drop wise added trifluoromethane­sulfonic anhydride (0.25 mL, 1.5 mmol) at 0 °C under argon, and the mixture was stirred at the same temperature for 15 min. Then, compound **3** (0.321 g, 1 mmol) was added in one portion at 0 °C, and the mixture was stirred for 1 h while slowly warming to room temperature. The reaction was quenched by addition of 10% aqueous NaHCO_3_ (15 mL). The phases were separated and the aqueous layer was exhaustively extracted with CH_2_Cl_2_. The combined organic layers were washed with water and brine, dried over Na_2_SO_4_ and evaporated. To remove excess triphenylphosphine oxide, the residue was subjected to column chromatography, eluting first with CH_2_Cl_2_, then with CH_2_Cl_2_/ethyl acetate (19:1). The fraction showing an intense blue fluorescence under UV_366_ was evaporated under reduced pressure and the residue was recrystallized from EtOH to give **4** (0.244 g, 81%) as colorless needles, m.p. > 310 °C (sublimation). MS (EI, 70 eV) *m*/*z* = 304 ([M + 1]^+^, 20%), 303 (M^+^, 100), 302 (36), 275 (13), 274 (10), 262 (8), 152 (12), 77 (7); ^1^H-NMR (CDCl_3_) δ: 8.49 (d, *J* = 1.0 Hz, 1H, 14-H), 8.42 (dd, *J* = 8.0 Hz, 1.1 Hz, 1H, 10-H), 8.13–8.08 (m, 1H, 7-H), 7.86 (ddd, *J* = 8.3 Hz, 7.2 Hz, 1.6 Hz, 1H, 8-H), 7.75 (d, *J* = 8.3 Hz, 1H, 1-H), 7.68–7.49 (m, 3H, 2-H, 3-H, 9-H), 5.37 (d, *J* = 0.8 Hz, 2H, CH_2_); ^13^C-NMR (CDCl_3_) δ: 160.7 (11-C), 158.8 (d, *J*_C–F_ = 261.4 Hz, 4-C), 152.1 (5b-C), 151.7 (5a-C), 149.4 (6a-C), 140.1 (d, *J*_C–F_ = 12.8 Hz, 4a-C), 134.8 (8-C), 131.6 (d, *J*_C–F_ = 2.9 Hz, 14-C), 130.7 (13a-C), 130.3 (14a-C), 129.1 (7-C), 128.7 (d, *J*_C–F_ = 7.8 Hz, 2-C), 127.8 (9-C), 126.6 (10-C), 123.7 (d, *J*_C–F_ = 5.0 Hz, 1-C), 121.5 (10a-C), 114.9 (d, *J*_C–F_ = 18.7 Hz, 3-C), 47.4 (13-C). Anal. calcd. for C_18_H_10_FN_3_O·0.15 H_2_O: C, 70.65; H, 3.39; N, 13.73. Found: C, 70.63; H, 3.30; N, 13.74. HRMS (ESI-TOF) *m*/*z* calcd. for C_18_H_11_FN_3_O ([M + H]^+^): 304.0881. Found: 304.0882.

*2-[8-(Pyrrolidin-1-yl)-3-(pyrrolidin-1-ylcarbonyl)quinolin-2-yl]quinazolin-4(3H)-one* (**5**). To a solution/suspension of the fluoro compound **4** (0.152 g, 0.5 mmol) and K_2_CO_3_ (0.069 g, 0.5 mmol) in DMSO (15 mL) was added pyrrolidine (0.356 g, 5 mmol). The mixture was heated to 100 °C and the color of the solution changed into dark red. Stirring was continued for 24 h at 100 °C, then the mixture was cooled to room temperature and the volatile components were removed by Kugelrohr distillation (10^−1^ mbar, 100 °C). The residue was taken up in CH_2_Cl_2_ (50 mL) and the solution was washed with water and brine, then dried over Na_2_SO_4_ and evaporated under reduced pressure. The residue was subjected to MPLC, eluting with CH_2_Cl_2_/MeOH (94:6) to afford compound **5** (108 mg, 49%) as red crystals, m.p. 210–211 °C (CHCl_3_). MS (EI, 70 eV) *m*/*z* = 439 (M^+^, 9%), 368 (100), 340 (42), 339 (27), 311 (28), 299 (23), 273 (23); ^1^H-NMR (CDCl_3_) δ 10.68 (s, 1H, NH), 8.35 (dd, *J* = 7.9 Hz, 1.1 Hz, 1H, quinazoline 5-H), 8.10 (s, 1H, quinoline 4-H), 7.80–7.73 (m, 1H, quinazoline 7-H), 7.67–7.60 (m, 1H, quinazoline 8-H), 7.55–7.45 (m, 2H, quinazoline 6-H, quinoline 6-H), 7.15 (d, *J* = 7.2 Hz, 1H, quinoline 5-H), 6.84 (d, *J* = 7.8 Hz, 1H, quinoline 7-H), 4.00–3.80 (m, 6H, *N*-arylpyrrolidine 2-H, 2′-H, 5-H, 5′-H, *N*-acylpyrrolidine 2-H, 2′-H), 3.24 (t, *J* = 6.7 Hz, 2H, *N*-acylpyrrolidine 5-H, 5′-H), 2.17–2.10 (m, 4H, *N*-arylpyrrolidine 3-H, 3′-H, 4-H, 4′-H), 2.06 (quint, *J* = 6.9 Hz, 2H, *N*-acylpyrrolidine 3-H, 3′-H), 1.88 (quint, *J* = 6.8 Hz, 2H, *N*-acylpyrrolidine 4-H, 4′-H); ^13^C-NMR (CDCl_3_) δ: 168.6 (amide C=O), 161.4 (quinazoline 4-C), 149.0 (quinazoline 8a-C), 148.7 (quinazoline 2-C), 146.6 (quinoline 8-C), 139.3 quinoline 2-C), 138.4 (quinoline 8a-C), 136.1 (quinoline 4-C), 134.7 (quinazoline 7-C), 130.8 (quinoline 4a-C), 130.5 (quinoline 3-C), 130.3 (quinoline 6-C), 128.3 (quinazoline 8-C), 127.6 (quinazoline 6-C), 127.0 (quinazoline 5-C), 122.6 (quinazoline 4a-C), 114.9 (quinoline 5-C), 111.3 (quinoline 7-C), 52.6 (*N*-arylpyrrolidine 2-C, 5-C), 48.6 (*N*-acylpyrrolidine 5-C), 46.2 (*N*-acylpyrrolidine 2-C), 26.2 (*N*-arylpyrrolidine 3-C, 4-C), 26.1 (*N*-acylpyrrolidine 4-C), 25.0 (*N*-acylpyrrolidine 3-C). HRMS (ESI-TOF) *m*/*z* calcd. for C_26_H_26_N_5_O_2_ ([M + H]^+^): 440.2081. Found: 440.2080.

*2-[8-Fluoro-3-(piperidin-1-ylcarbonyl)quinolin-2-yl]quinazolin-4(3H)-one* (**6**). A mixture of the fluoro compound **4** (0.152 g, 0.5 mmol), K_2_CO_3_ (0.069 g, 0.5 mmol) and piperidine (0.426 g, 5 mmol) in DMSO (15 mL) was stirred at 100 °C for 16 h, then it was cooled to room temperature and the volatile components were removed by Kugelrohr distillation (10^−1^ mbar, 100 °C). The residue was taken up in CH_2_Cl_2_ (50 mL) and the solution was washed with water and brine, then dried over Na_2_SO_4_ and evaporated under reduced pressure. The residue was subjected to MPLC, eluting with CH_2_Cl_2_/MeOH (97:3) to afford **6** (0.134 g, 67%) as dark-red crystals, m.p. 215–216 °C (CHCl_3_). MS (EI, 70 eV) *m*/*z* = 402 (M^+^, 2%), 318 (26), 289 (16), 171 (21), 119 (36), 84 (100), 69 (26), 64 (21), 57 (41), 56 (44), 55 (67); ^1^H-NMR (CDCl_3_) δ 11.11 (s, 1H, NH), 8.38 (ddd, *J* = 7.9 Hz, 1.5 Hz, 0.5 Hz, 1H, quinazoline 5-H), 8.24 (d, *J* = 1.4 Hz, 1H, quinoline 4-H), 7.81 (ddd, *J* = 8.4 Hz, 6.9 Hz, 1.5 Hz, 1H, quinazoline 7-H), 7.76 (ddd, *J* = 8.2 Hz, 1.4 Hz, 0.5 Hz, 1H, quinazoline 8-H), 7.70 (dd, *J* = 8.2 Hz, 1.2 Hz, 1H, quinoline 5-H), 7.64 (td, *J* = 7.9 Hz, 4.7 Hz, 1H, quinoline 6-H), 7.59–7.49 (m, 2H, quinazoline 6-H, quinoline 7-H), 4.44–4.27 (m, 1H, piperidine 2-H), 3.50 (ddd, *J* = 13.3 Hz, 9.8 Hz, 3.6 Hz, 1H, piperidine 2′-H), 3.32–3.22 (m, 1H, piperidine 6-H), 3.12 (ddd, *J* = 13.1 Hz, 9.6 Hz, 3.4 Hz, 1H, piperidine 6′-H), 2.01–1.87 (m, 1H, piperidine 3-H), 1.84–1.69 (m, 2H, piperidine 3′-H, 4-H), 1.62–1.50 (m, 2H, piperidine 4′-H, 5-H), 1.48–1.41 (m, 1H, piperidine 5′-H); ^13^C-NMR (CDCl_3_) δ: 168.2 (amide C=O), 161.4, (quinazoline 4-C), 158.1 (d, *J*_C__–F_ = 261.3 Hz, quinoline 8-C), 148.6 (quinazoline 8a-C), 147.9 (quinazoline 2-C), 144.7 (quinoline 2-C), 136.8 (d, *J*_C__–F_ = 12.4 Hz, quinoline 8a-C), 136.0 (d, *J*_C__–F_ = 2.9 Hz, quinoline 4-C), 134.7 (quinazoline 7-C), 131.4 (quinoline 3-C), 130.0 (quinoline 4a-C), 129.3 (d, *J*_C__–F_ = 7.8 Hz, quinoline 6-C), 128.5 (quinazoline 8-C), 128.2 (quinazoline 6-C), 127.1 (quinazoline 5-C), 123.4 (d, *J*_C__–F_ = 5.0 Hz, quinoline 5-C), 123.0 (quinazoline 4a-C), 115.3 (d, *J*_C__–F_ = 18.3 Hz, quinoline 7-C), 48.2 (piperidine 6-C), 43.0 (piperidine 2-C), 25.8 (piperidine 5-C), 25.4 (piperidine 3-C), 24.7 (piperidine 4-C). HRMS (ESI-TOF) *m*/*z* calcd. for C_23_H_20_FN_4_O_2_ ([M + H]^+^) 403.1563. Found 403.1563.

*2-[8-Fluoro-3-(morpholin-4-ylcarbonyl)quinolin-2-yl]quinazolin-4(3H)-one* (**7**). A mixture of the fluoro compound **4** (0.152 g, 0.5 mmol), K_2_CO_3_ (0.069 g, 0.5 mmol) and morpholine (0.436 g, 5 mmol) in DMSO (15 mL) was stirred at 100 °C for 16 h, then it was cooled to room temperature and the volatile components were removed by Kugelrohr distillation (10^−1^ mbar, 100 °C). The residue was taken up in CH_2_Cl_2_ (50 mL) and the solution was washed with water and brine, then dried over Na_2_SO_4_ and evaporated under reduced pressure. The residue was subjected to column chromatography, eluting with CH_2_Cl_2_/MeOH (97:3) to afford **7** (0.067 g, 33%) as yellow crystals, m.p. > 265 °C (sublimation; CHCl_3_). MS (EI, 70 eV) *m*/*z* = 405 ([M + 1]^+^, 2%), 404 (M^+^, 8), 319 (85), 318 (100), 146 (20), 119 (43), 57 (22), 56 (23); ^1^H-NMR (CDCl_3_) δ 11.10 (s, 1H, NH), 8.41–8.36 (m, 1H, quinazoline 5-H), 8.28 (d, *J* = 1.4 Hz, 1H, quinoline 4-H), 7.82 (ddd, *J* = 8.4 Hz, 7.0 Hz, 1.5 Hz, 1H, quinazoline 7-H), 7.77 (ddd, *J* = 8.2 Hz, 1.4 Hz, 0.6 Hz, 1H, quinazoline 8-H), 7.72 (dd, *J* = 8.3 Hz, 1.3 Hz, 1H, quinoline 5-H), 7.66 (td, *J* = 7.9 Hz, 4.6 Hz, 1H, quinoline 6-H), 7.61–7.56 (m, 1H, quinazoline 6-H), 7.56–7.51 (m, 1H, quinoline 7-H), 4.29 (dt, *J* = 13.0 Hz, 3.0 Hz, 1H, morpholine 5-H), 4.11 (dt, *J* = 11.7 Hz, 3.6 Hz, 1H, morpholine 6-H), 3.81 (ddd, *J* = 11.7 Hz, 9.3 Hz, 2.9 Hz, 1H, morpholine 6′-H), 3.74–3.64 (m, 2H, morpholine 2-H, 5′-H), 3.55 (ddd, *J* = 11.6 Hz, 9.0 Hz, 3.1 Hz, 1H, morpholine 2′-H), 3.38–3.27 (m, 1H, morpholine 3-H), 3.26–3.17 (m, 1H, morpholine 3′-H); ^13^C-NMR (CDCl_3_) δ: 168.8 (amide C=O), 161.3 (quinazoline 4-C), 158.0 (*J*_C__–F_ = 260.0 Hz, quinoline 8-C), 148.3 (quinazoline 8a-C), 147.8 (quinazoline 2-C), 144.7 (quinoline 2-C), 136.9 (*J*_C__–F_ = 12.5 Hz, quinoline 8a-C), 136.4 (*J*_C__–F_ = 2.8 Hz, quinoline 4-C), 134.9 (quinazoline 7-C), 130.2 (quinoline 3-C), 129.8 (quinoline 4a-C), 129.6 (*J*_C__–F_ = 7.9 Hz, quinoline 6-C), 128.5 (quinazoline 8-C), 128.4 (quinazoline 6-C), 127.1 (quinazoline 5-C), 123.5 (*J*_C__–F_ = 4.9 Hz, quinoline 5-C), 122.9 (quinazoline 4a-C), 115.6 (*J*_C__–F_ = 18.4 Hz, quinoline 7-C), 66.6 (morpholine 2-C), 66.2 (morpholine 6-C), 47.2 (morpholine 3-C), 42.6 (morpholine 5-C). HRMS (ESI-TOF) *m*/*z* calcd. for C_22_H_18_FN_4_O_3_ ([M + H]^+^) 405.1357. Found 405.1356.

*N-Butyl-8-fluoro-2-(4-oxo-3,4-dihydroquinazolin-2-yl)quinoline-3-carboxamide* (**8**). A mixture of the fluoro compound **4** (0.100 g, 0.33 mmol), K_2_CO_3_ (0.046 g, 0.33 mmol) and *n*-butylamine (0.241 g, 3.3 mmol) in DMSO (10 mL) was stirred at 100 °C for 16 h, then it was cooled to room temperature and the volatile components were removed by Kugelrohr distillation (10^−1^ mbar, 100 °C). The residue was taken up in CH_2_Cl_2_ (50 mL) and the solution was washed with water and brine, then dried over Na_2_SO_4_ and evaporated under reduced pressure. The residue was subjected to MPLC, eluting with CH_2_Cl_2_/MeOH (96:4) to afford **8** (0.078 g, 53%) as colorless crystals, m.p. 218–220 °C (methyl tert-butyl ether). MS (EI, 70 eV) *m*/*z* = 391 ([M + 1]^+^, 2%), 390 (M^+^, 6), 319 (63), 318 (100), 291 (28), 171 (20), 146 (21), 119 (49), 92 (22), 90 (35); ^1^H-NMR (CDCl_3_) δ 10.94 (s, 1H, quinazolinone NH), 8.39 (d, *J* = 1.3 Hz, 1H, quinoline 4-H), 8.22 (d, *J* = 7.9 Hz, 1H, quinazoline 5-H), 7.76–7.74 (m, 2H, quinazoline 7-H, 8-H), 7.68 (d, *J* = 7.6 Hz, 1H, quinoline 5-H), 7.62 (td, *J* = 7.9 Hz, 4.8 Hz, 1H, quinoline 6-H), 7.53–7.46 (m, 1H, quinoline 7-H), 7.46–7.38 (m, 1H, quinazoline 6-H), 6.54 (t, *J* = 5.6 Hz, 1H, amide NH), 3.71–3.58 (m, 2H, butyl 1-CH_2_), 1.76 (p, *J* = 7.5 Hz, 2H, butyl 2-CH_2_), 1.51 (dq, *J* = 14.6 Hz, 7.3 Hz, 2H, butyl 3-CH_2_), 1.01 (t, *J* = 7.4 Hz, 3H, butyl CH_3_); ^13^C-NMR (CDCl_3_) δ: 168.1 (amide C=O), 161.2 (quianzoline 4-C), 157.7 (d, *J*_C__–F_ = 261.3 Hz, quinoline 8-C), 148.2 (quinazoline 8a-C), 147.6 (quinazoline 2-C), 144.6 (quinoline 2-C), 137.6 (d, *J*_C__–F_ = 2.5 Hz, quinoline 4-C), 136.6 (d, *J*_C__–F_ = 12.4 Hz, quinoline 8a-C), 134.6 (quinazoline 7-C), 131.5 (quinoline 3-C), 129.4 (quinoline 4a-C), 129.2 (d, *J*_C__–F_ = 7.9 Hz, quinoline 6-C), 128.3 (quinazoline 8-C), 127.9 (quinazoline 6-C), 126.6 (quinazoline 5-C), 123.4 (d, *J*_C__–F_ = 4.8 Hz, quinoline 5-C), 122.4 (quinazoline 4a-C), 115.4 (d, *J*_C__–F_ = 18.3 Hz, quinoline 7-C), 40.5 (butyl 1-C), 31.5 (butyl 2-C), 20.4 (butyl 3-C), 13.9 (butyl 4-C). HRMS (ESI-TOF) *m*/*z* calcd. for C_22_H_20_FN_4_O_2_ ([M + H]^+^) 391.1565. Found 391.1561.

*2-[3-(Morpholin-4-ylcarbonyl)quinolin-2-yl]quinazolin-4(3H)-one* (**10**). A mixture of Luotonin A (**9)** (0.029 g, 0.1 mmol), K_2_CO_3_ (0.014 g, 0.1 mmol) and morpholine (0.087 g, 1 mmol) in DMSO (5 mL) was stirred at 100 °C for 16 h, then it was cooled to room temperature and the volatile components were removed by Kugelrohr distillation (10^−1^ mbar, 100 °C). The residue was taken up in CH_2_Cl_2_ (20 mL) and the solution was washed with water and brine, then dried over Na_2_SO_4_ and evaporated under reduced pressure to afford **10** (0.020 g, 52%) as almost colorless crystals, m.p. > 272 °C (sublimation; CH_2_Cl_2_). MS (EI, 70 eV) *m*/*z* = 296 (21%), 211 (27), 111 (32), 109 (24), 97 (47), 95 (39), 85 (38), 83 (46), 81 (51), 71 (56), 69 (81), 67 (28), 57 (100); ^1^H-NMR (CDCl_3_) δ 11.15 (s, 1H, NH), 8.38 (dd, *J* = 7.9 Hz, 1.0 Hz, 1H, quinazoline 5-H), 8.25 (s, 1H, quinoline 4-H), 8.20 (d, *J* = 7.5 Hz, 1H, quinoline 8-H), 7.92 (d, *J* = 8.1 Hz, 1H, quinoline 5-H), 7.88 (ddd, *J* = 8.4 Hz, 6.9 Hz, 1.4 Hz, 1H, quinoline 7-H), 7.85–7.75 (m, 2H, quinazoline 7-H, 8-H), 7.72 (ddd, *J* = 8.2 Hz, 7.0 Hz, 1.2 Hz, 1H, quinoline 6-H), 7.57 (ddd, *J* = 8.2 Hz, 6.9 Hz, 1.5 Hz, 1H, quinazoline 6-H), 4.29 (dt, *J* = 13.4 Hz, 3.5 Hz, 1H, morpholine 3-H), 4.10 (dt, *J* = 11.6 Hz, 3.7 Hz, 1H, morpholine 2-H), 3.86–3.76 (m, 1H, morpholine 2′-H), 3.74–3.63 (m, 2H, morpholine 3′-H, 6-H), 3.55 (ddd, *J* = 11.7 Hz, 8.8 Hz, 3.2 Hz, 1H, morpholine 6′-H), 3.32–3.24 (m, 2H, morpholine 5-H, 5′-H); ^13^C-NMR (CDCl_3_) δ: 169.2 (amide C=O), 161.4 (quinazoline 4-C), 148.6 (quinazoline 8a-C), 148.1 (quinazoline 2-C), 146.5 (quinoline 8a-C), 144.3 (quinoline 2-C), 136.6 (quinoline 4-C), 134.9 (quinazoline 7-C), 131.7 (quinoline 7-C), 129.8 (quinoline 8-C), 129.5 (quinoline 6-C), 129.2 (quinoline 3-C), 128.5 (quinazoline 8-C), 128.4 (quinoline 4a-C), 128.2 (quinazoline 6-C), 127.9 (quinoline 5-C), 127.0 (quinazoline 5-C), 122.8 (quinazoline 4a-C), 66.6 (morpholine 2-C), 66.3 (morpholine 6-C), 47.3 (morpholine 5-C), 42.6 (morpholine 3-C), HRMS (ESI-TOF) *m*/*z* calcd. for C_22_H_19_N_4_O_3_ ([M + H]^+^) 387.1452. Found 387.1451.

*N-Butyl-2-(4-oxo-3,4-dihydroquinazolin-2-yl)quinoline-3-carboxamide* (**11**). A mixture of Luotonin A (**9)** (0.057 g, 0.2 mmol), K_2_CO_3_ (0.028 g, 0.2 mmol) and *n*-butylamine (0.146 g, 2 mmol) in DMSO (7 mL) was stirred at 100 °C for 16 h, then it was cooled to room temperature and the volatile components were removed by Kugelrohr distillation (10^−1^ mbar, 100 °C). The residue was taken up in CH_2_Cl_2_ (30 mL) and the solution was washed with water and brine, then dried over Na_2_SO_4_ and evaporated under reduced pressure to afford **11** (0.052 g, 70%) as orange crystals, m.p. > 227 °C (sublimation; CH_2_Cl_2_). MS (EI, 70 eV) *m*/*z* = 373 ([M + 1]^+^, 2%), 372 (M^+^, 6), 329 (9), 301 (63), 300 (100), 273 (29), 153 (22), 119 (33), 90 (23); ^1^H-NMR (CDCl_3_) δ 11.00 (s, 1H, quinazolinone NH), 8.37 (s, 1H, quinoline 4-H), 8.27 (d, *J* = 8.2 Hz, 1H, quinazoline 5-H), 8.10 (d, *J* = 8.4 Hz, 1H, quinoline 8-H), 7.88 (d, *J* = 8.1 Hz, 1H, quinoline 5-H), 7.86–7.80 (m, 1H, quinoline 7-H), 7.79–7.71 (m, 2H, quinazoline 7-H, 8-H), 7.67 (t, *J* = 7.5 Hz, 1H, quinoline 6-H), 7.48 (ddd, *J* = 8.1 Hz, 6.2 Hz, 2.1 Hz, 1H, quinazoline 6-H), 6.50 (t, *J* = 5.2 Hz, 1H, amide NH), 3.69–3.57 (m, 2H, butyl 1-CH_2_), 1.80–1.68 (m, 2H, butyl 2-CH_2_), 1.49 (dq, *J* = 14.8 Hz, 7.4 Hz, 2H, butyl 3-CH_2_), 1.00 (t, *J* = 7.4 Hz, 3H, CH_3_); ^13^C-NMR (CDCl_3_) δ: 168.6 (amide C=O), 161.5 (quinazoline 4-C), 148.5 (quinazoline 8a-C), 148.1 (quinazoline 2-C), 146.4 (quinoline 8a-C), 144.5 (quinoline 2-C), 137.9 (quinoline 4-C), 134.7 (quinazoline 7-C), 131.6 (quinoline 7-C), 130.6 (quinoline 3-C), 129.6 (quinoline 8-C), 129.3 (quinoline 6-C), 128.4 (quinazoline 8-C), 128.1 (quinoline 4a-C), 128.0 (quinazoline 6-C), 127.9 (quinoline 5-C), 126.8 (quinazoline 5-C), 122.6 (quinazoline 4a-C), 40.6 (butyl 1-C), 31.6 (butyl 2-C), 20.5 (butyl 3-C), 14.0 (butyl 4-C). HRMS (ESI-TOF) *m*/*z* calcd. for C_22_H_20_N_4_NaO_2_ ([M + Na]^+^) 395.1478. Found 395.1476.

*N-[2-(Dimethylamino)ethyl]-2-(4-oxo-3,4-dihydroquinazolin-2-yl)quinoline-3-carboxamide* (**12**). A mixture of Luotonin A (**9)** (0.100 g, 0.35 mmol), K_2_CO_3_ (0.048 g, 0.35 mmol) and *N*,*N*-dimethyl­ethylene­diamine (0.309 g, 3.5 mmol) in DMSO (10 mL) was stirred at 100 °C for 16 h, then it was cooled to room temperature and the volatile components were removed by Kugelrohr distillation (10^−1^ mbar, 100 °C). The residue was taken up in CH_2_Cl_2_ (50 mL) and the solution was washed with water and brine, dried over Na_2_SO_4_ and evaporated under reduced pressure to afford **12** as crude product (0.070 g, 52%). The residue was subjected to MPLC, eluting with CH_2_Cl_2_/methanol (80 + 20) to afford **12** as colorless crystals, m.p. 185–188 °C (CH_2_Cl_2_). MS (EI, 70 eV) *m*/*z* = 197 (5%), 155 (18), 137 (46), 110 (34), 108 (12), 83 (14), 82 (13), 57 (13), 43 (100); ^1^H-NMR (CDCl_3_) δ 11.15 (br s, 1H, quinazolinone NH), 8.37 (s, 1H, quinoline 4-H), 8.35 (ddd, *J* = 8.0 Hz, 1.2 Hz, 0.6 Hz, 1H, quinazoline 5-H), 8.15 (d, *J* = 8.4 Hz, 1H, quinoline 8-H), 7.88 (d, *J* = 8.1 Hz, 1H, quinoline 5-H), 7.84 (ddd, *J* = 8.4 Hz, 6.9 Hz, 1.4 Hz, 1H, quinoline 7-H), 7.80–7.73 (m, 2H, quinazoline 7-H, 8-H), 7.67 (ddd, *J* = 8.1 Hz, 7.0 Hz, 1.1 Hz, 1H, quinoline 6-H), 7.52 (ddd, *J* = 8.2 Hz, 6.3 Hz, 2.0 Hz, 1H, quinazoline 6-H), 6.81 (br s, 1H, amide NH), 3.72 (q, *J* = 5.7 Hz, 2H, 1-CH_2_), 2.62 (t, *J* = 5.7 Hz, 2H, 2-CH_2_), 2.19 (s, 6H, CH_3_); ^13^C-NMR (CDCl_3_) δ: 168.8 (amide C=O), 161.5 (quinazoline 4-C), 148.6 (quinazoline 8a-C), 148.2 (quinazoline 2-C), 146.5 (quinoline 8a-C), 144.7 (quinoline 2-C), 137.7 (quinoline 4-C), 134.7 (quinazoline 7-C), 131.6 (quinoline 7-C), 130.5 (quinoline 3-C), 129.6 (quinoline 8-C), 129.2 (quinoline 6-C), 128.4 (quinazoline 8-C), 128.1 (quinaoline 4a-C), 128.0 (quinoline 5-C or quinazoline 6-C), 127.9 (quinazoline 6-C or quinoline 5-C), 126.9 (quinazoline 5-C), 122.7 (quinazoline 4a-C), 57.5 (2-CH_2_), 45.1 (CH_3_), 37.9 (1-C7H_2_). HRMS (ESI-TOF) *m*/*z* calcd. for C_22_H_22_N_5_O_2_ ([M + H]^+^): 388.1768. Found: 388.1772.

*2-[5,7-Dimethoxy-3-(morpholin-4-ylcarbonyl)quinolin-2-yl]quinazolin-4(3H)-one* (**14**). A mixture of 1,3-dimethoxyquinolino[2′,3′:3,4]pyrrolo[2,1-b]quinazolin-11(13*H*)-one (**13**) (0.172 g, 0.5 mmol), K_2_CO_3_ (0.069 g, 0.5 mmol) and morpholine (0.436 g, 5 mmol) in DMSO (15 mL) was stirred at 100 °C for 16 h, then it was cooled to room temperature and the volatile components were removed by Kugelrohr distillation (10^−1^ mbar, 100 °C). The residue was taken up in CH_2_Cl_2_ (50 mL) and the solution was washed with water and brine, then dried over Na_2_SO_4_ and evaporated under reduced pressure. The residue was subjected to MPLC, eluting with CH_2_Cl_2_/methanol (95 + 5) to afford **14** (0.060 g, 27%) as yellowish crystals, m.p. > 264 °C (sublimation; CH_2_Cl_2_). MS (EI, 70 eV) *m*/*z* = 447 ([M + 1]^+^, 2%), 446 (M^+^, 8) 361 (78), 360 (100), 333 (42), 302 (22), 119 (26), 90 (32), 86 (25), 57 (30), 56 (57), 55 (27); ^1^H-NMR (CDCl_3_) δ 11.14 (s, 1H, NH), 8.50 (d, *J* = 0.5 Hz, 1H, quinoline 4-H), 8.37 (dd, *J* = 8.0 Hz, 1.1 Hz, 1H, quinazoline 5-H), 7.80 (ddd, *J* = 8.3 Hz, 6.8 Hz, 1.5 Hz, 1H, quinazoline 7-H), 7.76 (dd, *J* = 8.1 Hz, 1.0 Hz, 1H, quinazoline 8-H), 7.55 (ddd, *J* = 8.2 Hz, 6.9 Hz, 1.5 Hz, 1H, quinazoline 6-H), 7.06 (d, *J* = 1.6 Hz, 1H, quinoline 8-H), 6.63 (d, *J* = 2.1 Hz, 1H, quinoline 6-H), 4.29–4.24 (m, 1H, morpholine 3-H), 4.11–4.06 (m, 1H, morpholine 2-H), 4.02 (s, 3H, 7-OCH_3_), 4.01 (s, 3H, 5-OCH_3_), 3.84–3.77 (m, 1H, morpholine 2′-H), 3.73–3.66 (m, 2H, morpholine 3′-H, 6-H), 3.59–3.52 (m, 1H, morpholine 6′-H), 3.31–3.23 (m, 2H, morpholine 5-H, 5′-H); ^13^C-NMR (CDCl_3_) δ: 169.7 (amide C=O), 163.1 (quinoline 7-C), 161.4 (quinazoline 4-C), 156.1 (quinoline 5-C), 148.9 (quinoline 8a-C), 148.7 (quinazoline 8a-C), 148.3 (quinazoline 2-C), 144.6 (quinoline 2-C), 134.8 (quinazoline 7-C), 131.7 (quinoline 4-C), 128.5 (quinazoline 8-C), 128.0 (quinazoline 6-C), 127.0 (quinazoline 5-C), 126.0 (quinoline 3-C), 122.8 (quinazoline 4a-C), 117.4 (quinoline 4a-C), 100.7 (quinoline 6-C), 99.5 (quinoline 8-C), 66.7 (morpholine 2-C), 66.4 (morpholine 6-C), 56.2 (7-OCH_3_), 56.0 (5-OCH_3_), 47.3 (morpholine 5-C), 42.6 (morpholine 3-C). HRMS (ESI-TOF) *m*/*z* calcd. for C_24_H_23_N_4_O_5_ ([M + H]^+^) 447.1663. Found 447.1662.

### 3.3. Biological Evaluation

Human colon adenocarcinoma cells (SW480) were seeded in a 96-well plate at a density of 5 × 10^4^ cells per mL. Minimum Essential Medium Eagle (MEM) with 10% FCS (Fetal Calf Serum) was used to conduct the culture in the first 24 h at 37 °C in the presence of 5% CO_2_. Compounds were then added in defined concentrations to the cultivated cells using DMSO as solvent and diluting this solution with serum-free MEM-BSA (Minimum Essential Medium Eagle, Bovine Serum Albumin). Treated cells were incubated for further 72 h at 37 °C and an atmosphere containing 5% CO_2_. After this incubation period, the MTT viability assay [32] was performed. This test measures cell viability using the redox potential of living cells. Functional cells reduce a yellow-colored tetrazolium salt to a red formazan derivative, which can be then determined by optical density. After 3 h of incubation, the absorbance of formazan was measured at 450 nm with 620 nm as a reference wavelength.

## 4. Conclusions

In summary, we have discovered an unprecedented oxidative ring opening reaction of the central ring C in Luotonin A and two of its derivatives simply on heating with an excess amine and potassium carbonate in DMSO solution in the presence of air oxygen. The amide-type reaction products were fully characterized and all ^1^H- and ^13^C-NMR signals have been assigned by a combination of HSQC, HMBC, COSY, and NOESY experiments. Four of the new compounds show moderate in vitro anticancer activity towards human colon adenocarcinoma cells.

## Figures and Tables

**Table 1:** In vitro cell growth inhibition (%) of compounds **5**–**8**, **10**–**12** and **14** towards SW480 cancer cells at a fixed concentration of 40 μM (MTT viability assay [32]).

Compound	Luotonin A	5	6	7	8	10	11	12	14
% Inhibition	19 ± 3	47 ± 3	54 ± 10	52 ± 7	no inhib.	22 ± 1	5 ± 4	3 ± 0	7 ± 9

## Supplementary Materials

Spectra (MS, ^1^H-NMR, ^13^C-NMR) of all new compounds are available online.
