# Supplementary material for: A Facile Oxidative Opening of the C-Ring in Luotonin A and Derivatives
Source: Molecules. 2017 Sep 12;22(9):1540. doi: 10.3390/molecules22091540 (PMC6151605; doi:10.3390/molecules22091540)
Supplement: Supplementary file 1 [file molecules-22-01540-s001.pdf]

An unexpected oxidative opening of the C-ring in Luotonin A and derivatives

Amra Ibric [1], Kathrin Dutter [1], Brigitte Marian [2] and Norbert Haider [1,*]

[1] Department of Pharmaceutical Chemistry, University of Vienna, Althanstraße 14, A-1090 Vienna, Austria; amra.ibric@univie.ac.at (A.I.) ; kathrin_dutter@gmx.at (K.D.) ; norbert.haider@univie.ac.at (N.H.)

[2] Institute of Cancer Research, Medical University of Vienna, Borschkegasse 8a, A-1090 Vienna, Austria; brigitte.marian@meduniwien.ac.at

* Correspondence: norbert.haider@univie.ac.at; Tel.: +43-1-4277-55624

Supplementary Material

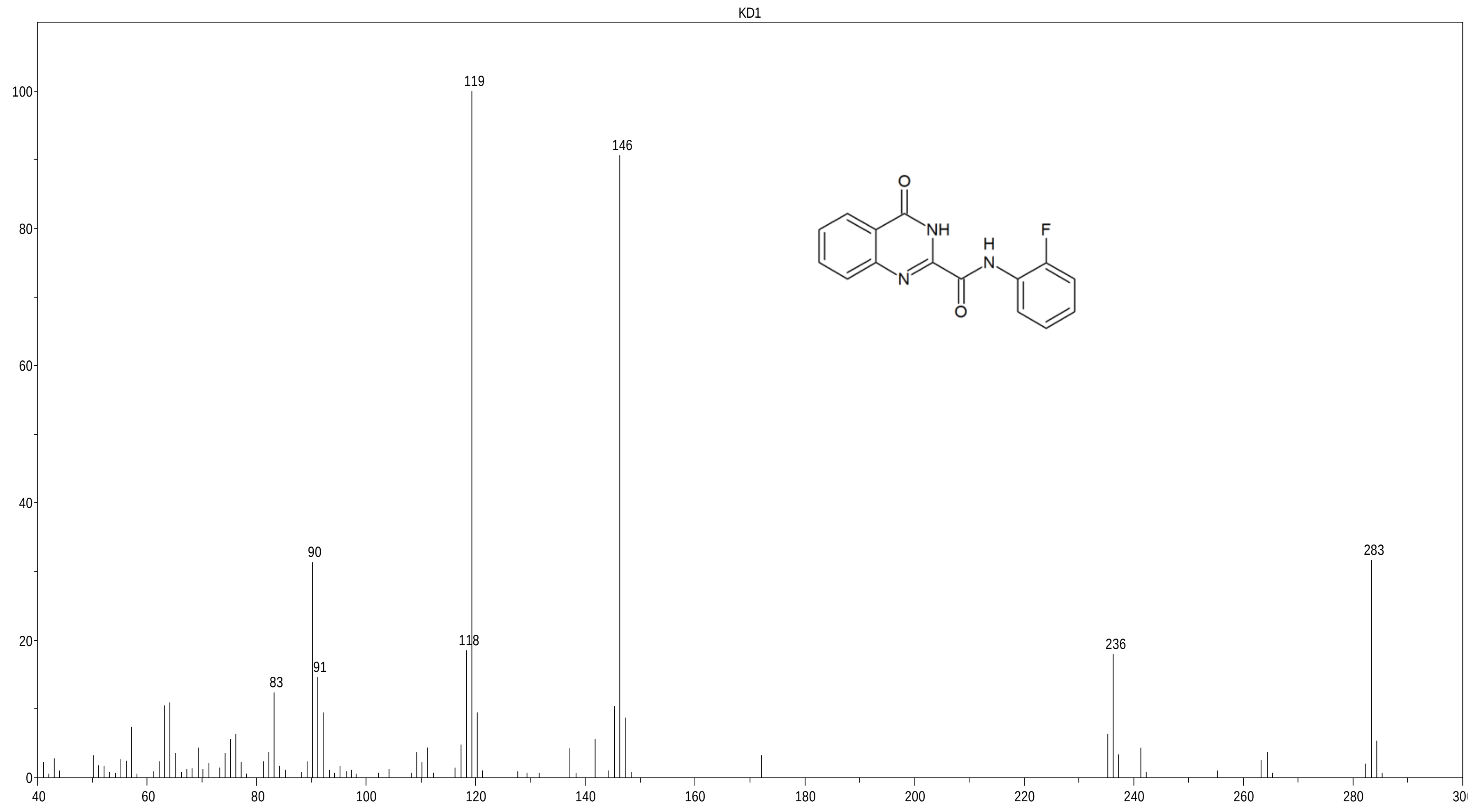

Figure S1. EI-MS of *N*-(2-fluorophenyl)-4-oxo-3,4-dihydroquinazoline-2-carboxamide (**2**)

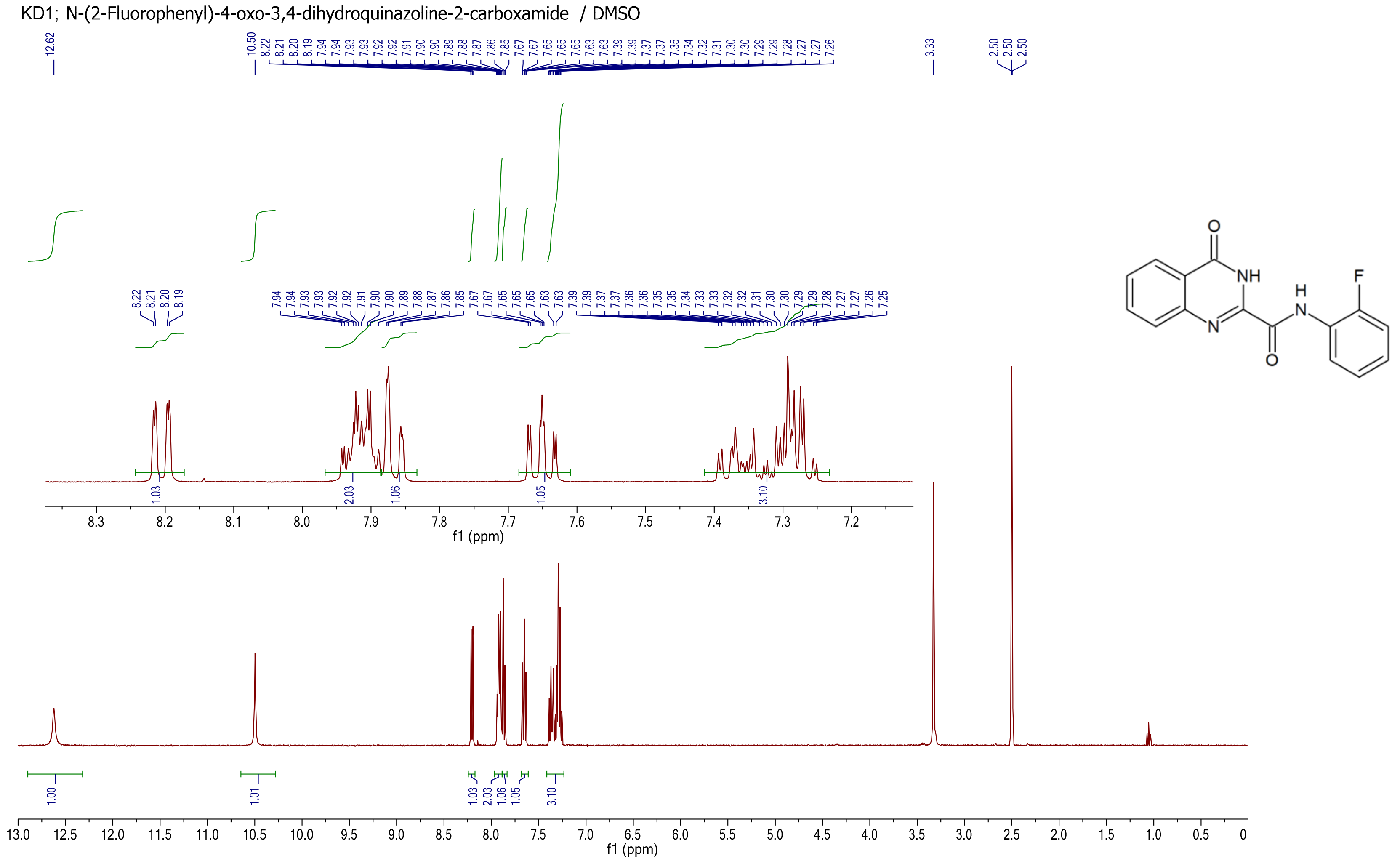

Figure S2. ^{1}H-NMR spectrum of *N*-(2-fluorophenyl)-4-oxo-3,4-dihydroquinazoline-2-carboxamide (**2**)

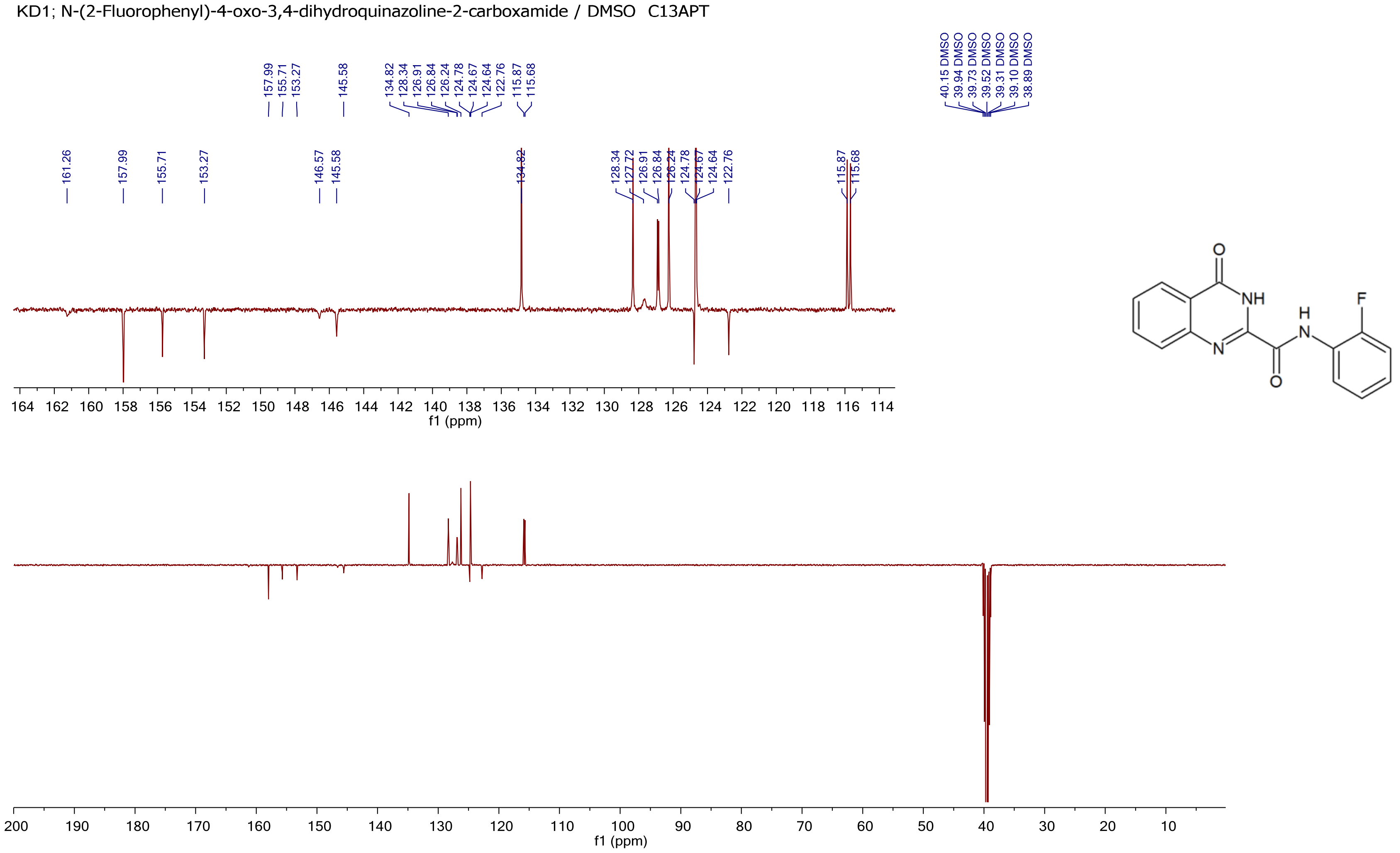

Figure S3. ^{13}C-NMR spectrum of *N*-(2-fluorophenyl)-4-oxo-3,4-dihydroquinazoline-2-carboxamide (**2**)

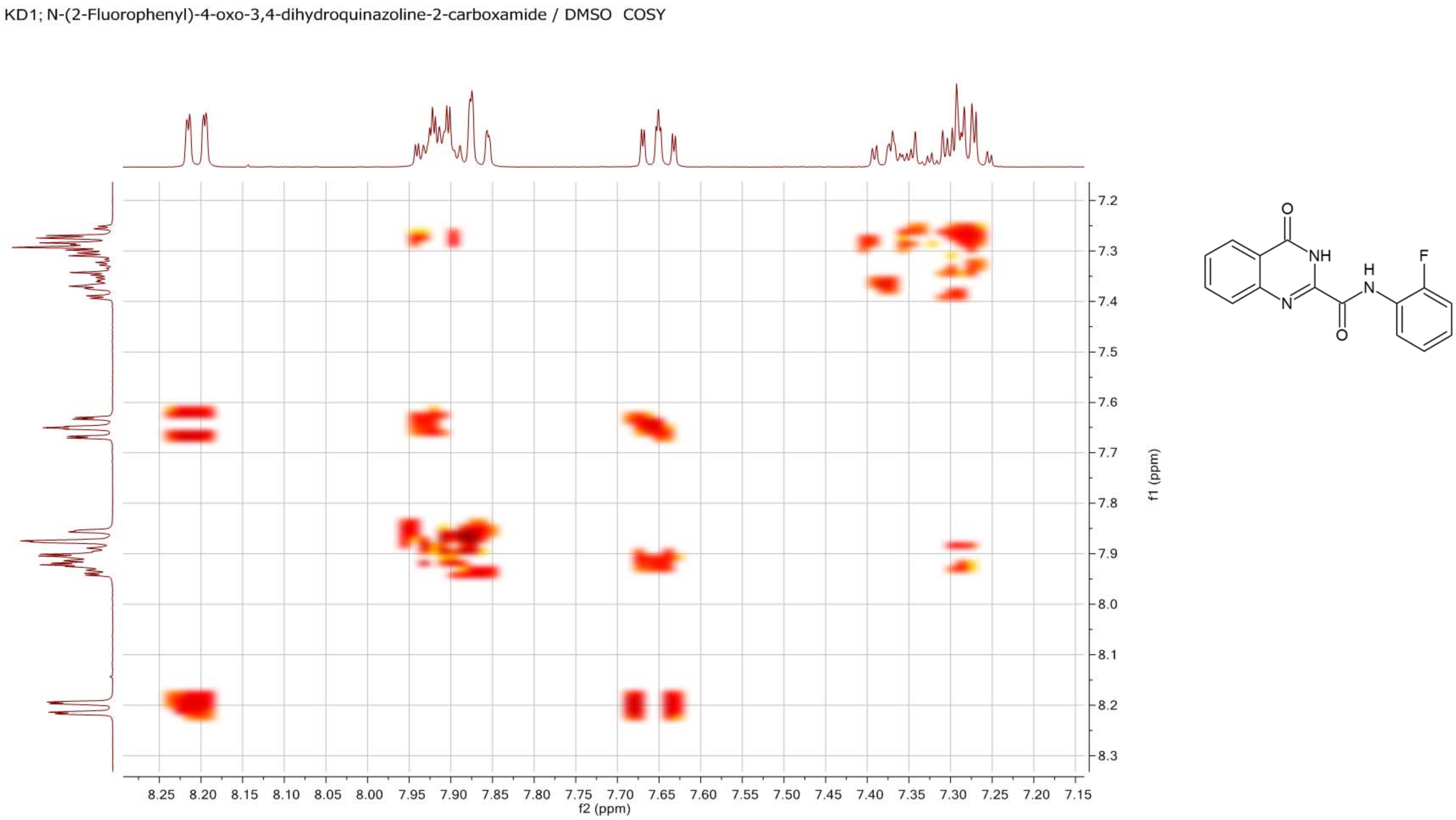

Figure S4. COSY spectrum of *N*-(2-fluorophenyl)-4-oxo-3,4-dihydroquinazoline-2-carboxamide (**2**)

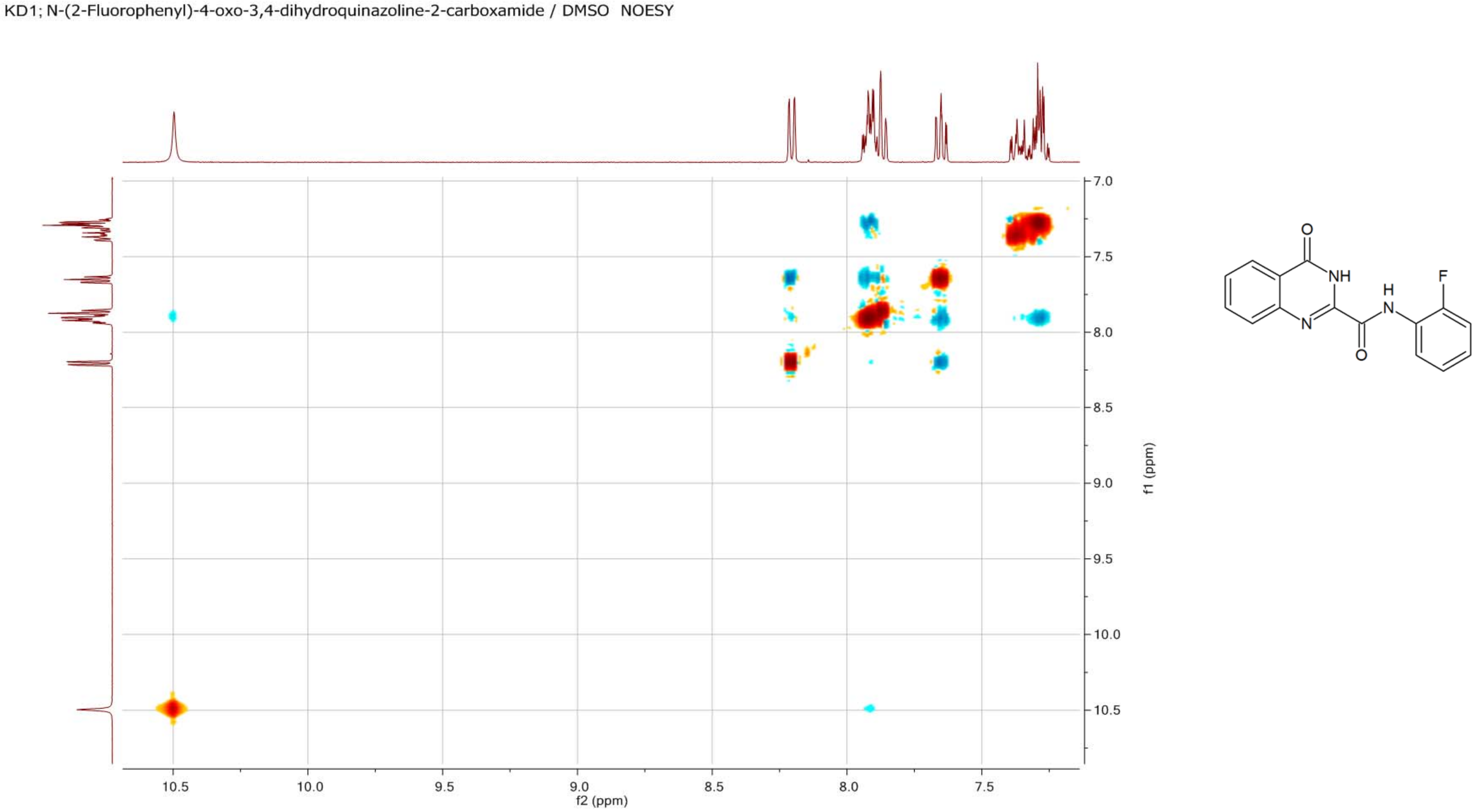

Figure S5. NOESY spectrum of *N*-(2-fluorophenyl)-4-oxo-3,4-dihydroquinazoline-2-carboxamide (**2**)

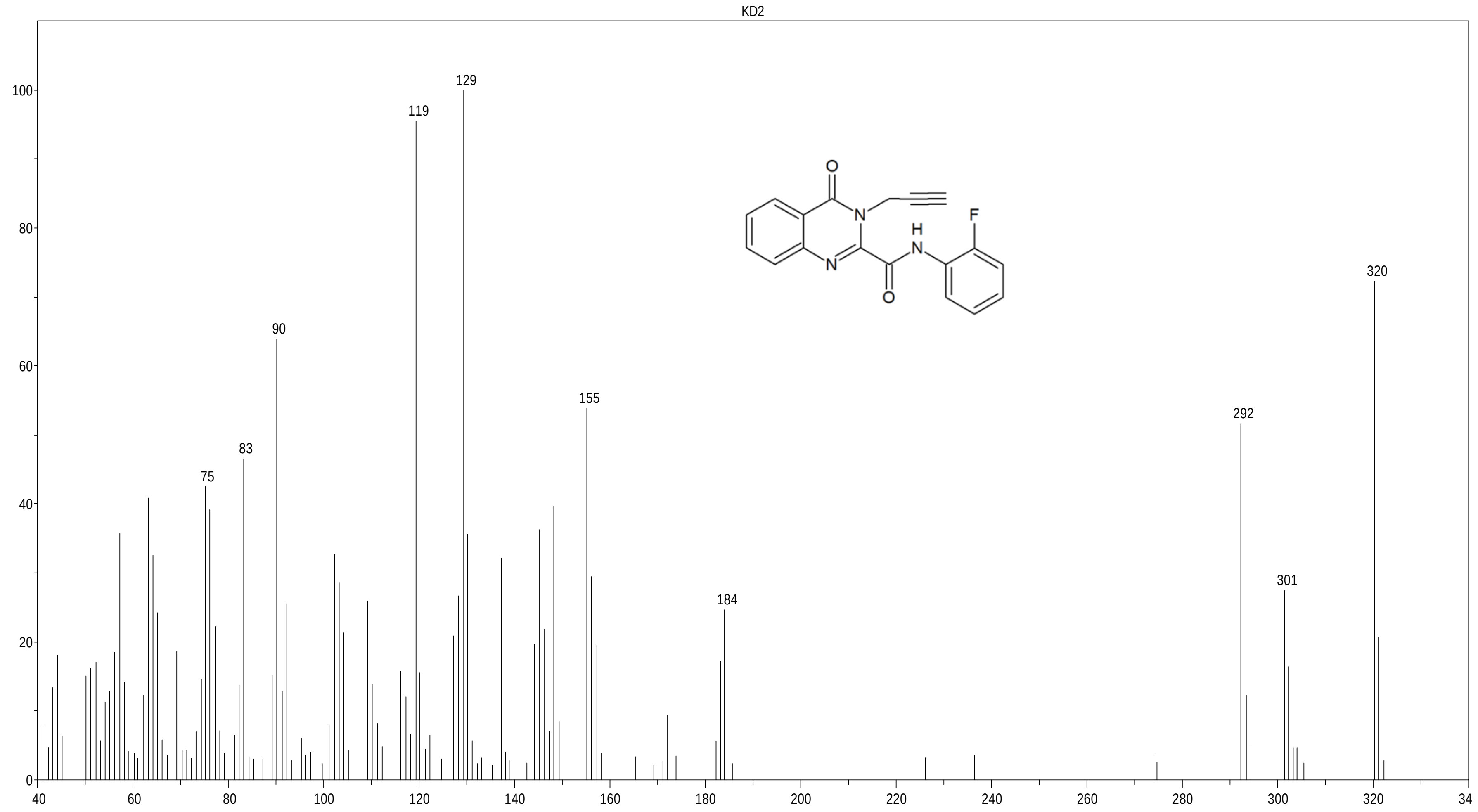

Figure S6. EI-MS of *N*-(2-fluorophenyl)-4-oxo-3-(prop-2-yn-1-yl)-3,4-dihydroquinazoline-2-carboxamide (**3**)

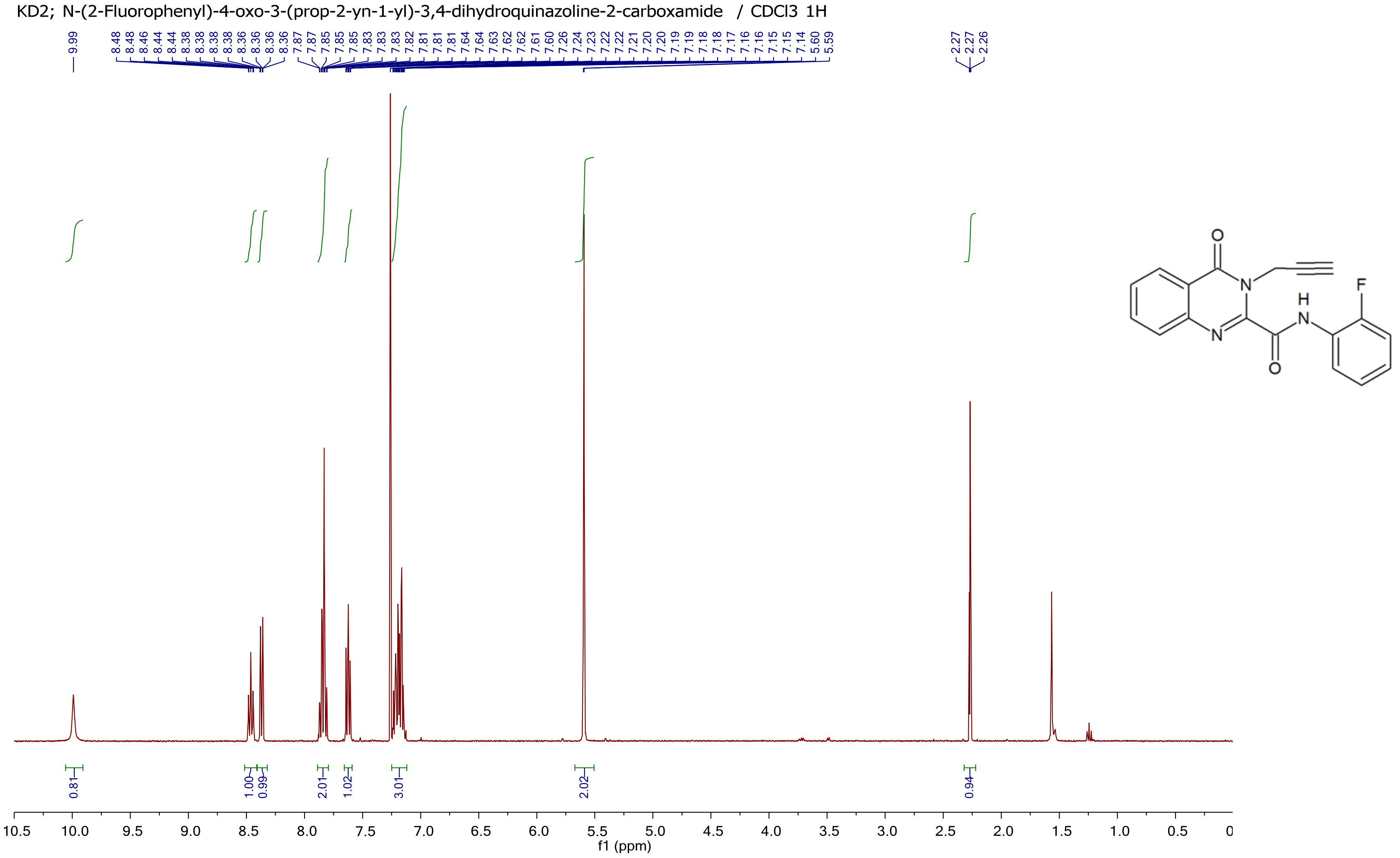

Figure S7. ^{1}H-NMR spectrum of *N*-(2-fluorophenyl)-4-oxo-3-(prop-2-yn-1-yl)-3,4-dihydroquinazoline-2-carboxamide (**3**)

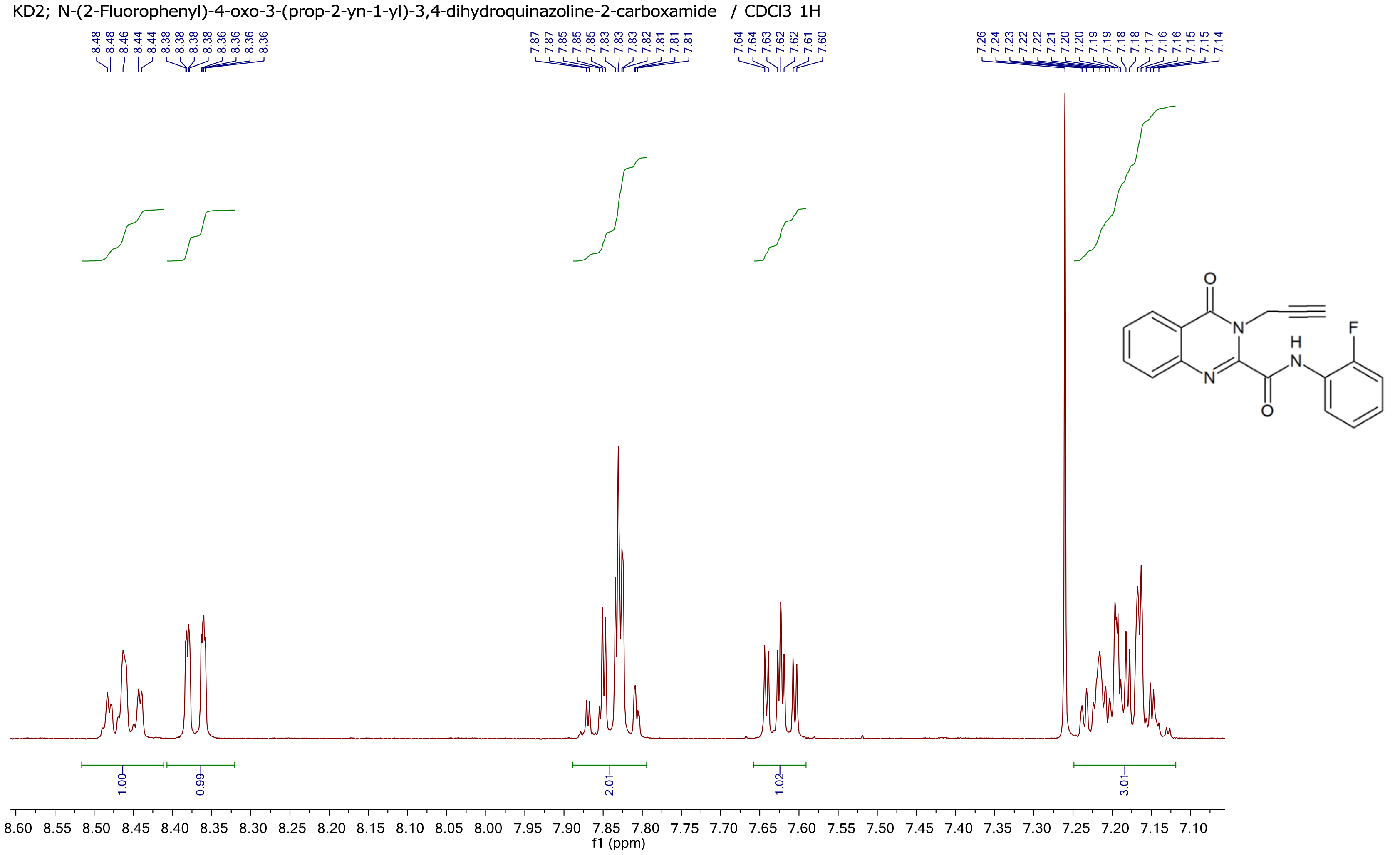

Figure S8. ^{1}H-NMR spectrum of *N*-(2-fluorophenyl)-4-oxo-3-(prop-2-yn-1-yl)-3,4-dihydroquinazoline-2-carboxamide (**3**)

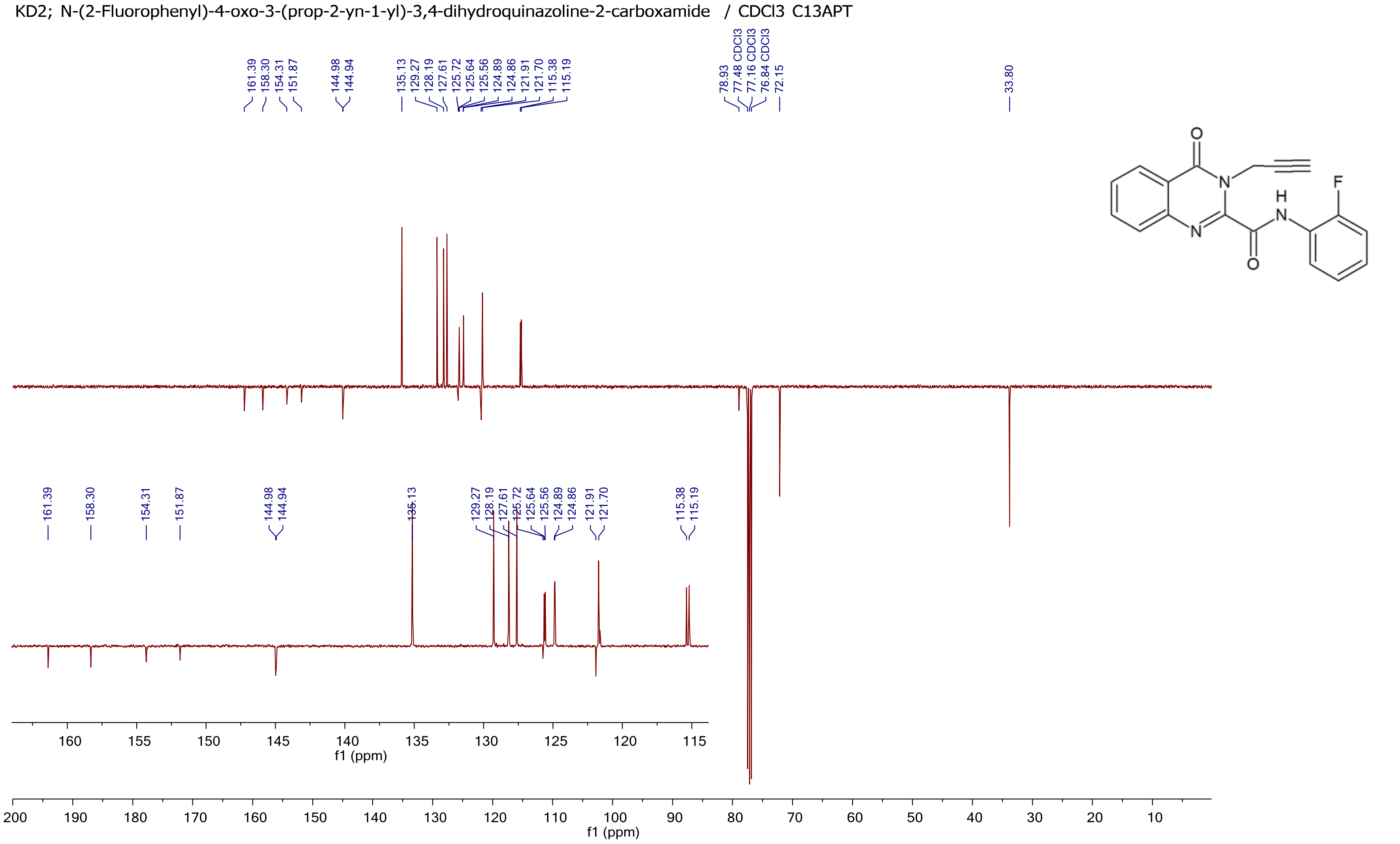

Figure S9. ^{13}C-NMR spectrum of *N*-(2-fluorophenyl)-4-oxo-3-(prop-2-yn-1-yl)-3,4-dihydroquinazoline-2-carboxamide (**3**)

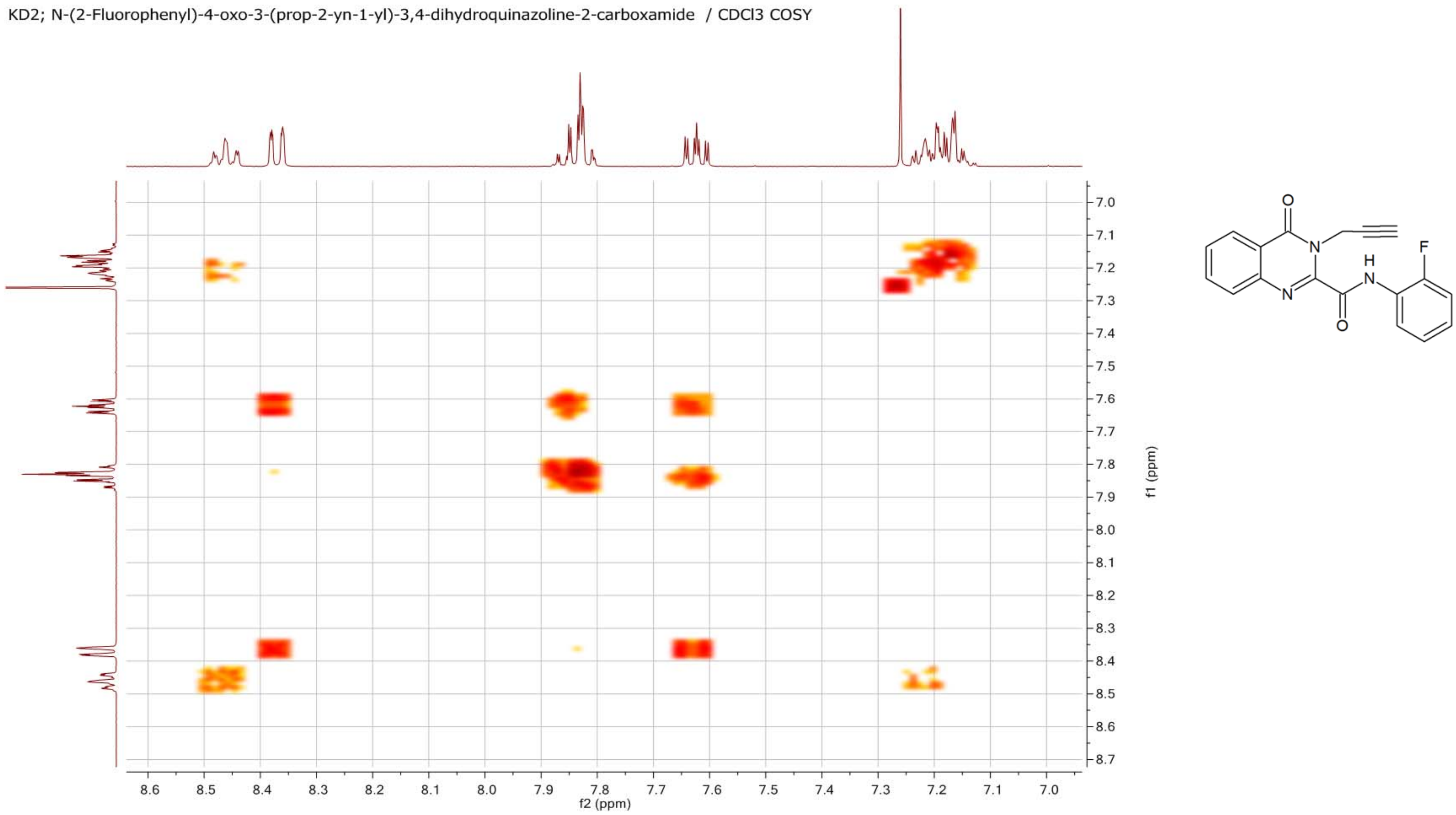

Figure S10. COSY spectrum of *N*-(2-fluorophenyl)-4-oxo-3-(prop-2-yn-1-yl)-3,4-dihydroquinazoline-2-carboxamide (**3**)

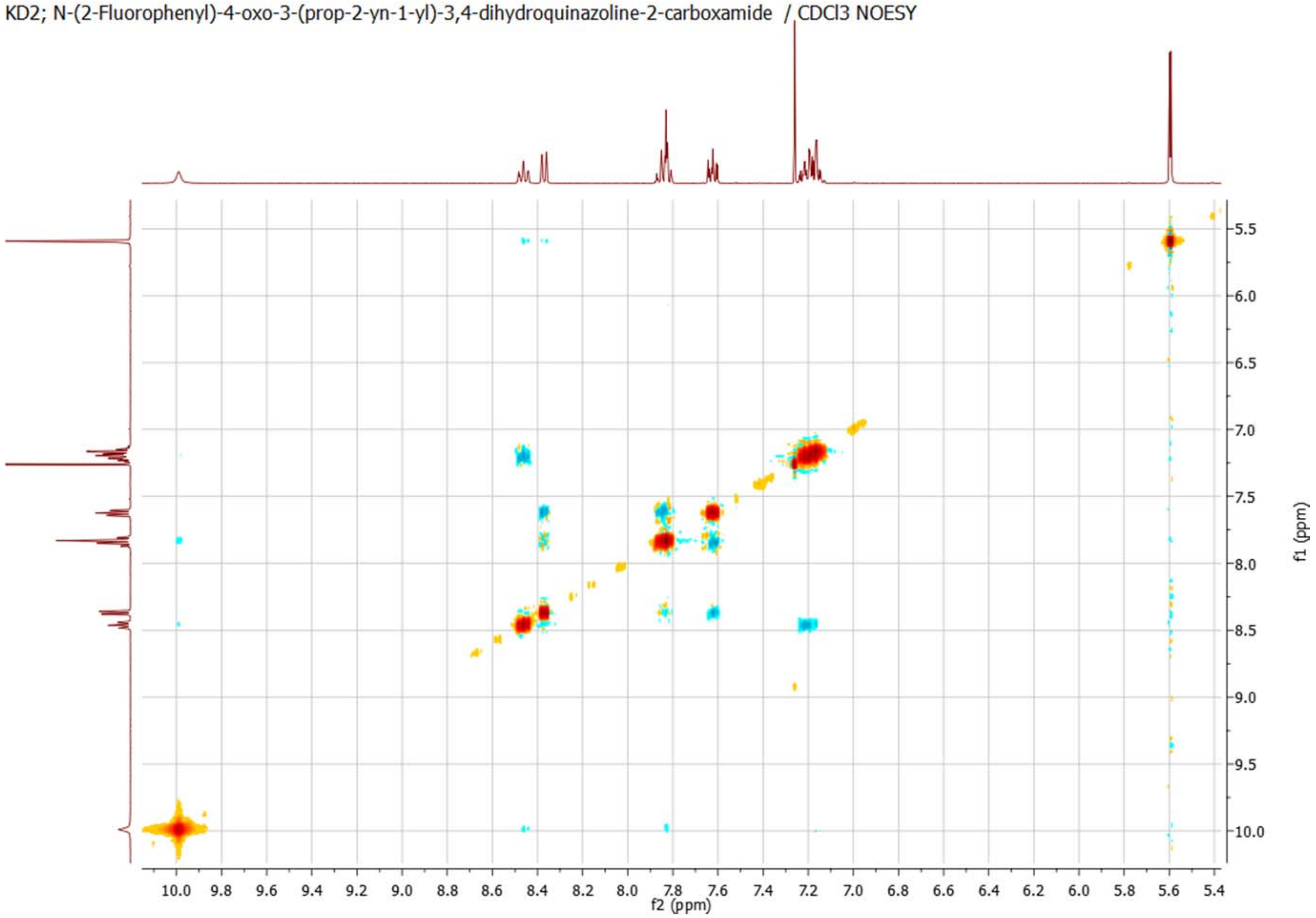

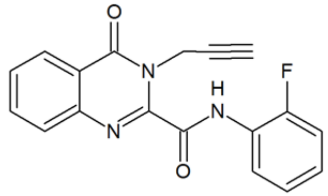

Figure S11. NOESY spectrum of *N*-(2-fluorophenyl)-4-oxo-3-(prop-2-yn-1-yl)-3,4-dihydroquinazoline-2-carboxamide (**3**)

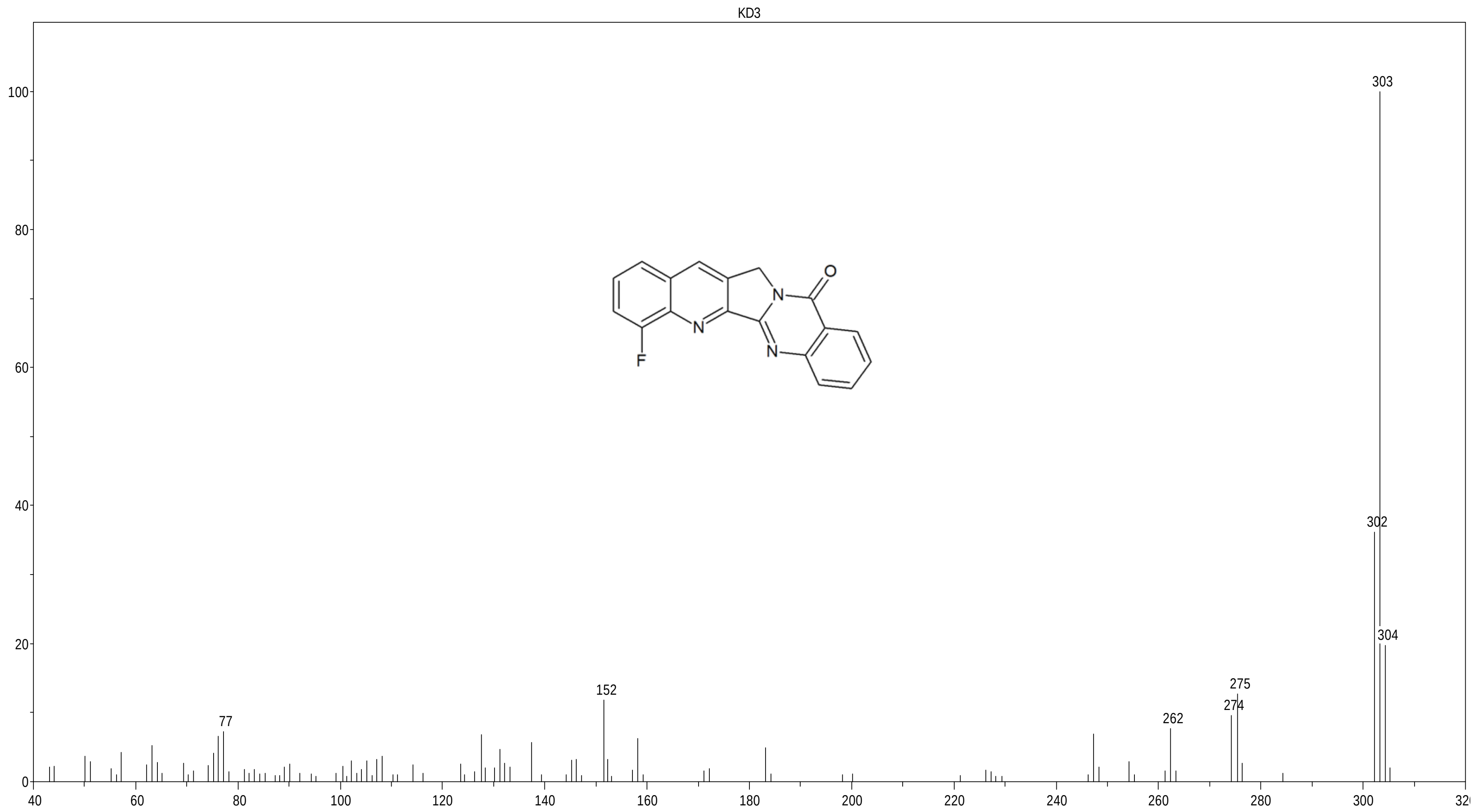

Figure S12. EI-MS of 4-fluoroquinolino[2',3':3,4]pyrrolo[2,1-*b*]quinazolin-11(13*H*)-one (**4**)

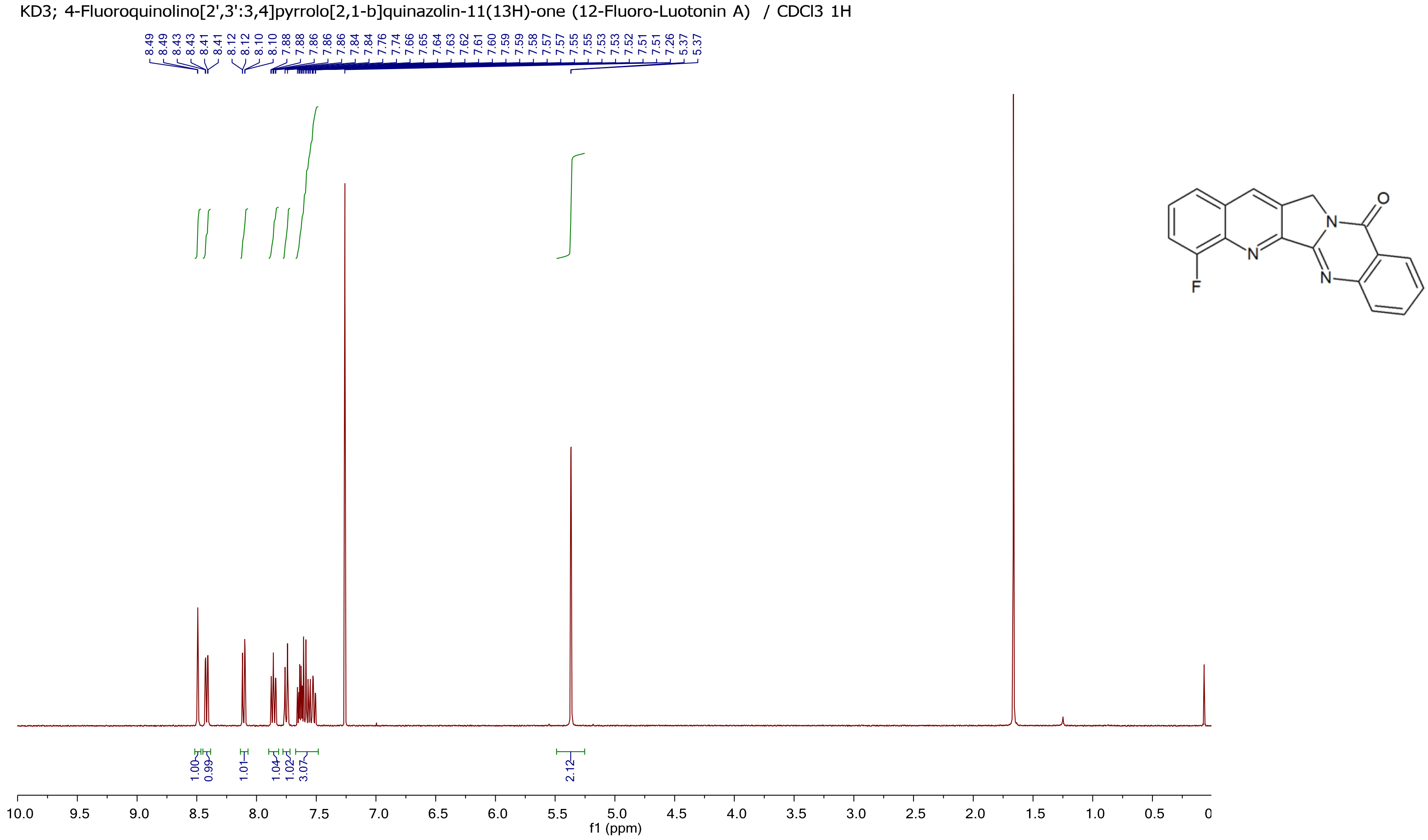

Figure S13. ^{1}H-NMR spectrum of 4-fluoroquinolino[2',3':3,4]pyrrolo[2,1-*b*]quinazolin-11(13*H*)-one (**4**)

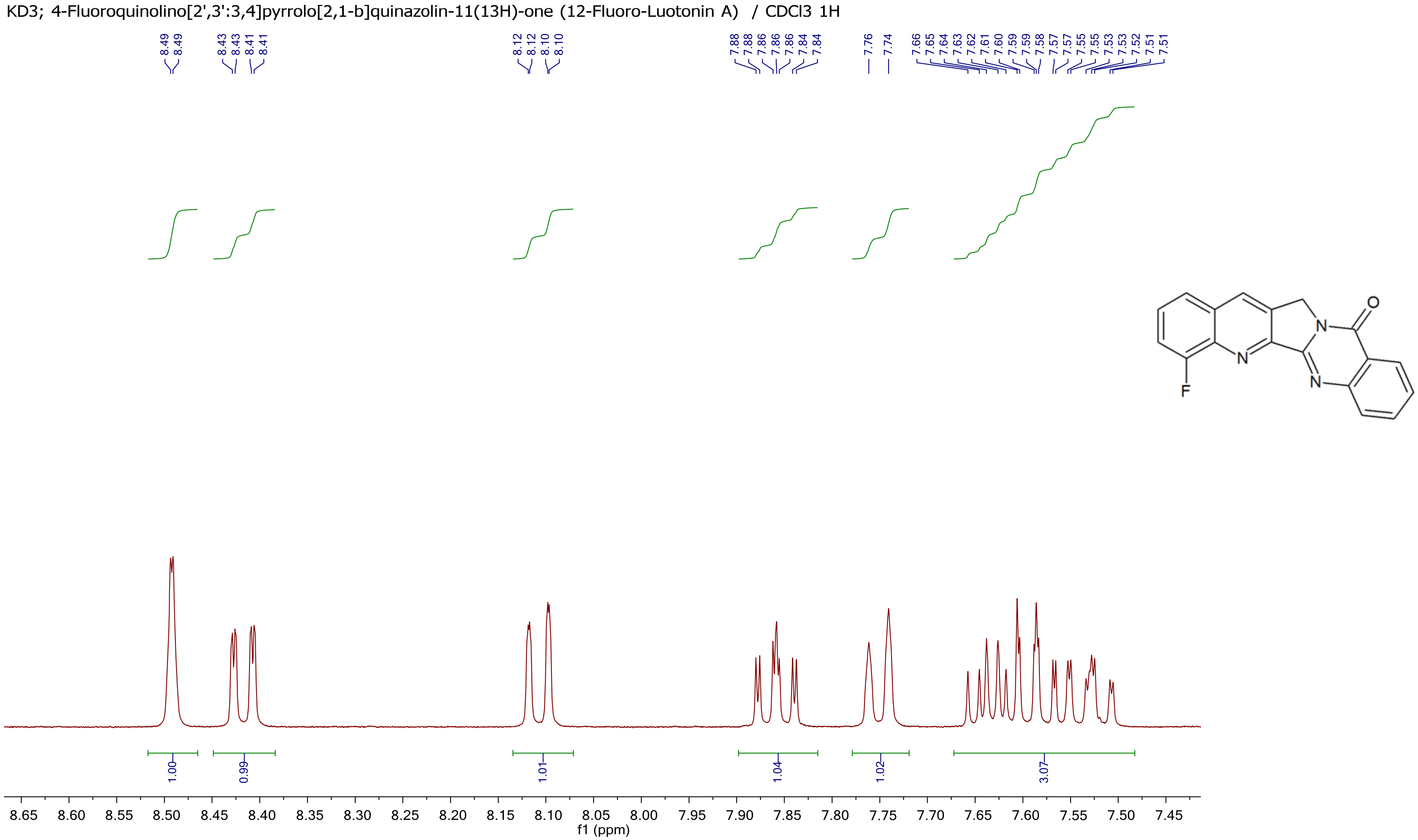

Figure S14. ^{1}H-NMR spectrum of 4-fluoroquinolino[2',3':3,4]pyrrolo[2,1-*b*]quinazolin-11(13*H*)-one (**4**)

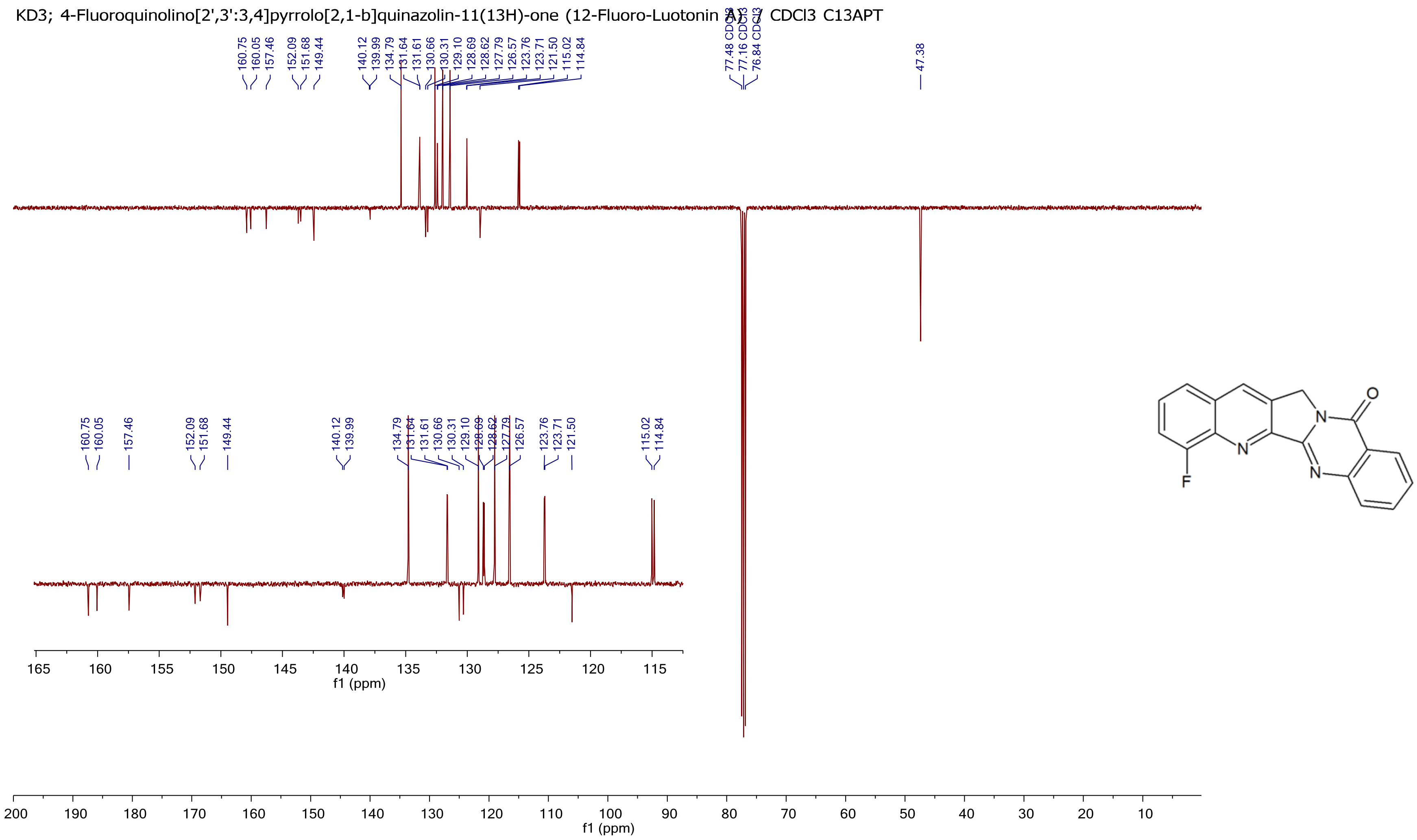

Figure S15. ^{13}C-NMR spectrum of 4-fluoroquinolino[2',3':3,4]pyrrolo[2,1-*b*]quinazolin-11(13*H*)-one (**4**)

KD3; 4-Fluoroquinolino[2',3':3,4]pyrrolo[2,1-b]quinazolin-11(13H)-one (12-Fluoro-Luotonin A) / CDCl3 COSY

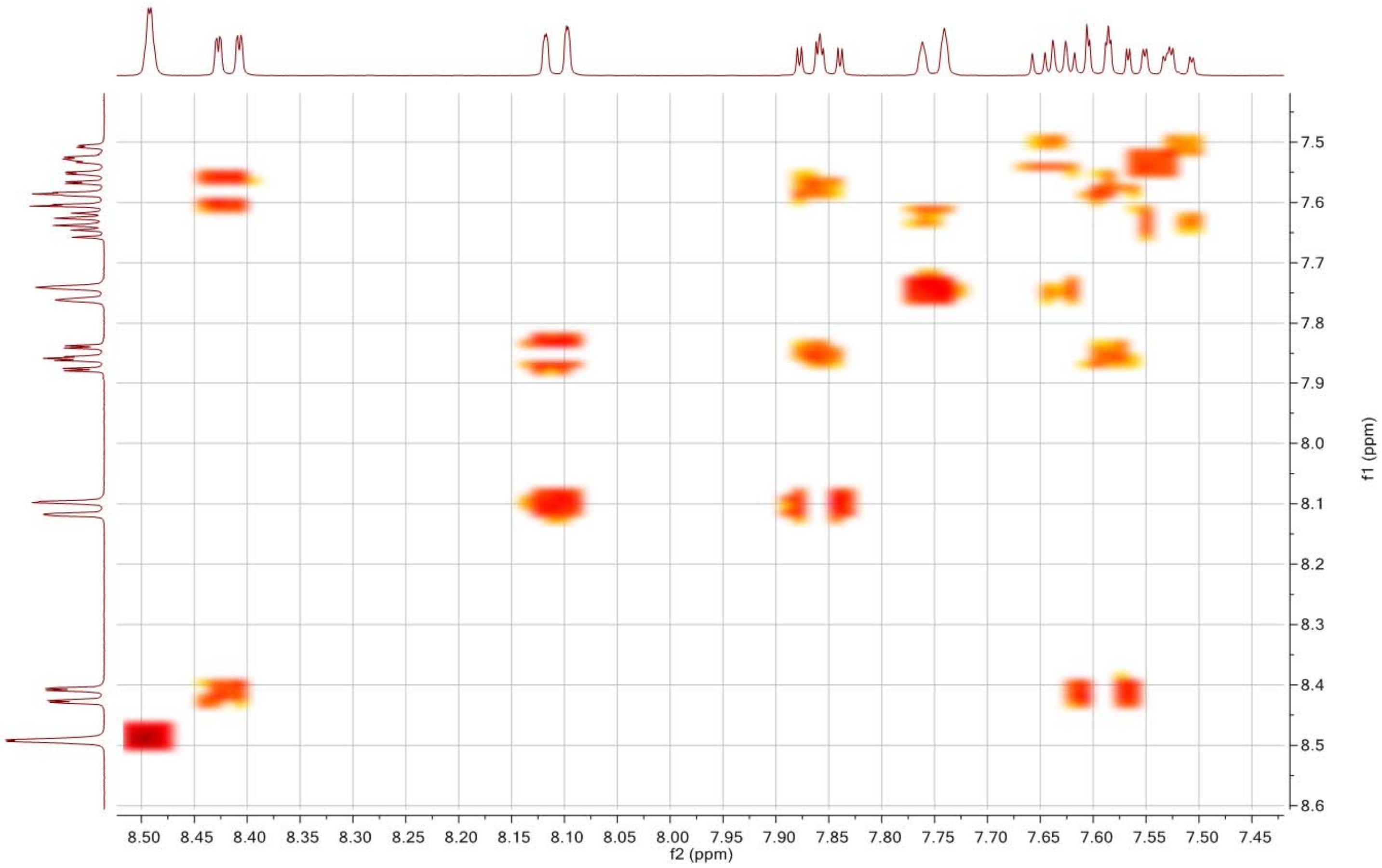

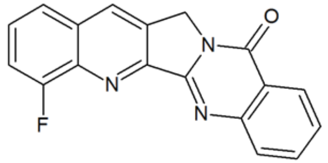

Figure S16. COSY spectrum of 4-fluoroquinolino[2',3':3,4]pyrrolo[2,1-*b*]quinazolin-11(13*H*)-one (**4**)

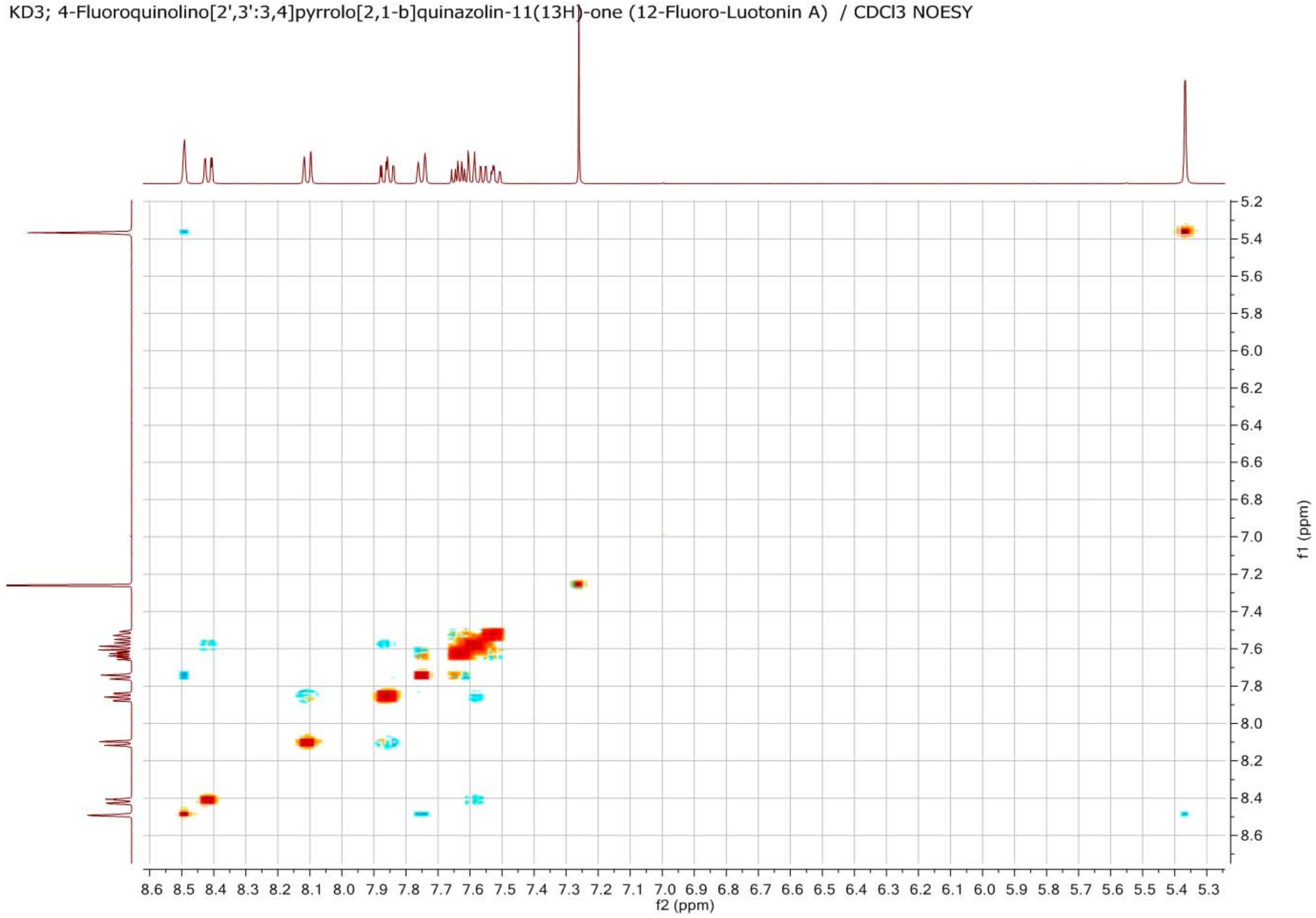

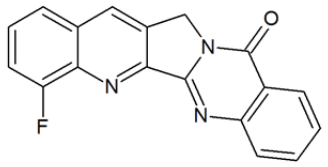

Figure S17. NOESY spectrum of 4-fluoroquinolino[2',3':3,4]pyrrolo[2,1-*b*]quinazolin-11(13*H*)-one (**4**)

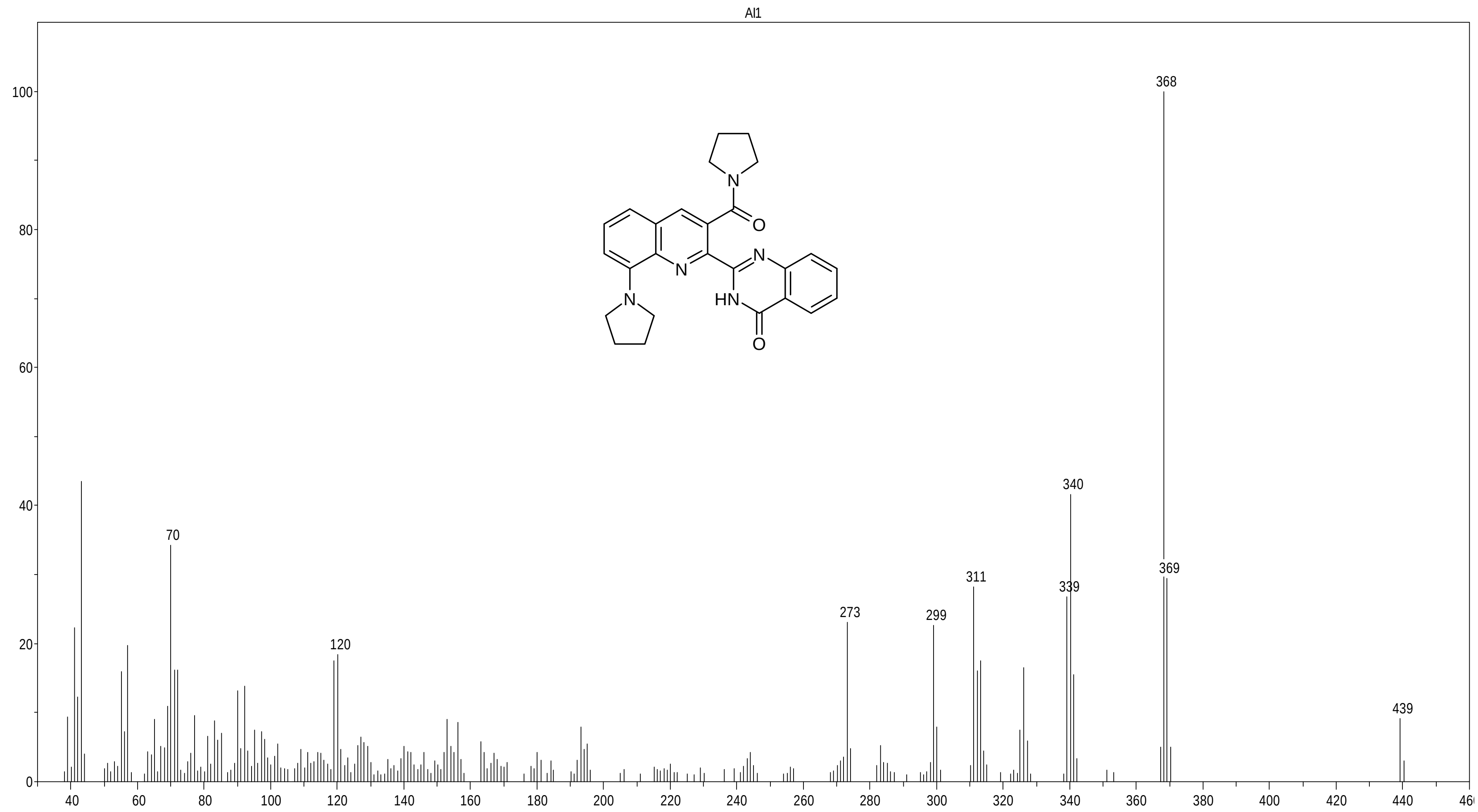

Figure S18. EI-MS of 2-[8-(pyrrolidin-1-yl)-3-(pyrrolidin-1-ylcarbonyl)quinolin-2-yl]quinazolin-4(3*H*)-one (**5**)

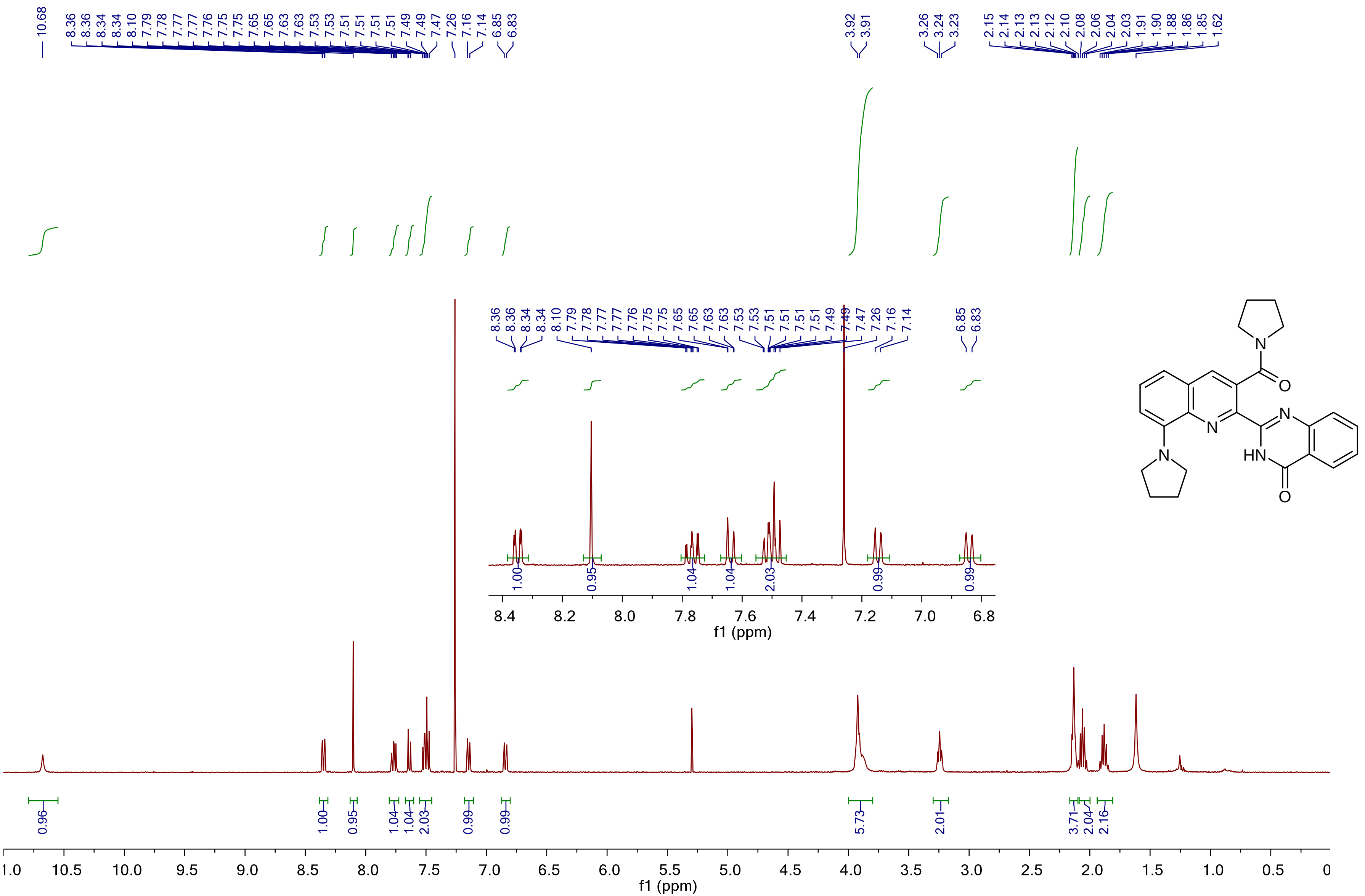

Figure S19. ^{1}H-NMR spectrum of 2-[8-(pyrrolidin-1-yl)-3-(pyrrolidin-1-ylcarbonyl)quinolin-2-yl]quinazolin-4(3*H*)-one (**5**)

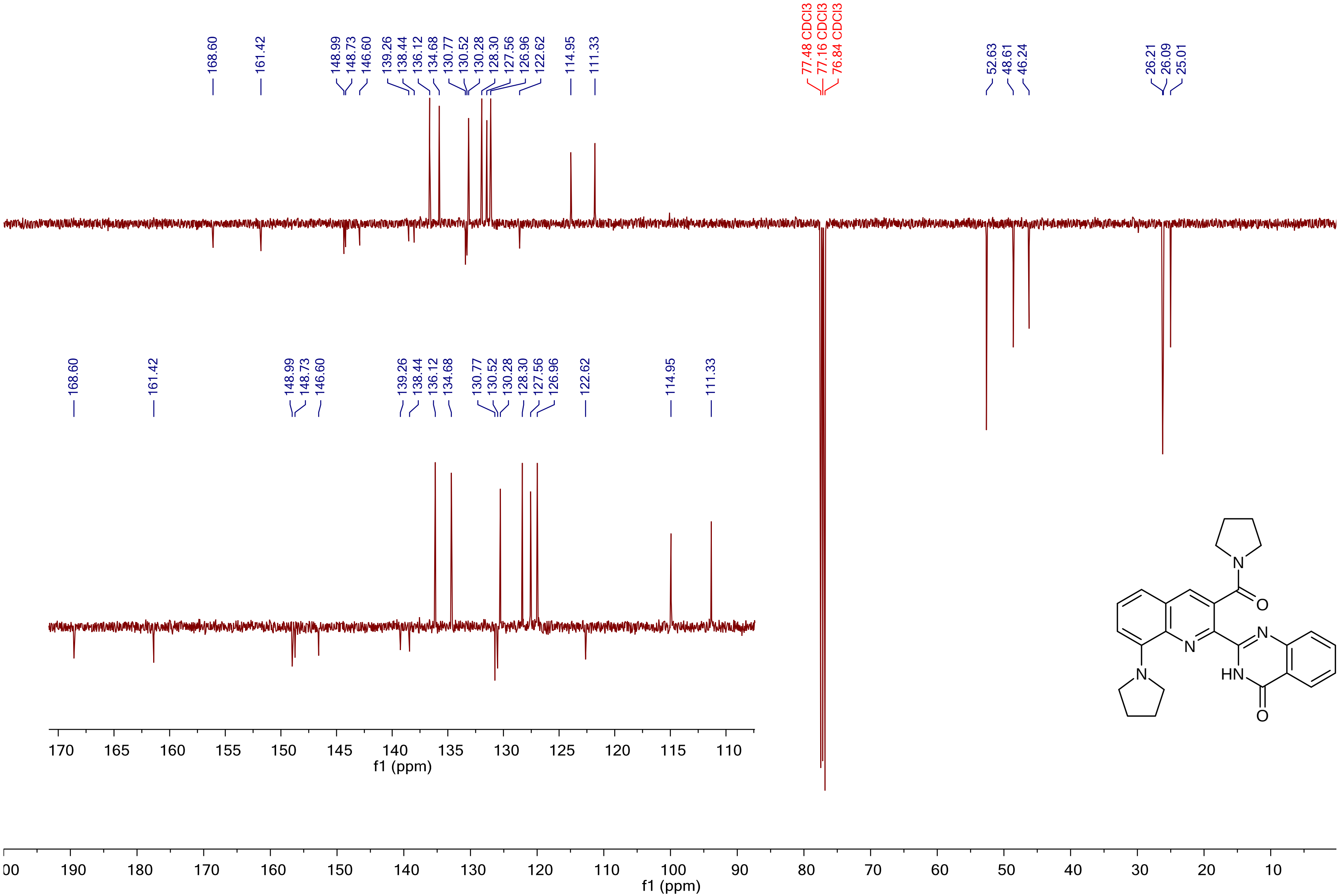

Figure S20. ^{1}H-NMR spectrum of 2-[8-(pyrrolidin-1-yl)-3-(pyrrolidin-1-ylcarbonyl)quinolin-2-yl]quinazolin-4(3*H*)-one (**5**)

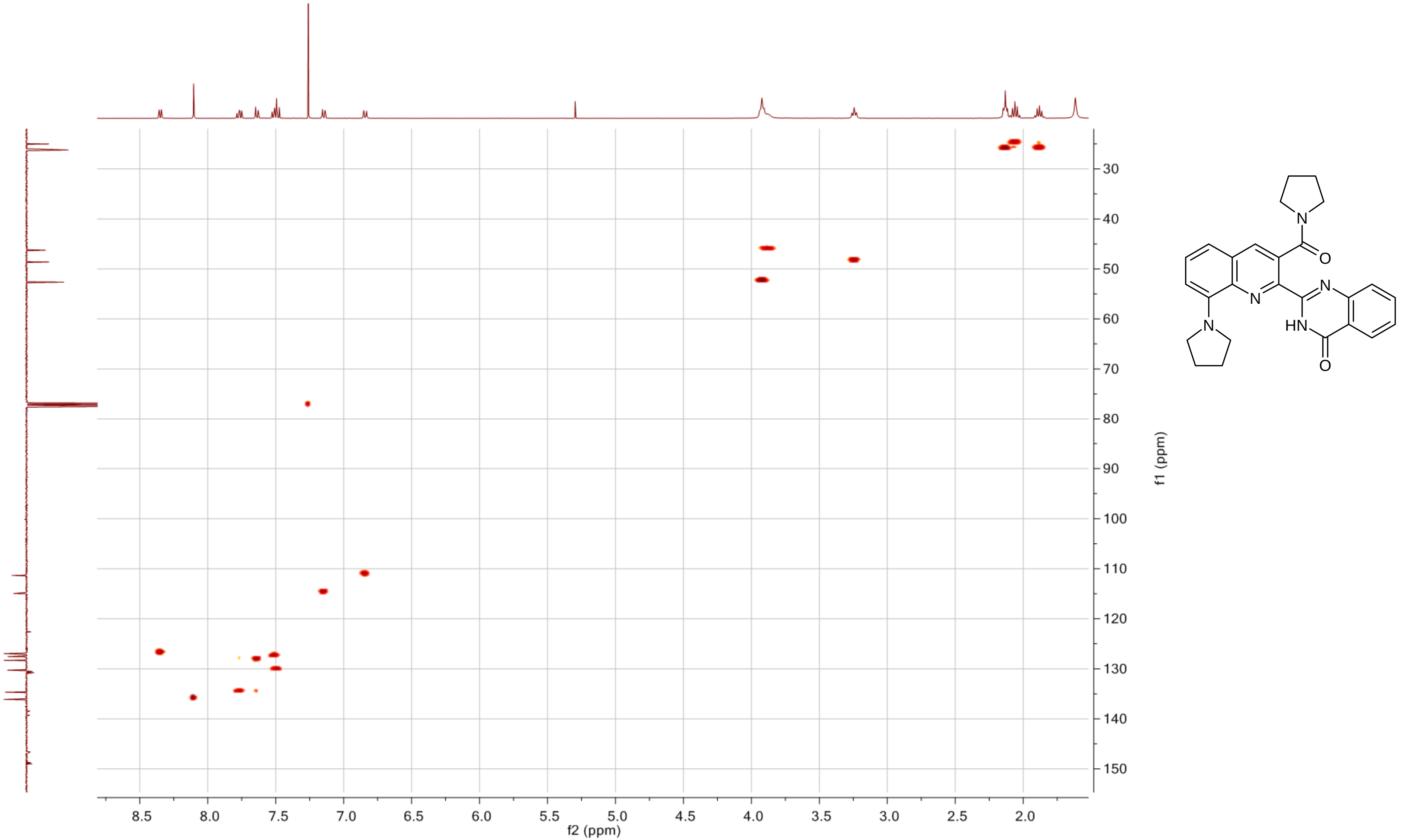

Figure S21. HSQC spectrum of 2-[8-(pyrrolidin-1-yl)-3-(pyrrolidin-1-ylcarbonyl)quinolin-2-yl]quinazolin-4(3*H*)-one (**5**)

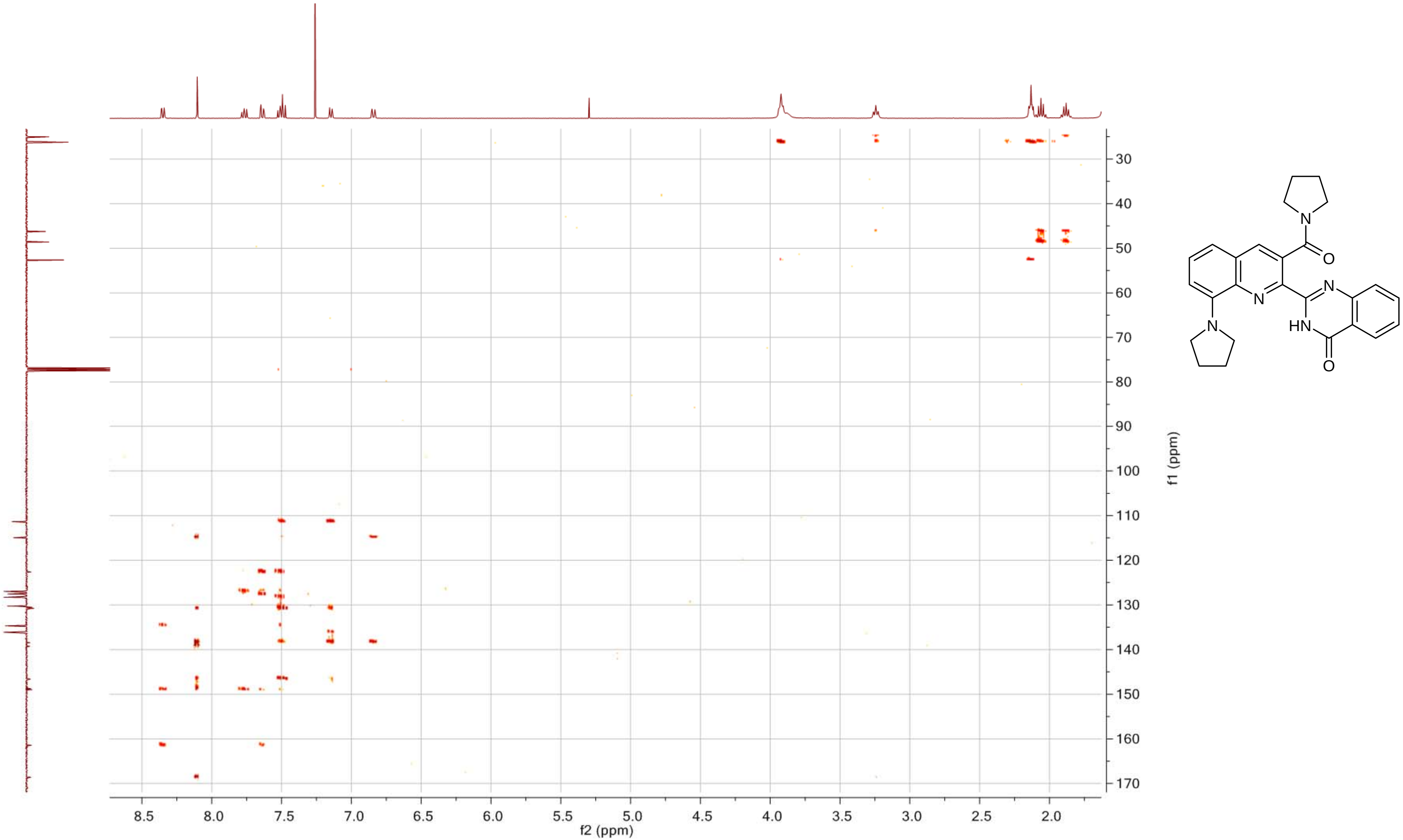

Figure S22. HMBC spectrum of 2-[8-(pyrrolidin-1-yl)-3-(pyrrolidin-1-ylcarbonyl)quinolin-2-yl]quinazolin-4(3*H*)-one (**5**)

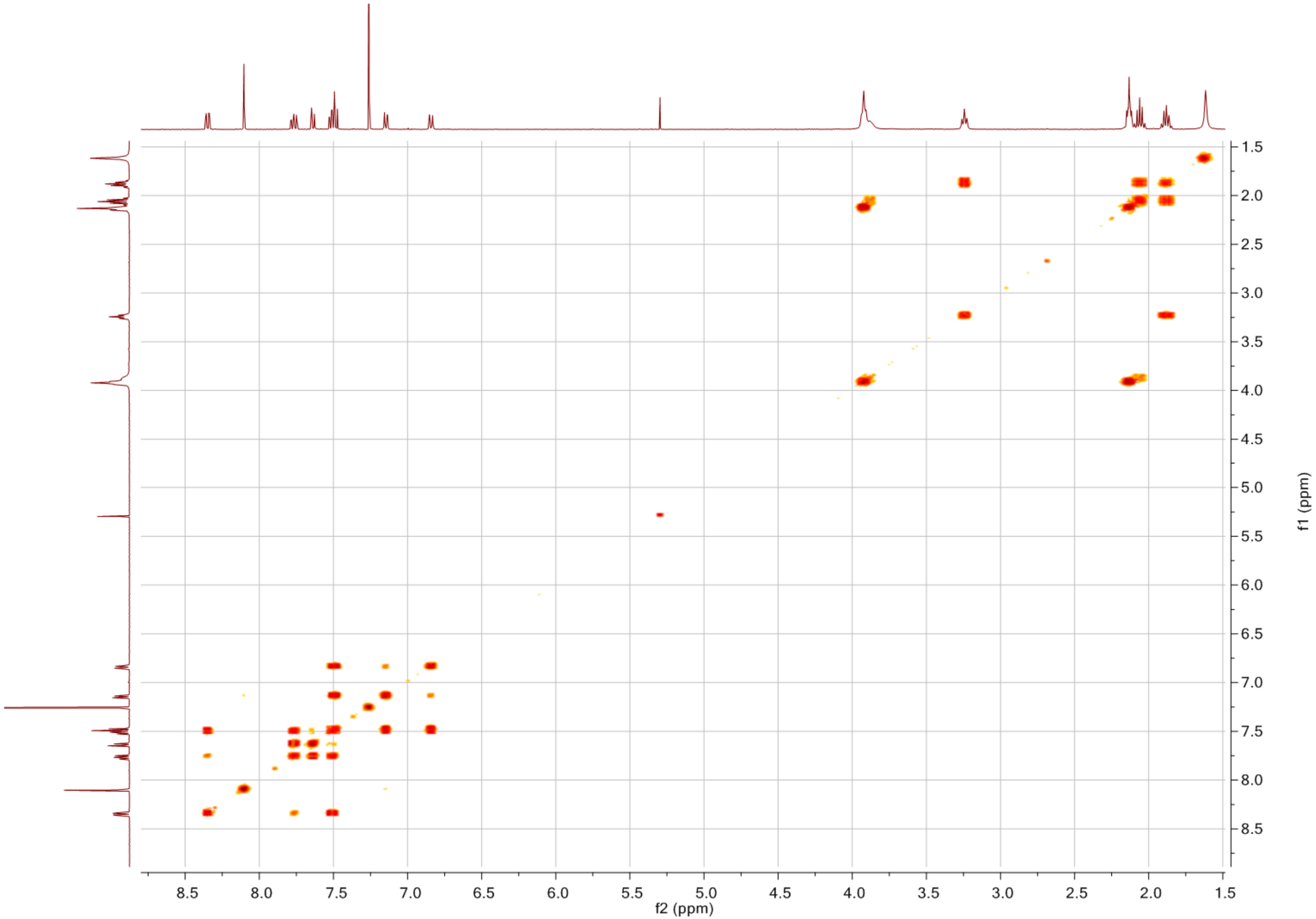

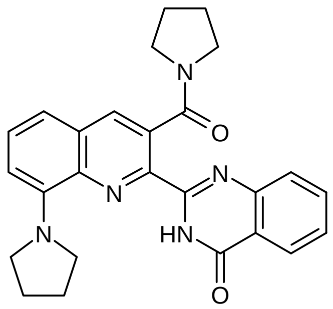

Figure S23. COSY spectrum of 2-[8-(pyrrolidin-1-yl)-3-(pyrrolidin-1-ylcarbonyl)quinolin-2-yl]quinazolin-4(3*H*)-one (**5**)

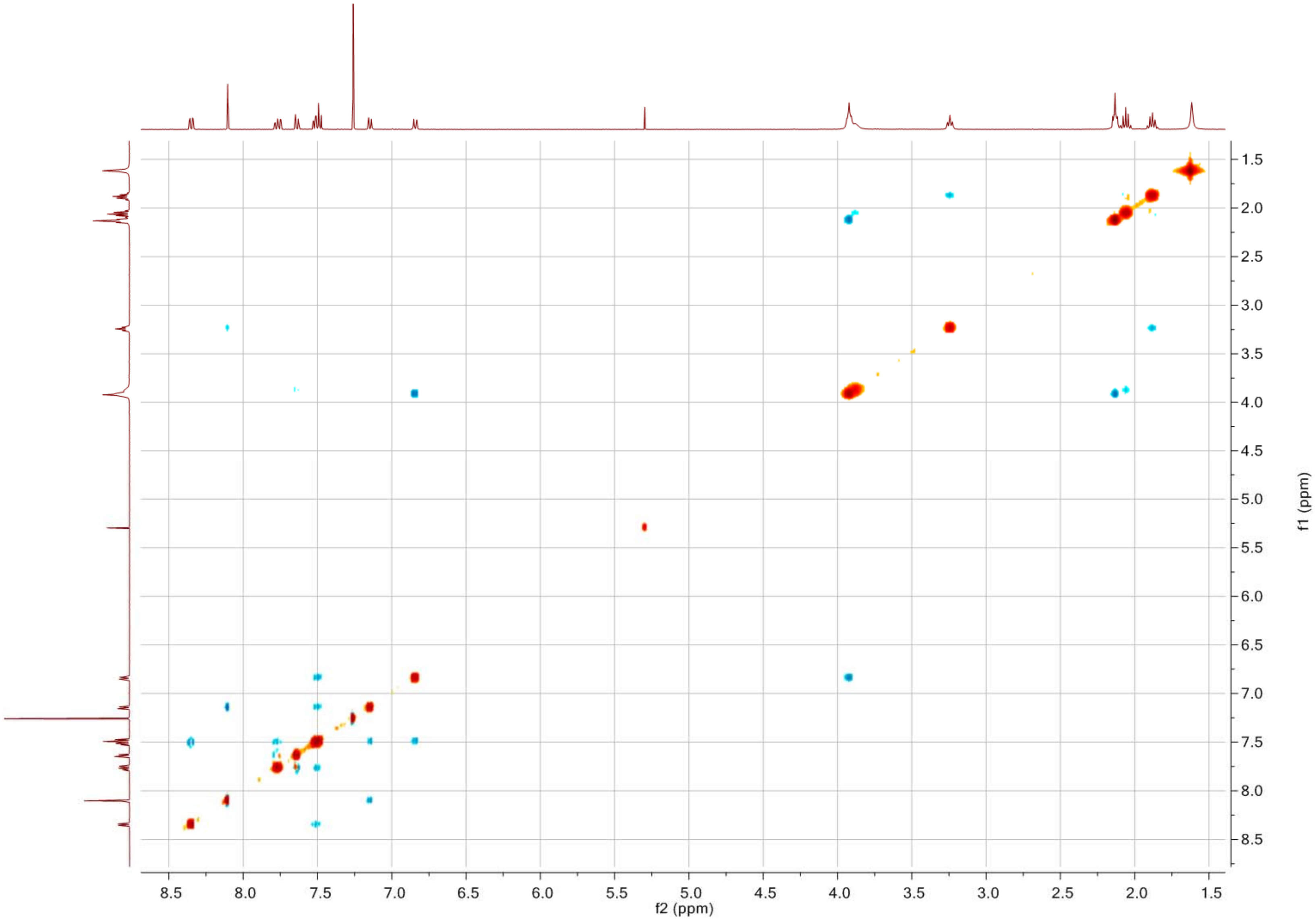

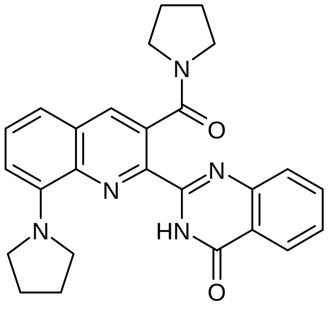

Figure S24. NOESY spectrum of 2-[8-(pyrrolidin-1-yl)-3-(pyrrolidin-1-ylcarbonyl)quinolin-2-yl]quinazolin-4(3*H*)-one (**5**)

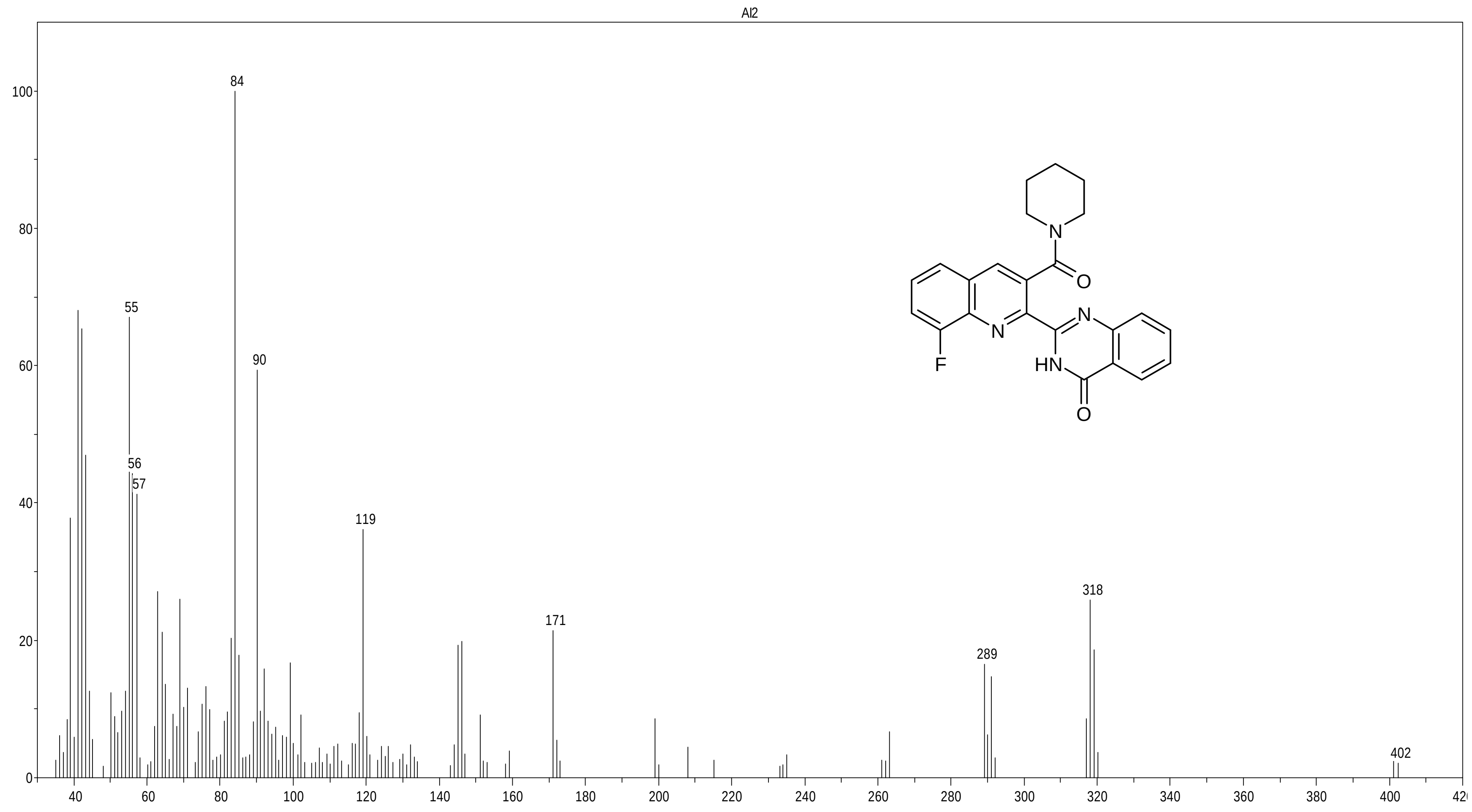

Figure S25. EI-MS of 2-[8-fluoro-3-(piperidin-1-ylcarbonyl)quinolin-2-yl]quinazolin-4(3*H*)-one (**6**)

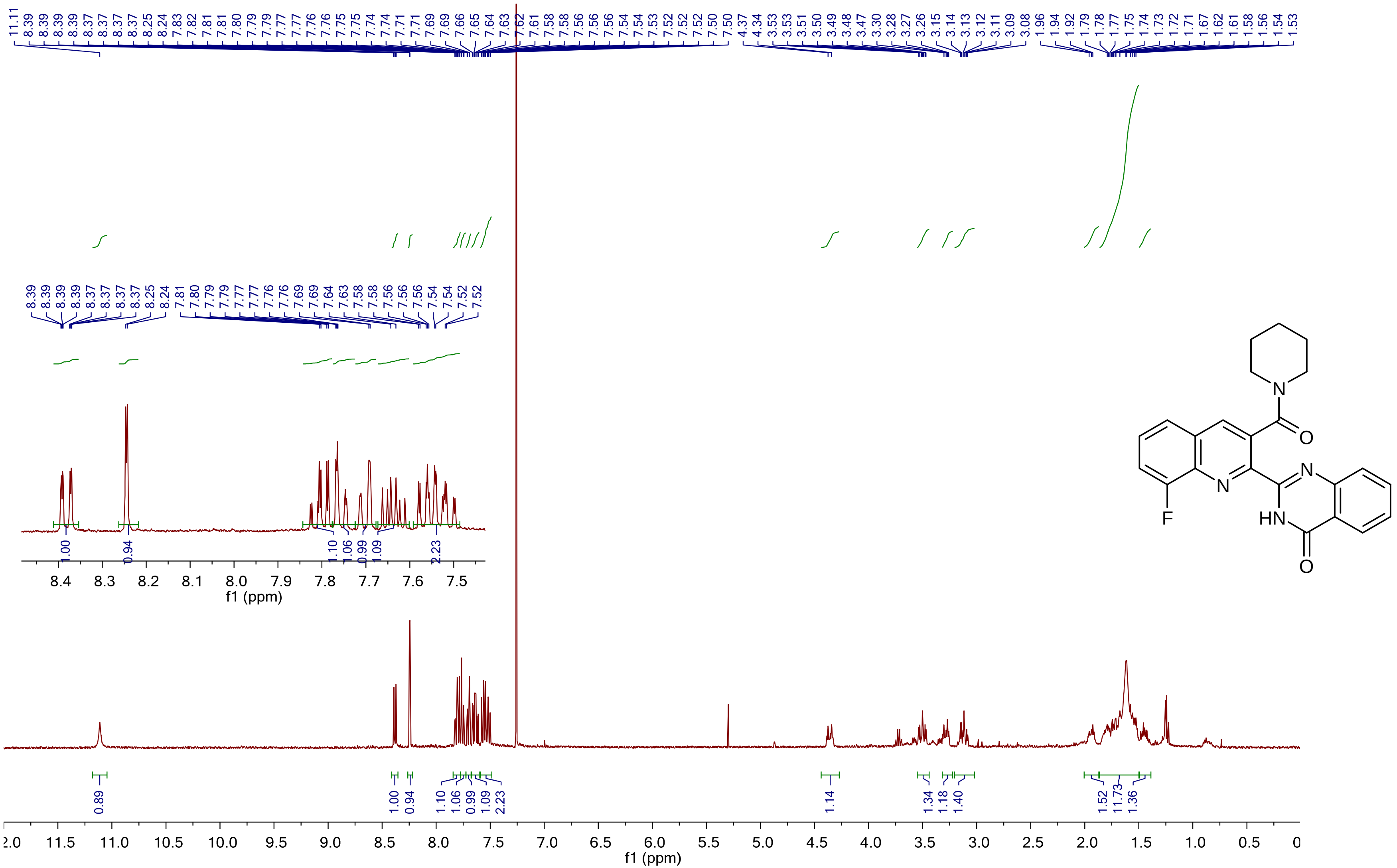

Figure S26. ^{1}H-NMR spectrum of 2-[8-fluoro-3-(piperidin-1-ylcarbonyl)quinolin-2-yl]quinazolin-4(3*H*)-one (**6**)

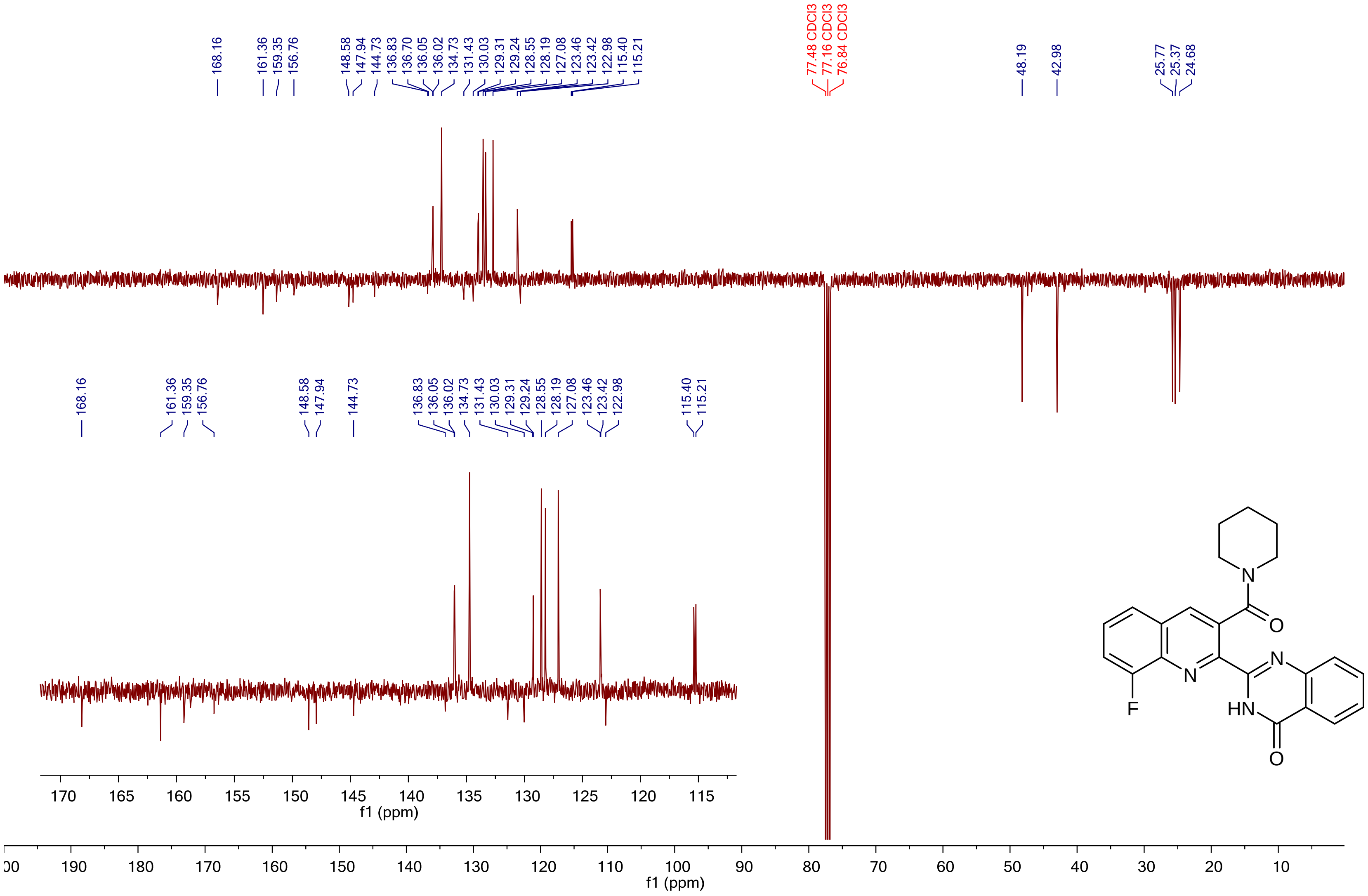

Figure S27. ^{13}C-NMR spectrum of 2-[8-fluoro-3-(piperidin-1-ylcarbonyl)quinolin-2-yl]quinazolin-4(3*H*)-one (**6**)

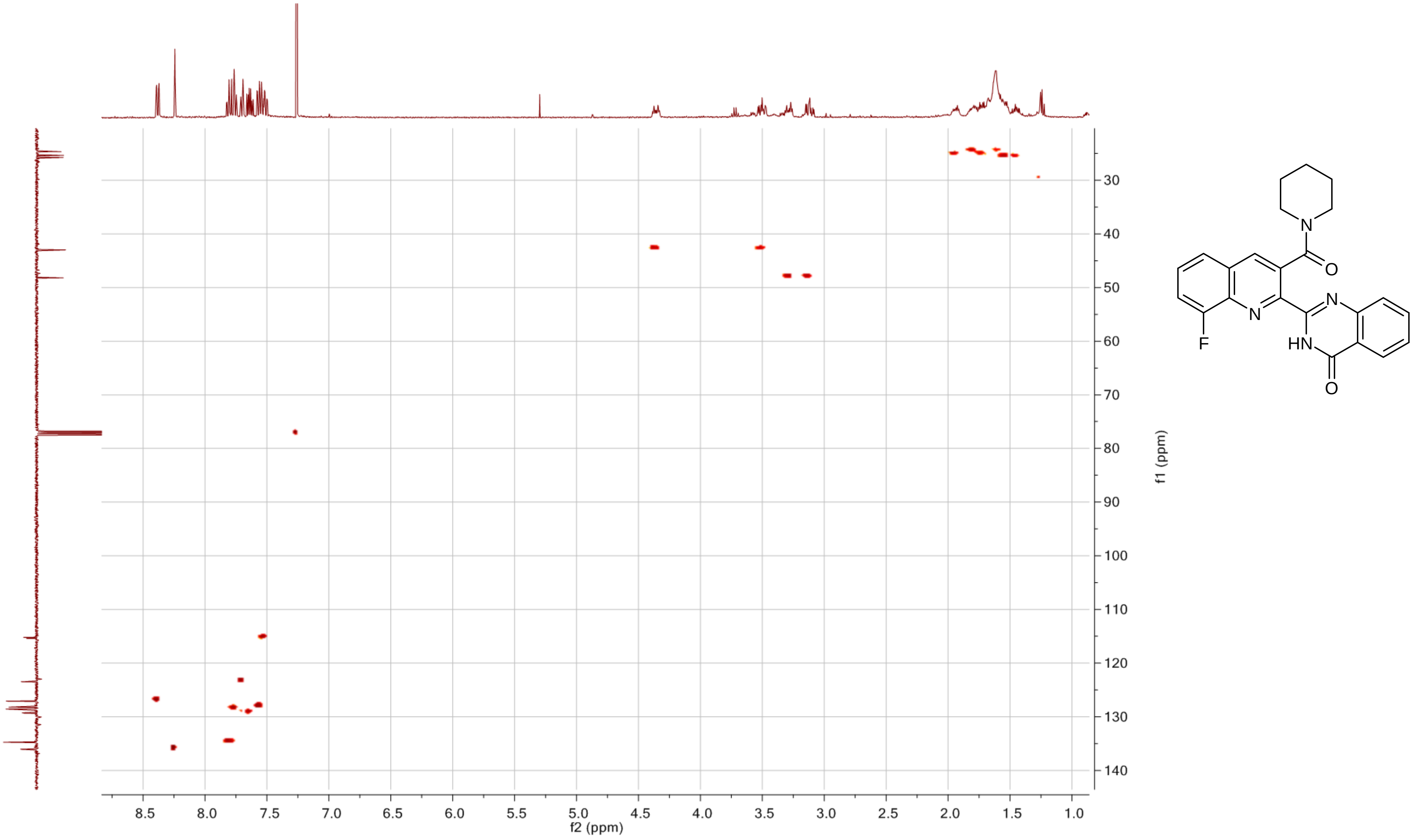

Figure S28. HSQC spectrum of 2-[8-fluoro-3-(piperidin-1-ylcarbonyl)quinolin-2-yl]quinazolin-4(3*H*)-one (**6**)

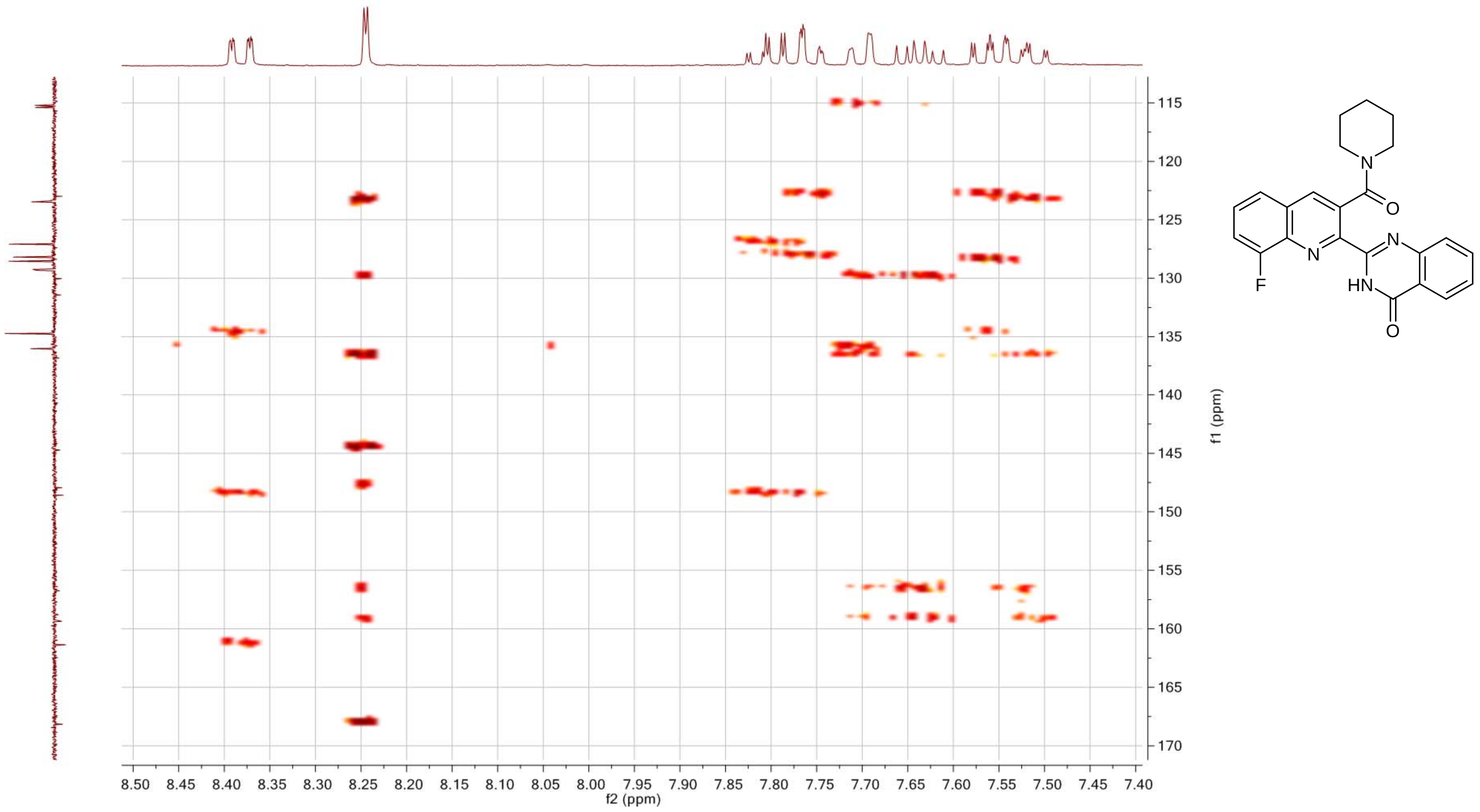

Figure S29. HMBC spectrum of 2-[8-fluoro-3-(piperidin-1-ylcarbonyl)quinolin-2-yl]quinazolin-4(3*H*)-one (**6**)

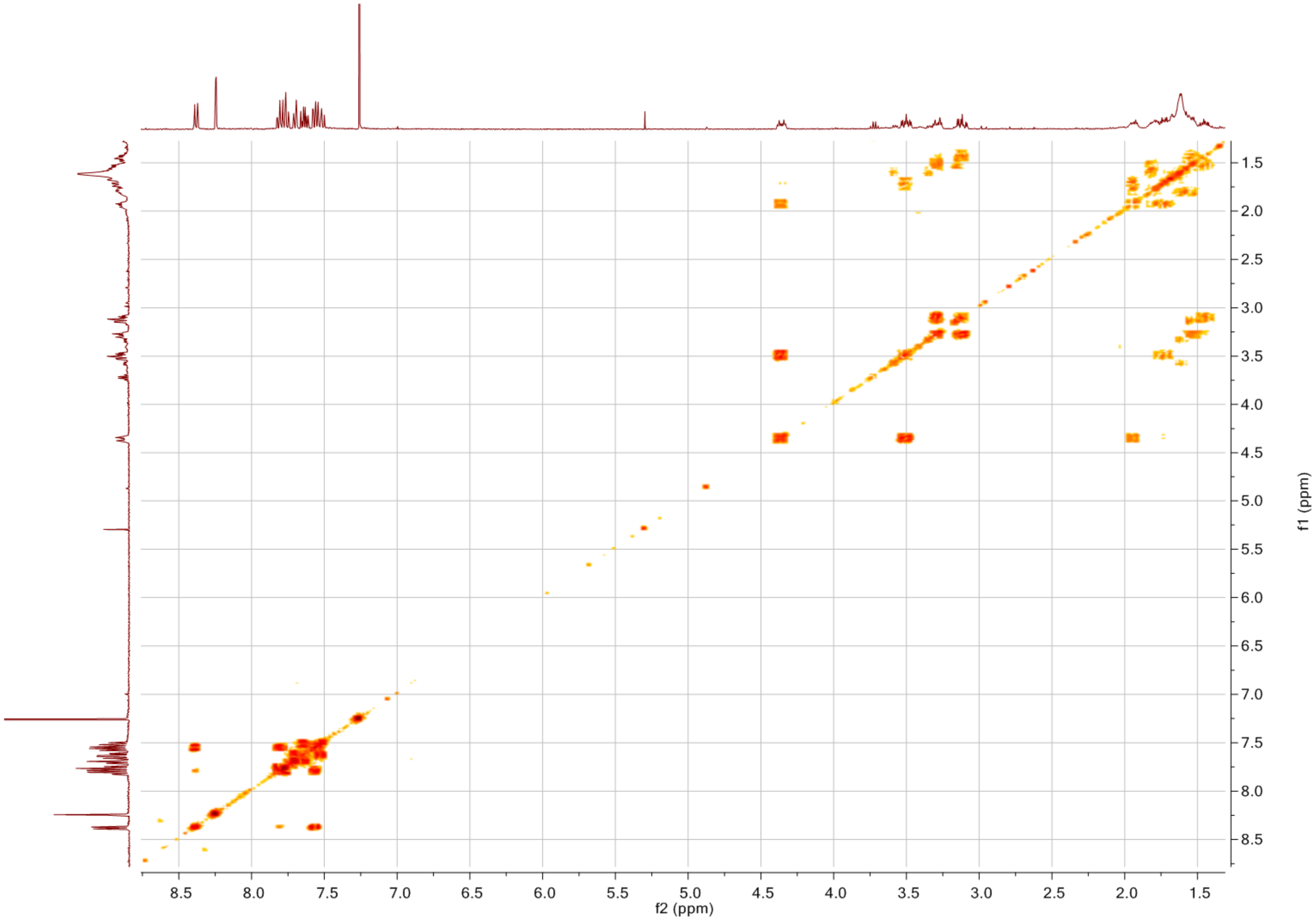

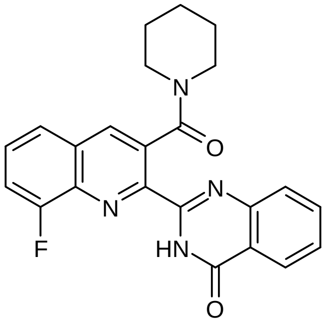

Figure S30. COSY spectrum of 2-[8-fluoro-3-(piperidin-1-ylcarbonyl)quinolin-2-yl]quinazolin-4(3*H*)-one (**6**)

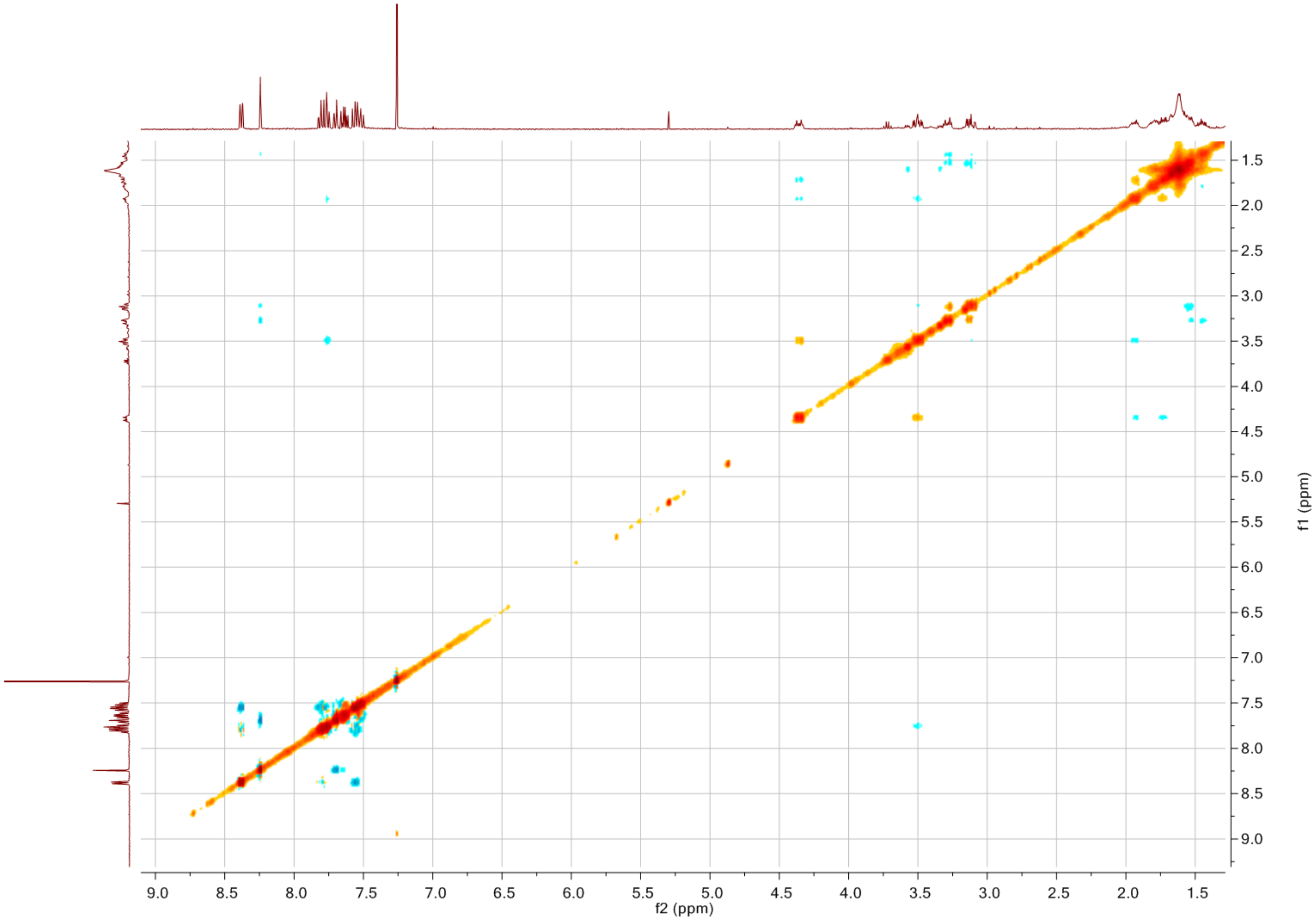

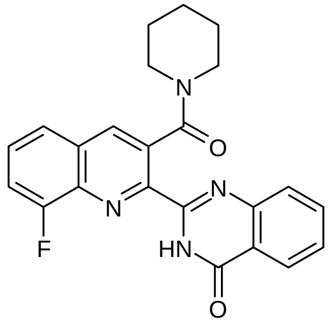

Figure S31. NOESY spectrum of 2-[8-fluoro-3-(piperidin-1-ylcarbonyl)quinolin-2-yl]quinazolin-4(3*H*)-one (**6**)

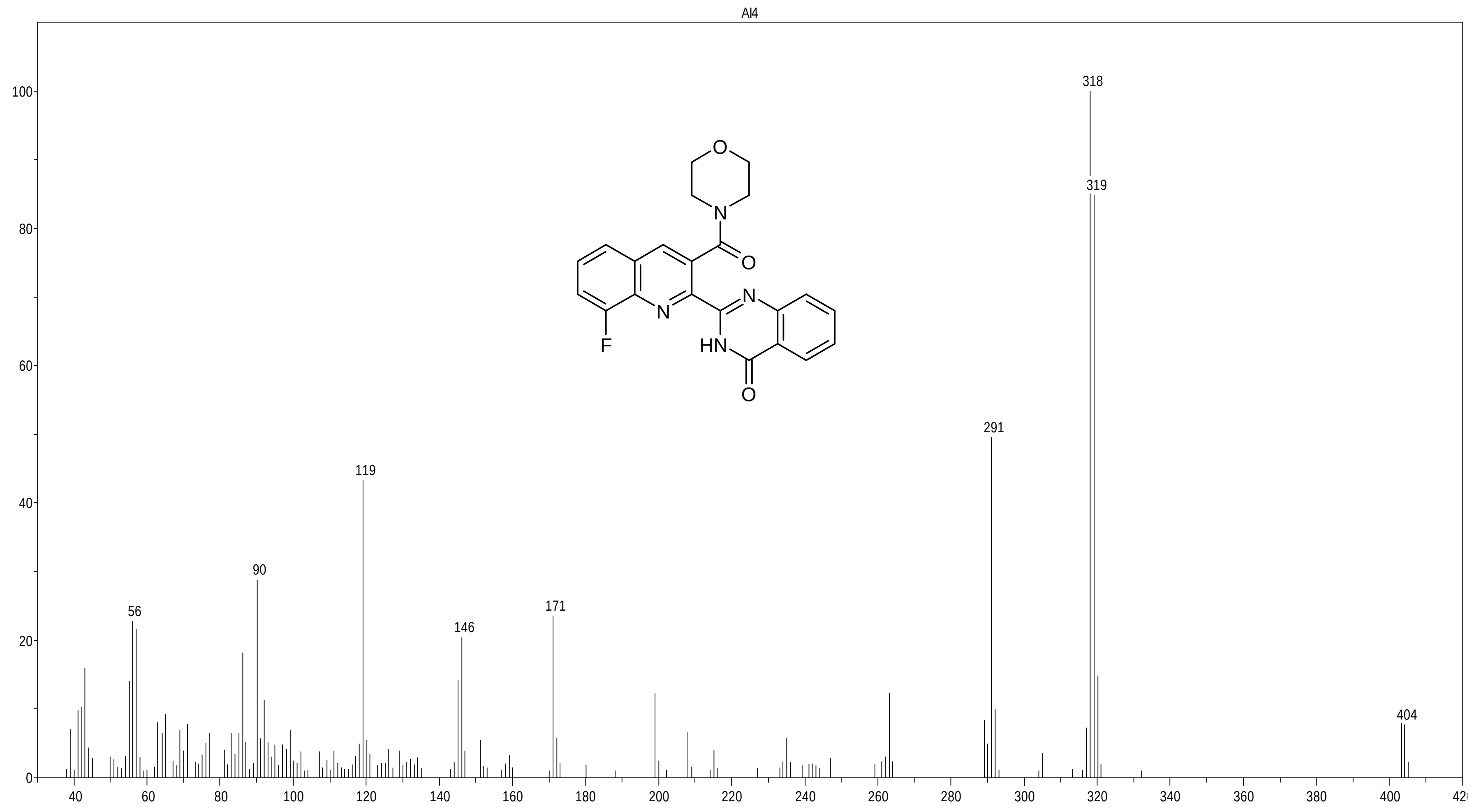

Figure S32. EI-MS of 2-[8-fluoro-3-(morpholin-4-ylcarbonyl)quinolin-2-yl]quinazolin-4(3*H*)-one (**7**)

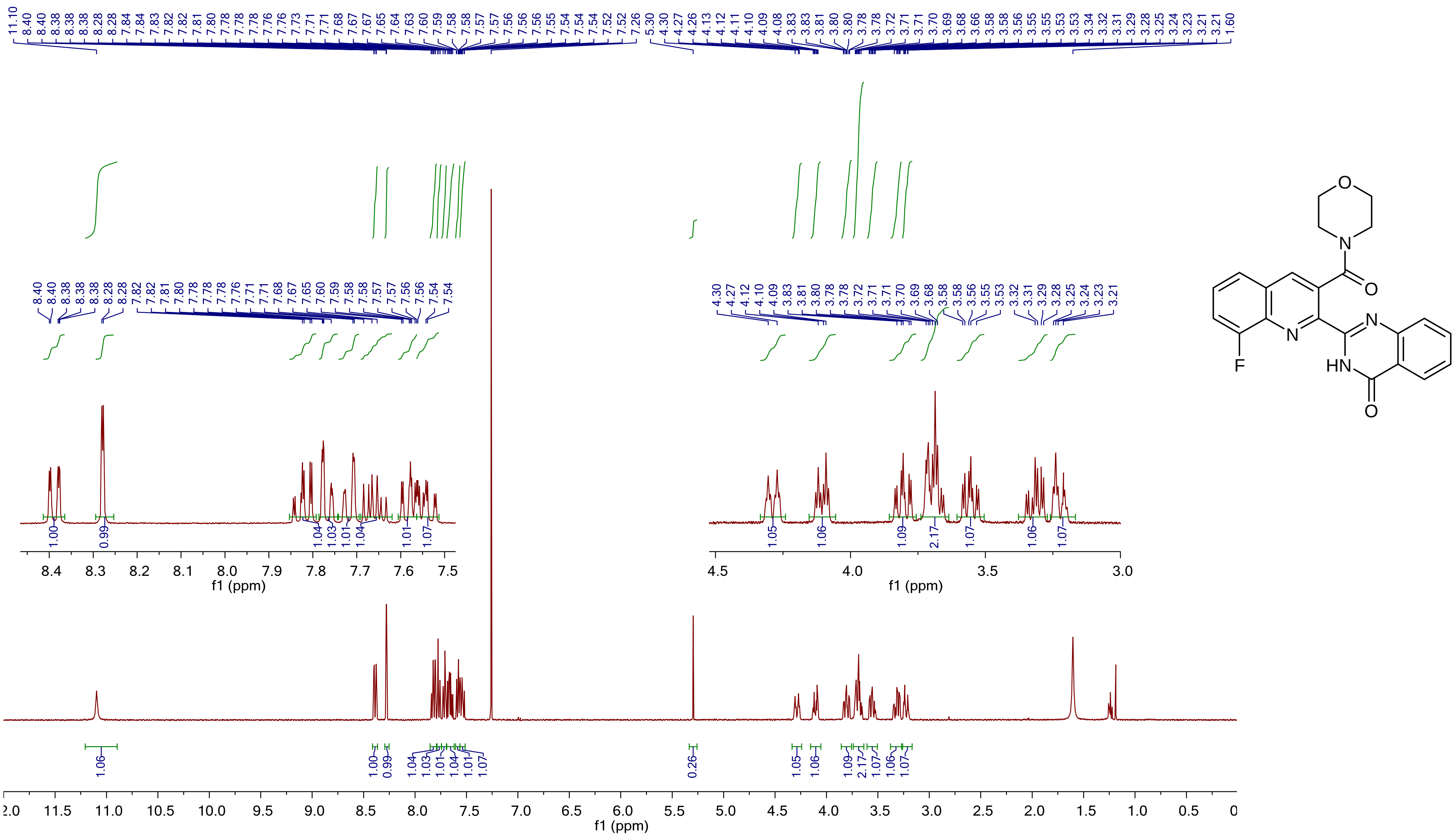

Figure S33. ^{1}H-NMR spectrum of 2-[8-fluoro-3-(morpholin-4-ylcarbonyl)quinolin-2-yl]quinazolin-4(3*H*)-one (**7**)

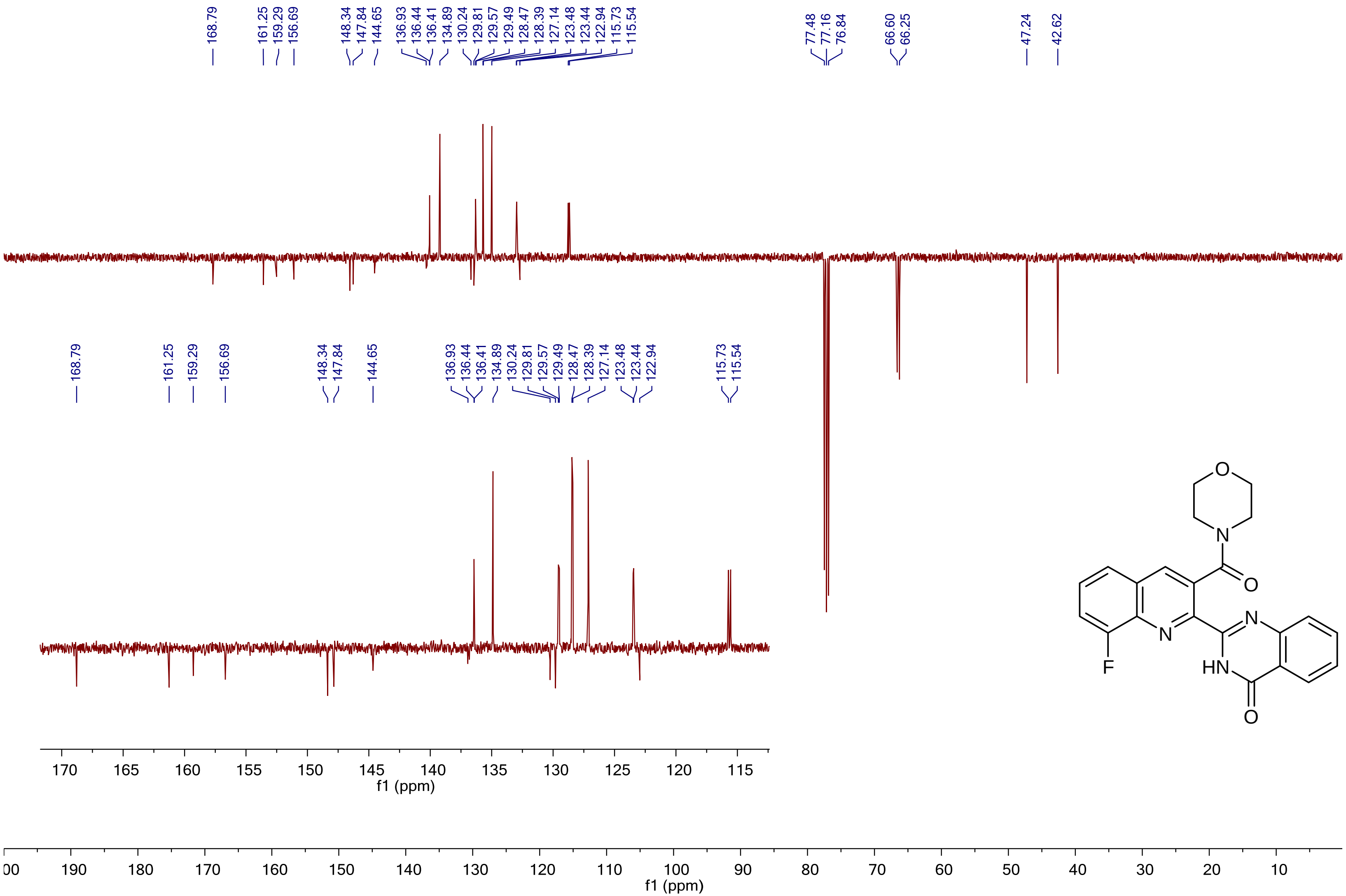

Figure S34. ^{13}C-NMR spectrum of 2-[8-fluoro-3-(morpholin-4-ylcarbonyl)quinolin-2-yl]quinazolin-4(3*H*)-one (**7**)

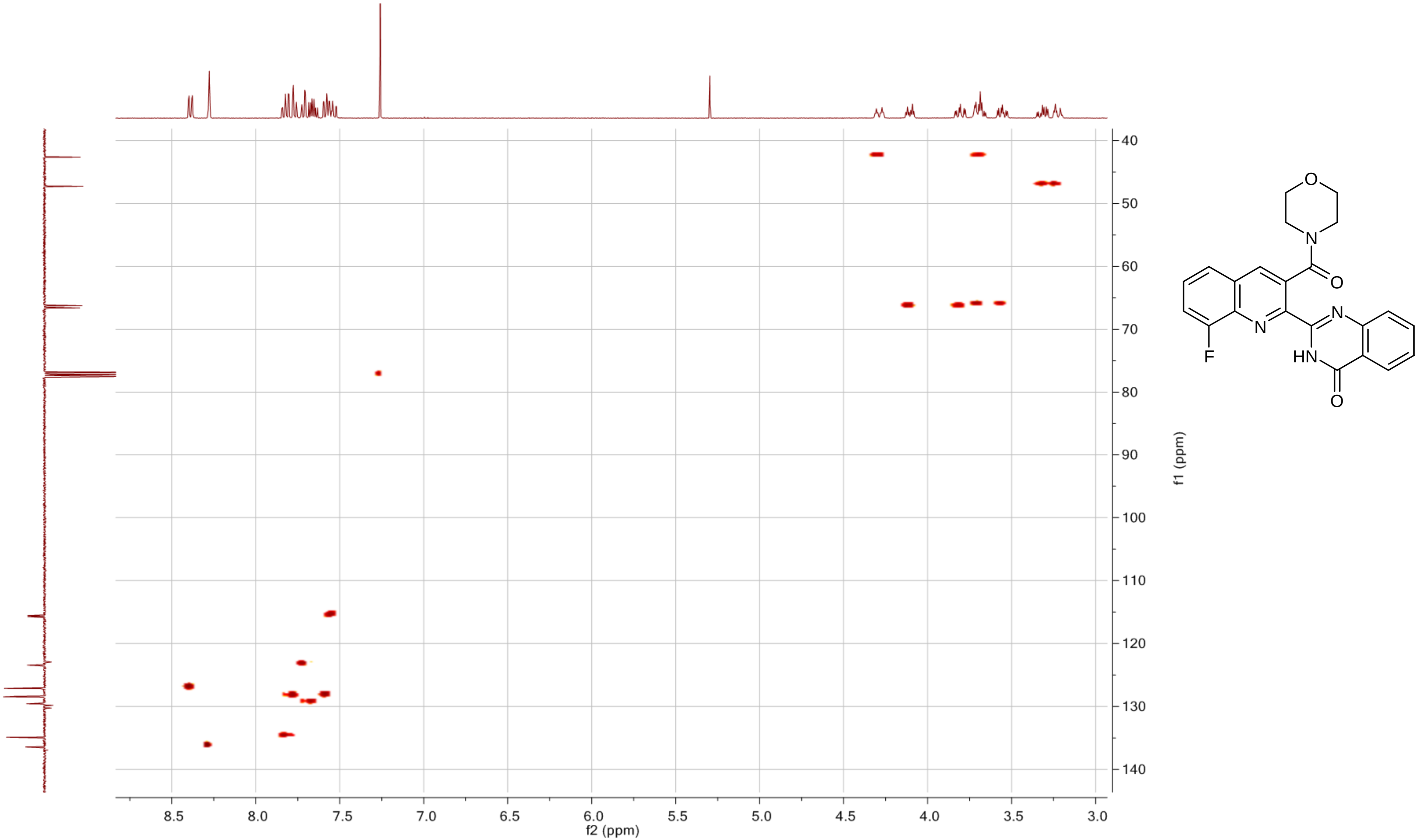

Figure S35. HSQC spectrum of 2-[8-fluoro-3-(morpholin-4-ylcarbonyl)quinolin-2-yl]quinazolin-4(3*H*)-one (**7**)

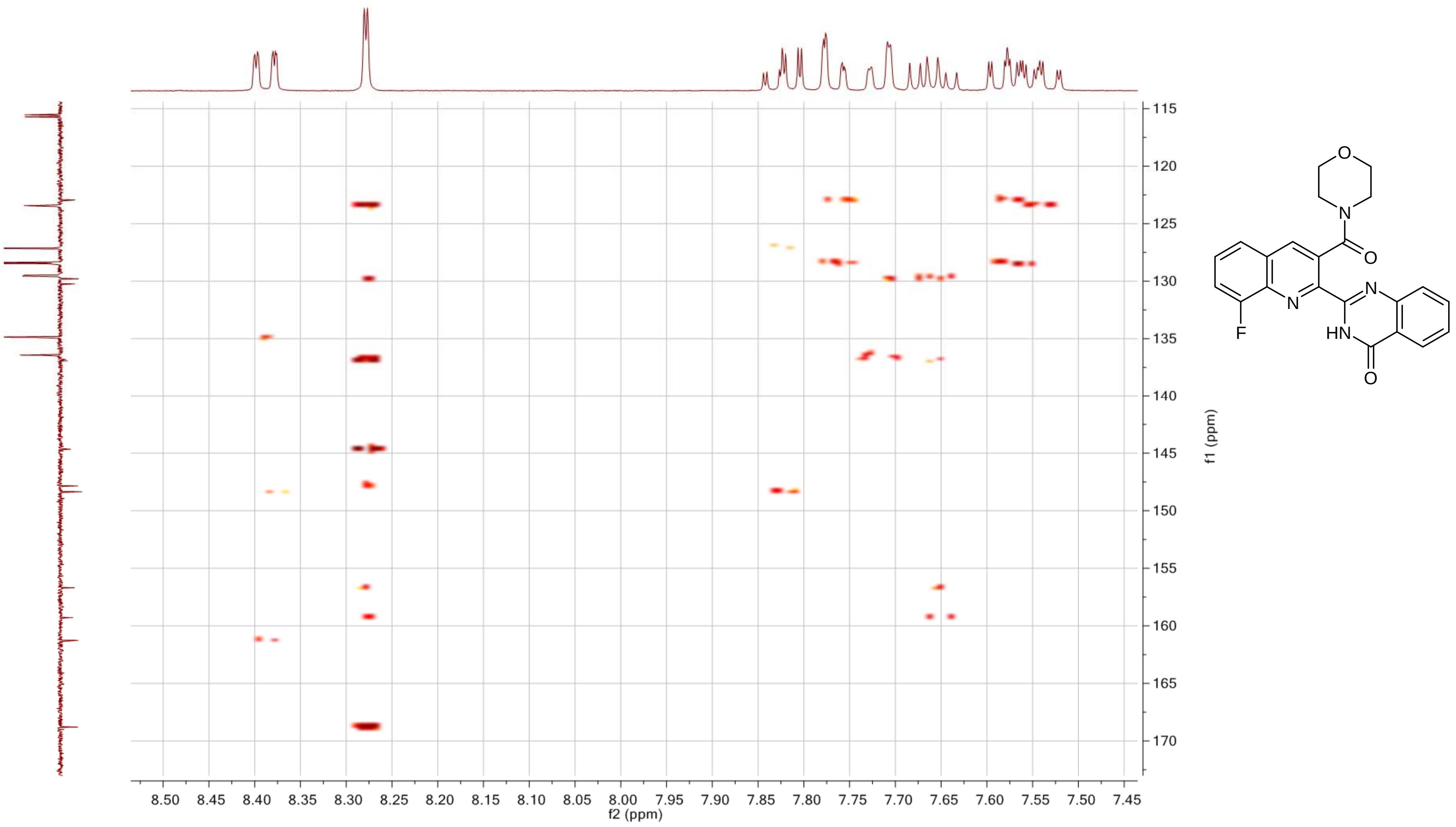

Figure S36. HMBC spectrum of 2-[8-fluoro-3-(morpholin-4-ylcarbonyl)quinolin-2-yl]quinazolin-4(3*H*)-one (**7**)

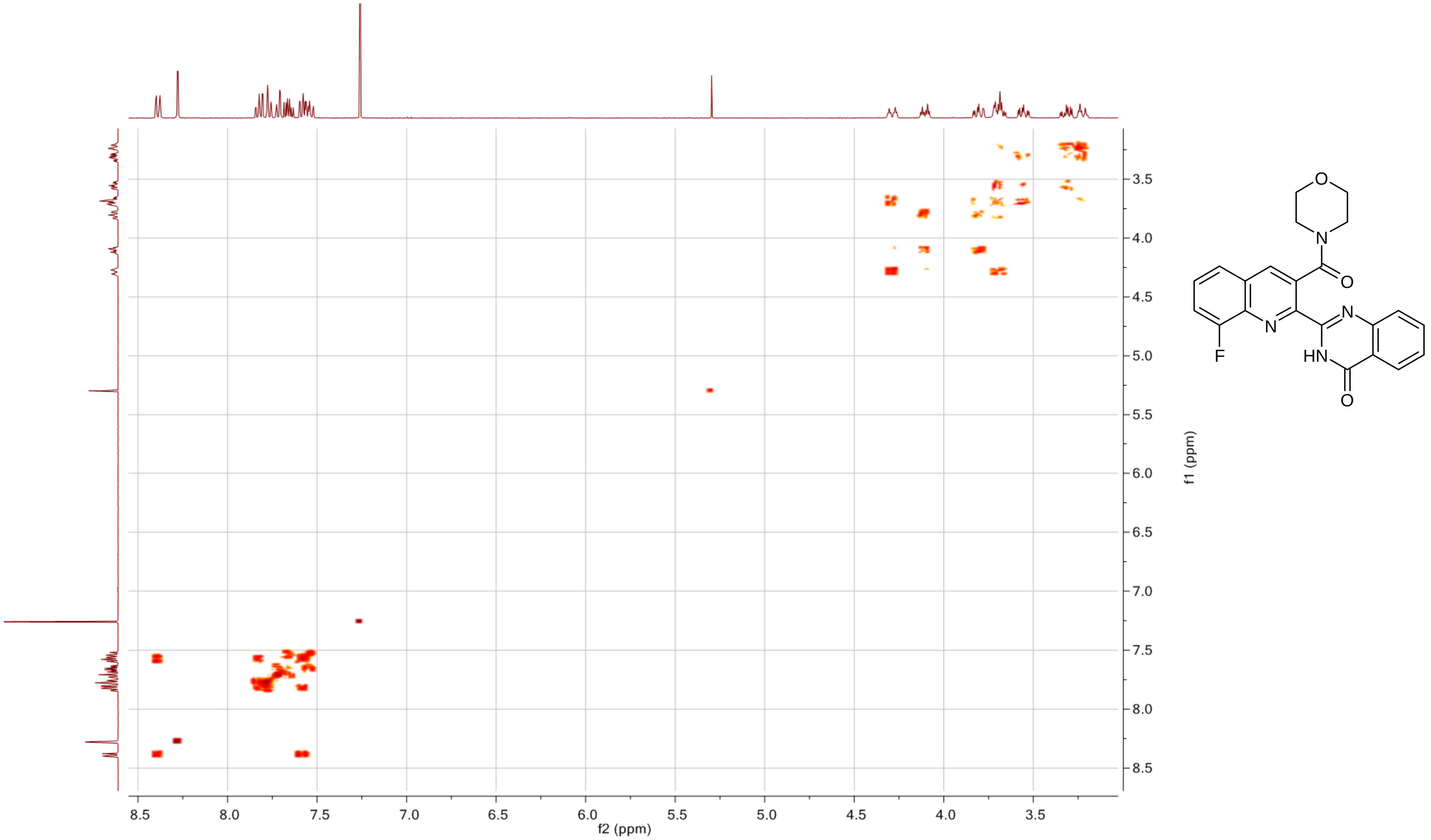

Figure S37. COSY spectrum of 2-[8-fluoro-3-(morpholin-4-ylcarbonyl)quinolin-2-yl]quinazolin-4(3*H*)-one (**7**)

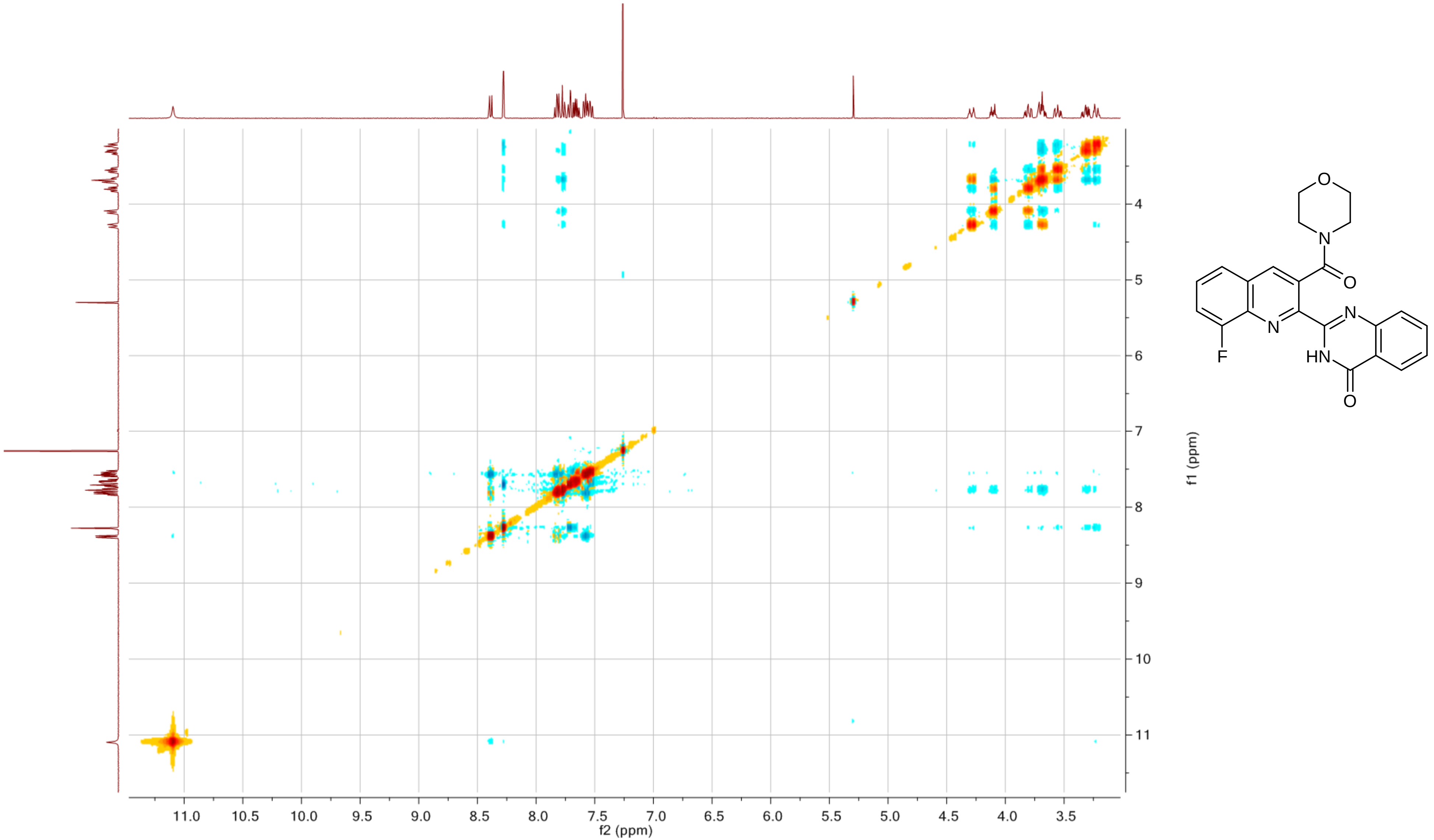

Figure S38. NOESY spectrum of 2-[8-fluoro-3-(morpholin-4-ylcarbonyl)quinolin-2-yl]quinazolin-4(3*H*)-one (**7**)

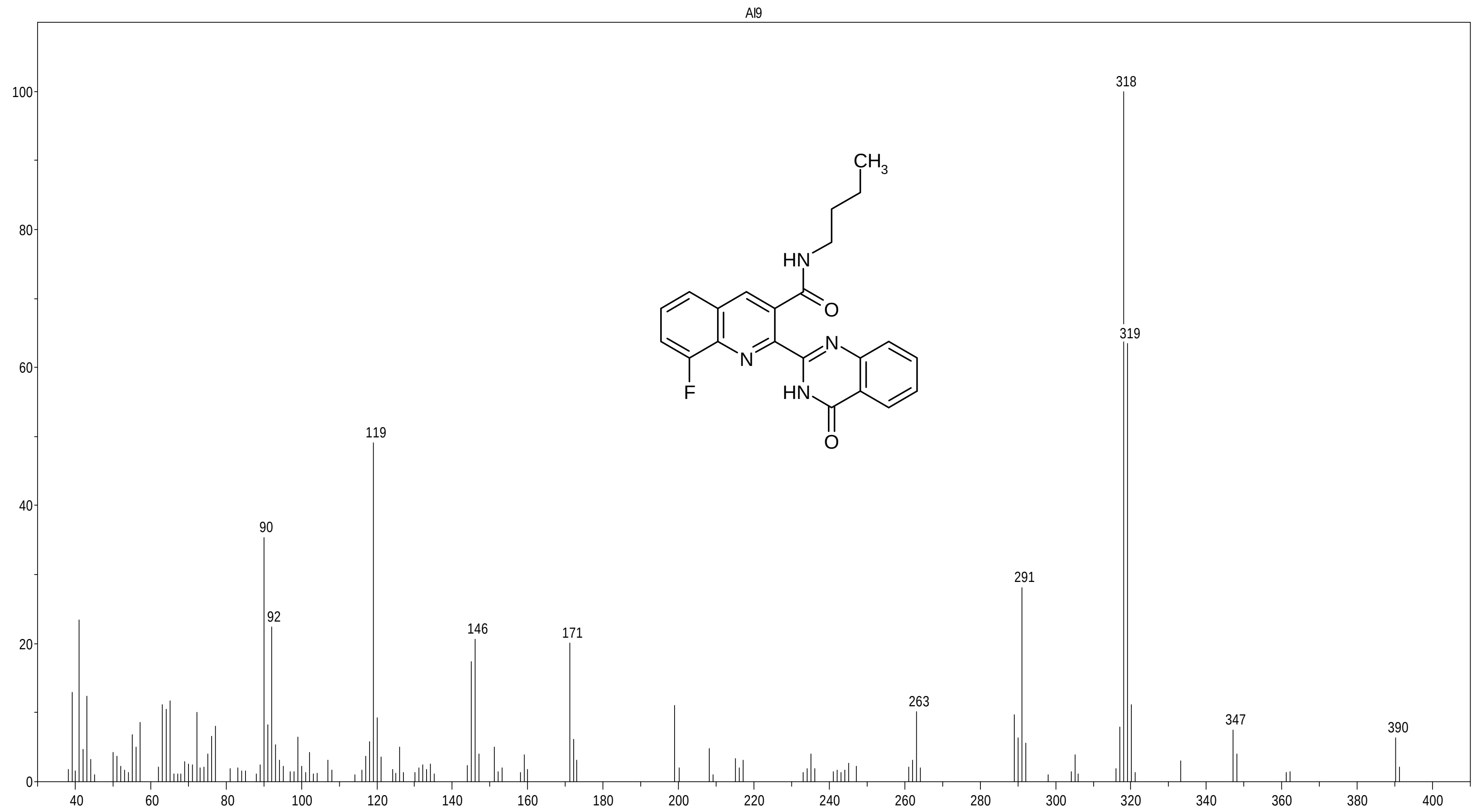

Figure S39. EI-MS of *N*-butyl-8-fluoro-2-(4-oxo-3,4-dihydroquinazolin-2-yl)quinoline-3-carboxamide (**8**)

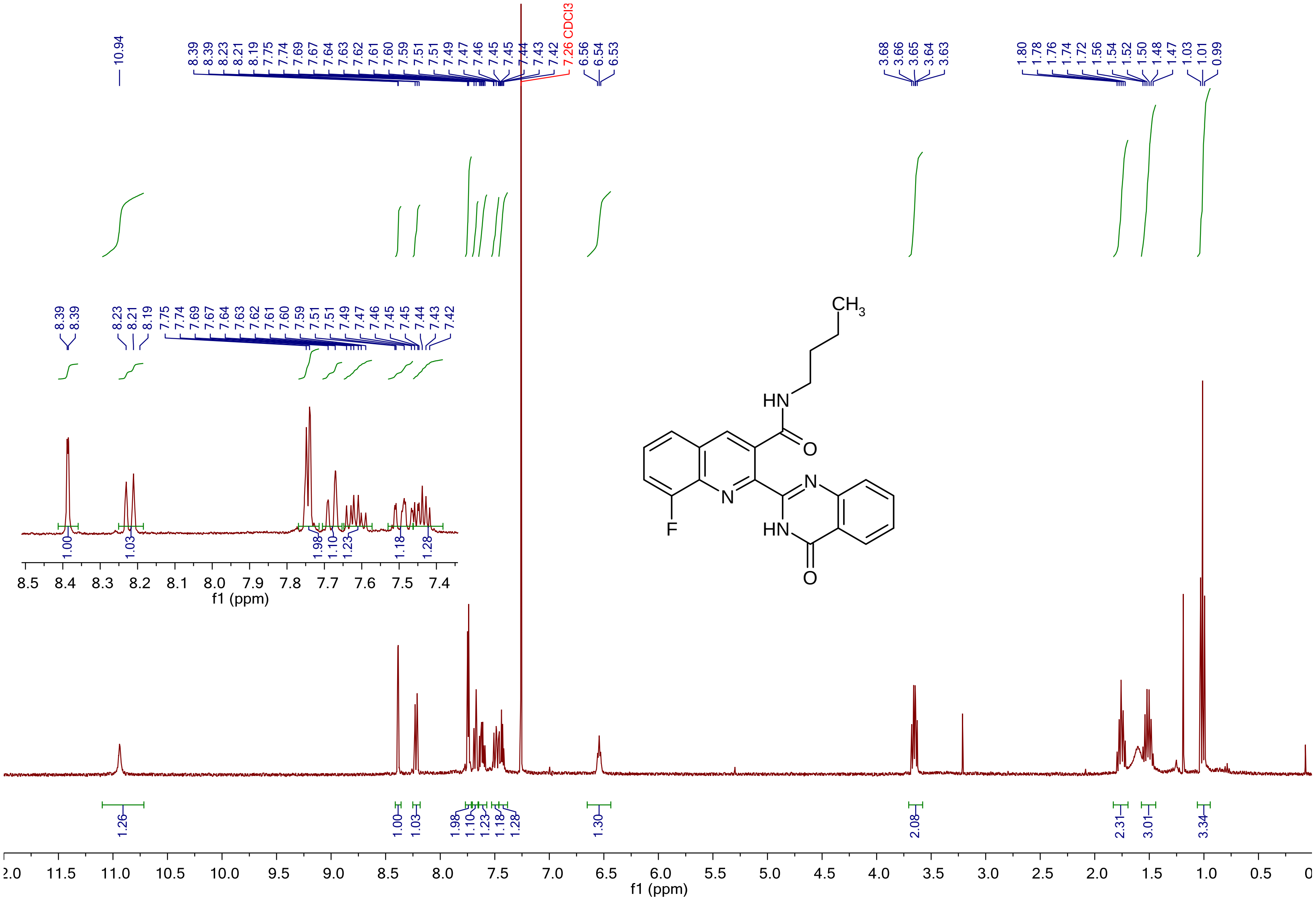

Figure S40. ^{1}H-NMR spectrum of *N*-butyl-8-fluoro-2-(4-oxo-3,4-dihydroquinazolin-2-yl)quinoline-3-carboxamide (**8**)

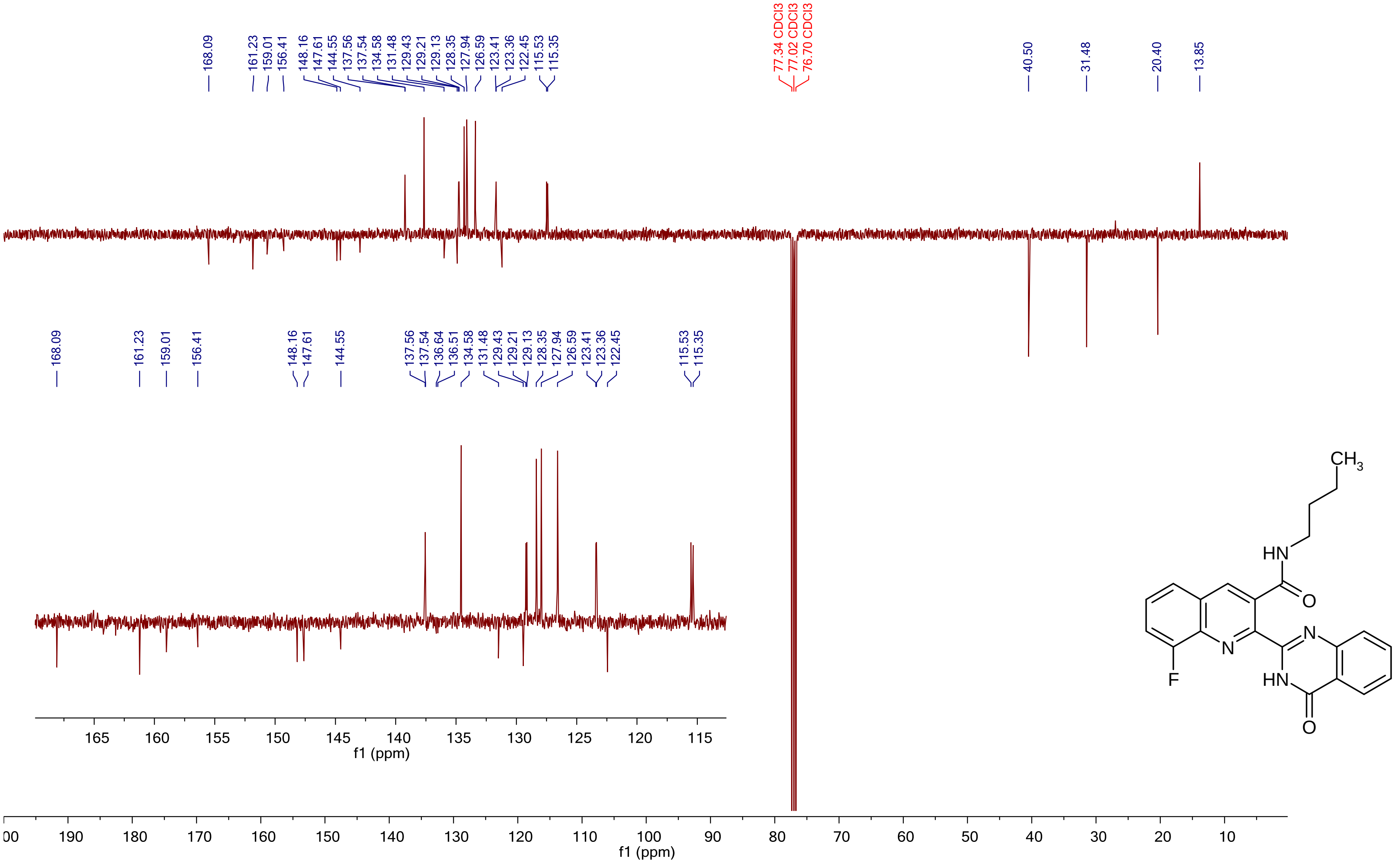

Figure S41. ^{13}C-NMR spectrum of *N*-butyl-8-fluoro-2-(4-oxo-3,4-dihydroquinazolin-2-yl)quinoline-3-carboxamide (**8**)

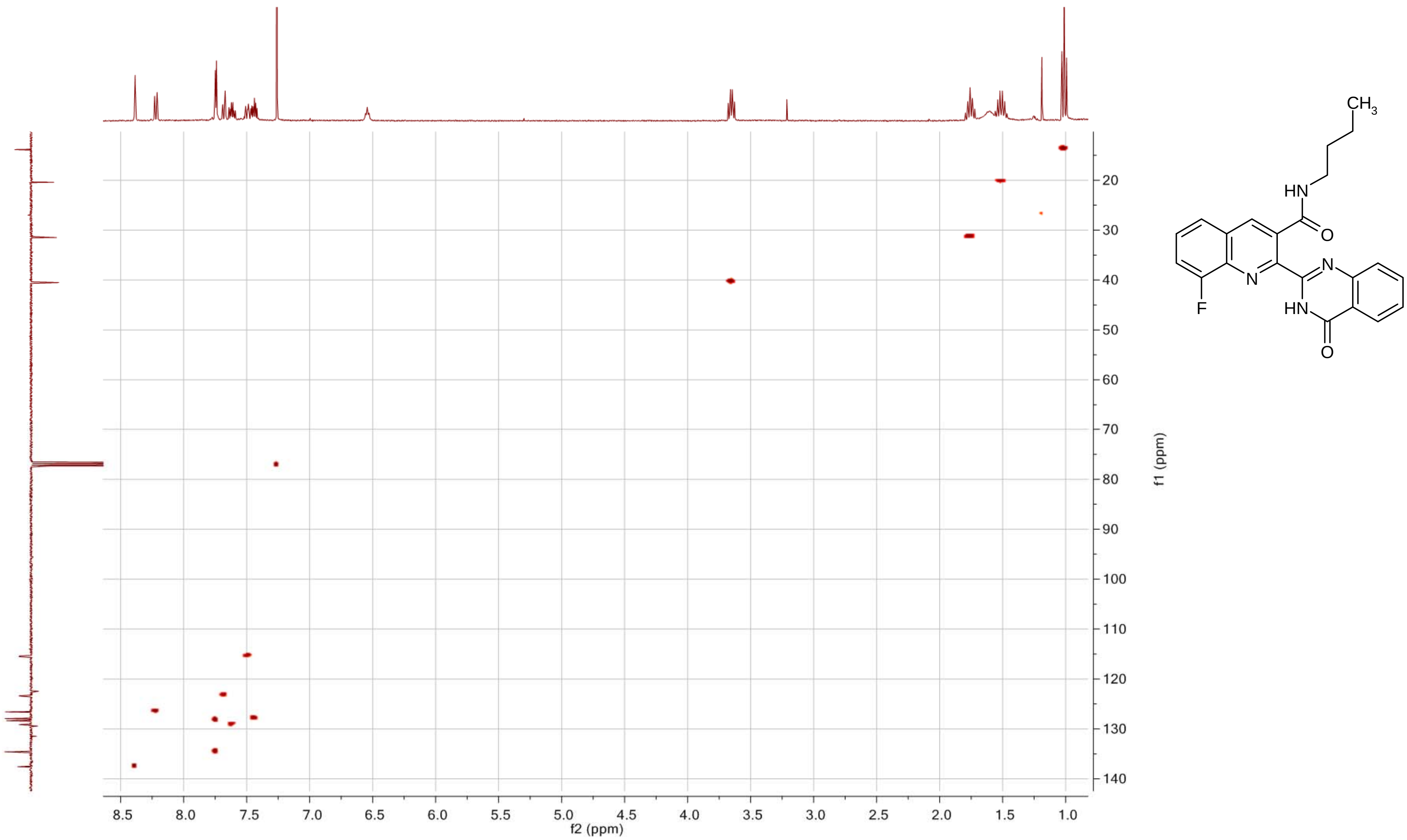

Figure S42. HSQC spectrum of *N*-butyl-8-fluoro-2-(4-oxo-3,4-dihydroquinazolin-2-yl)quinoline-3-carboxamide (**8**)

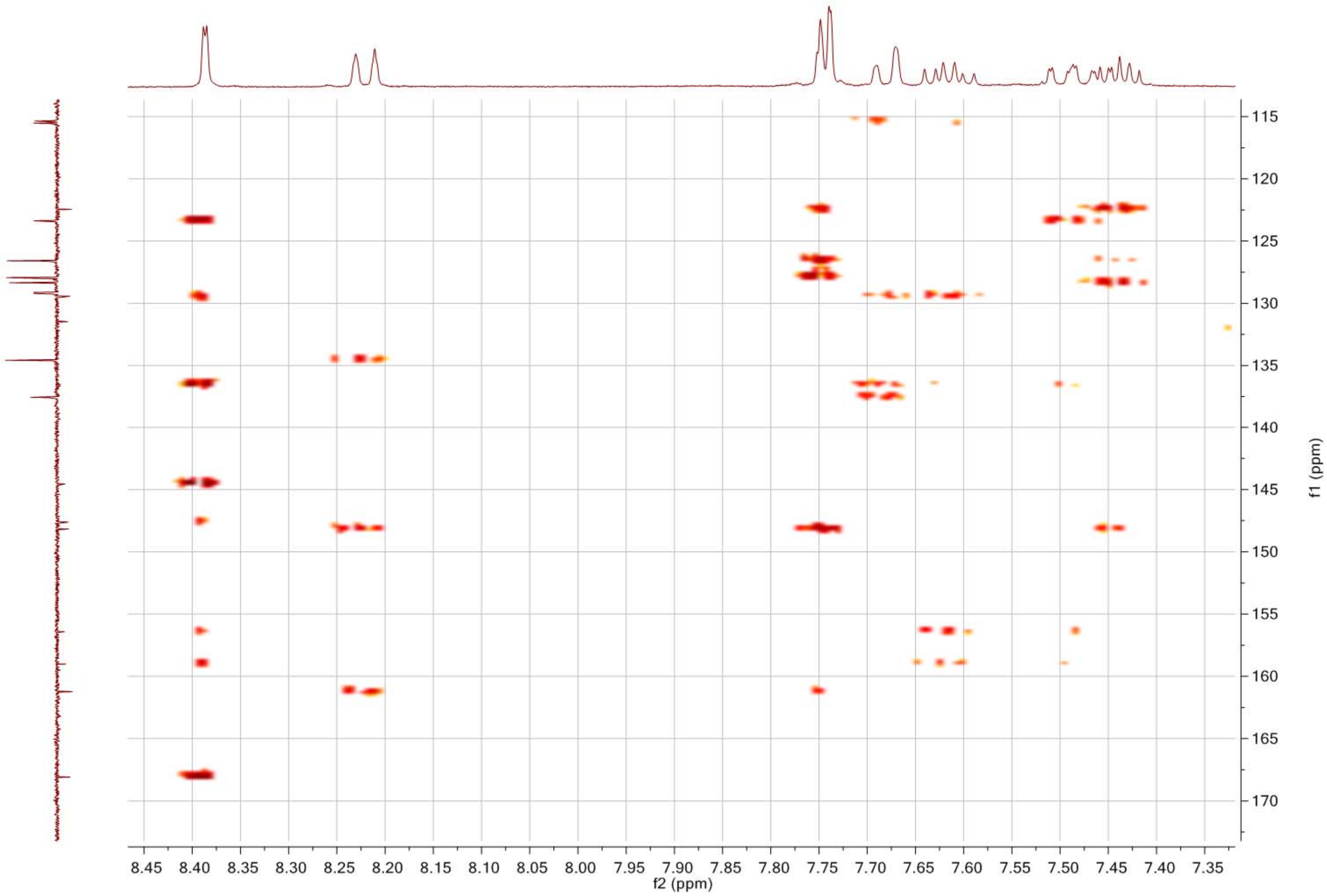

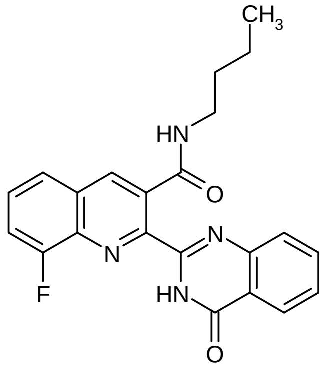

Figure S43. HMBC spectrum of *N*-butyl-8-fluoro-2-(4-oxo-3,4-dihydroquinazolin-2-yl)quinoline-3-carboxamide (**8**)

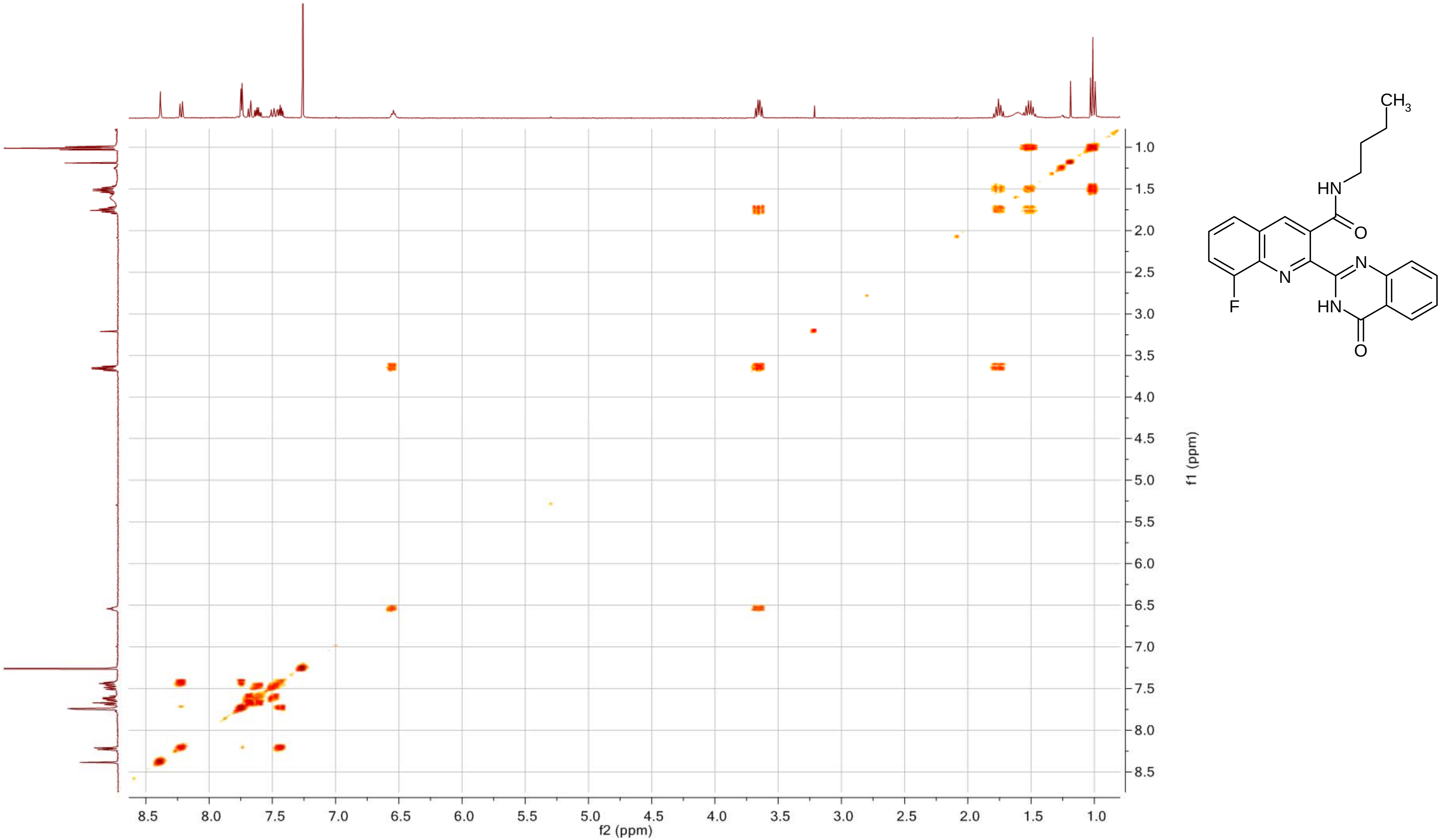

Figure S44. COSY spectrum of *N*-butyl-8-fluoro-2-(4-oxo-3,4-dihydroquinazolin-2-yl)quinoline-3-carboxamide (**8**)

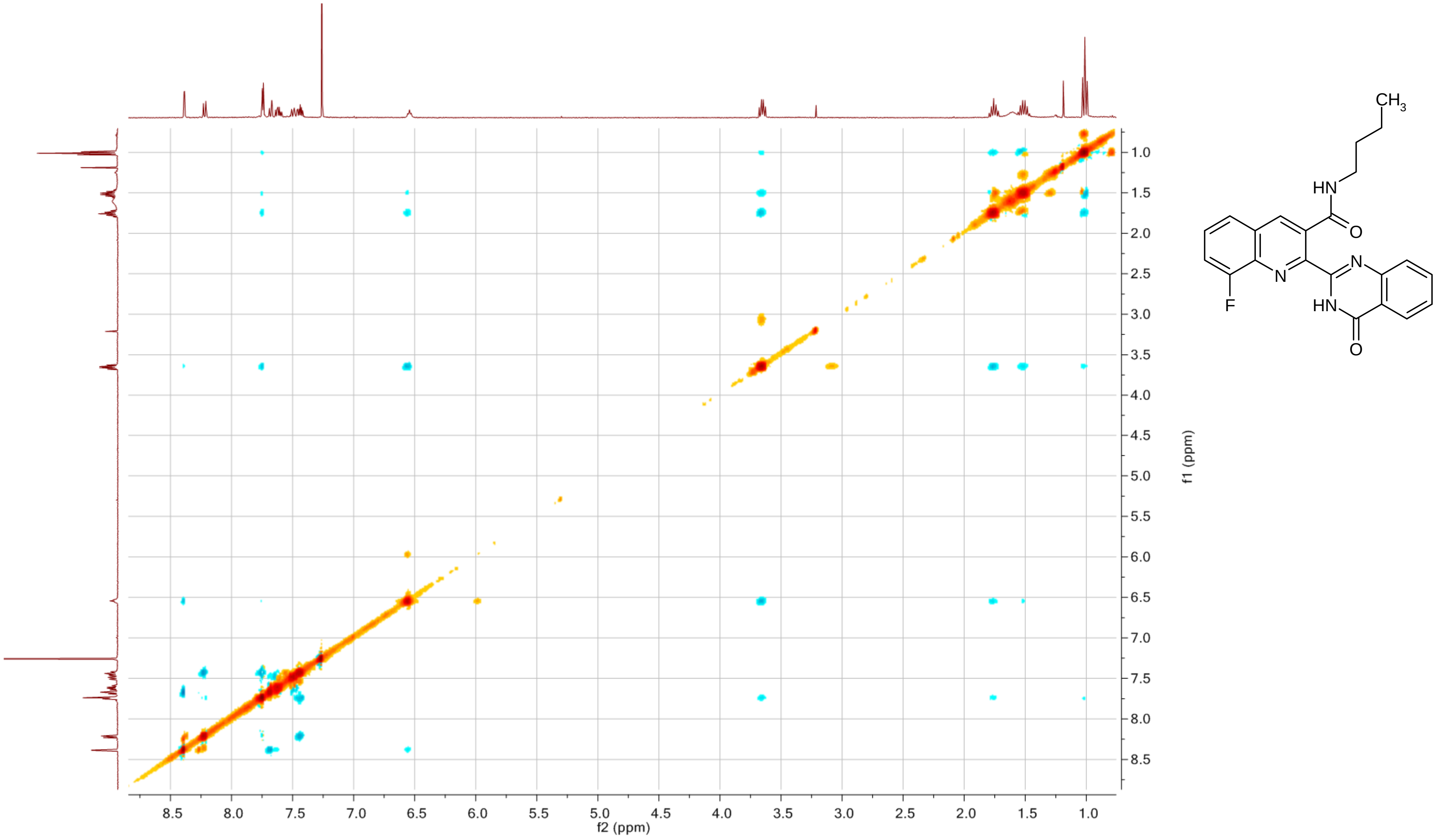

Figure S45. NOESY spectrum of *N*-butyl-8-fluoro-2-(4-oxo-3,4-dihydroquinazolin-2-yl)quinoline-3-carboxamide (**8**)

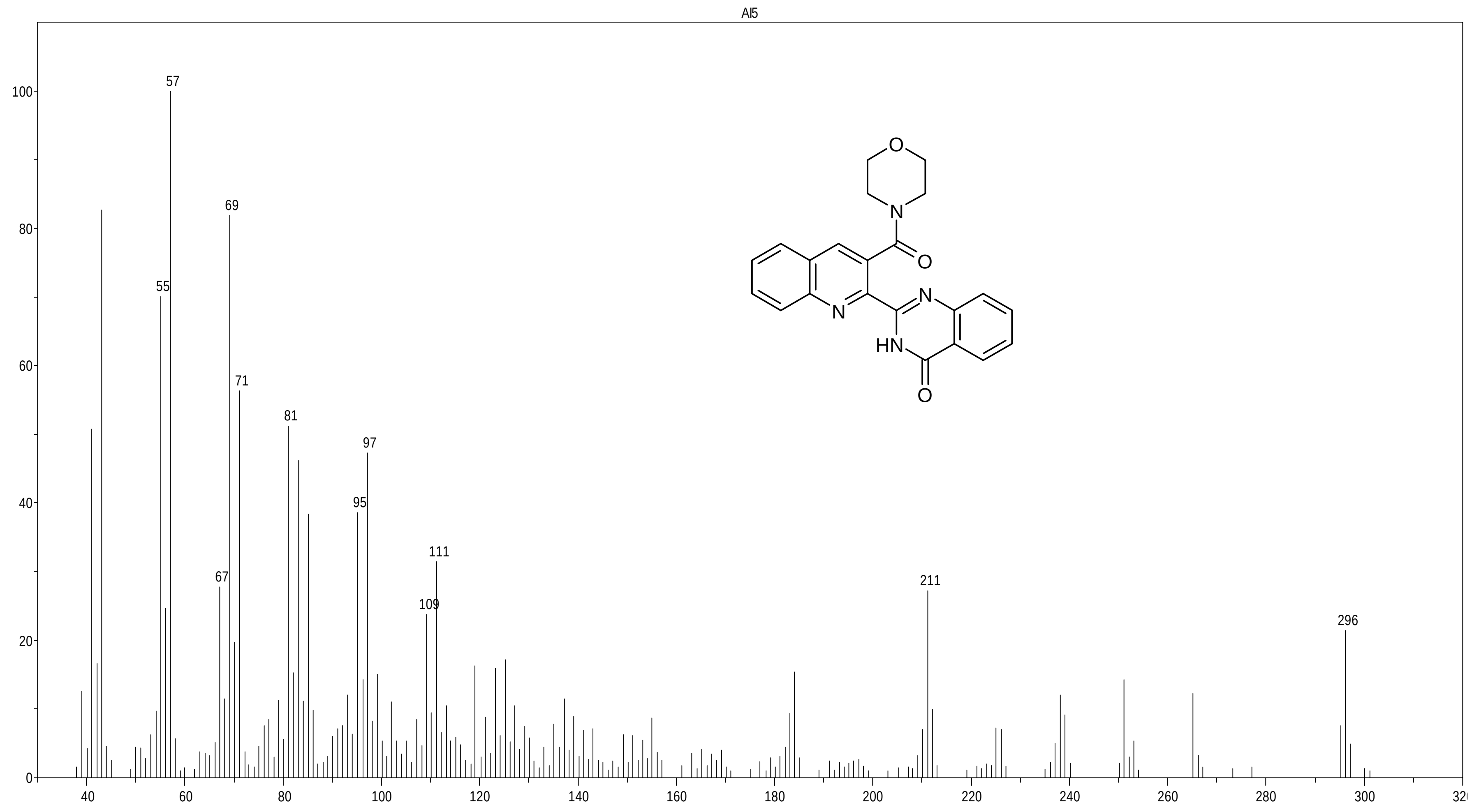

Figure S46. EI-MS of 2-[3-(morpholin-4-ylcarbonyl)quinolin-2-yl]quinazolin-4(3*H*)-one (**10**)

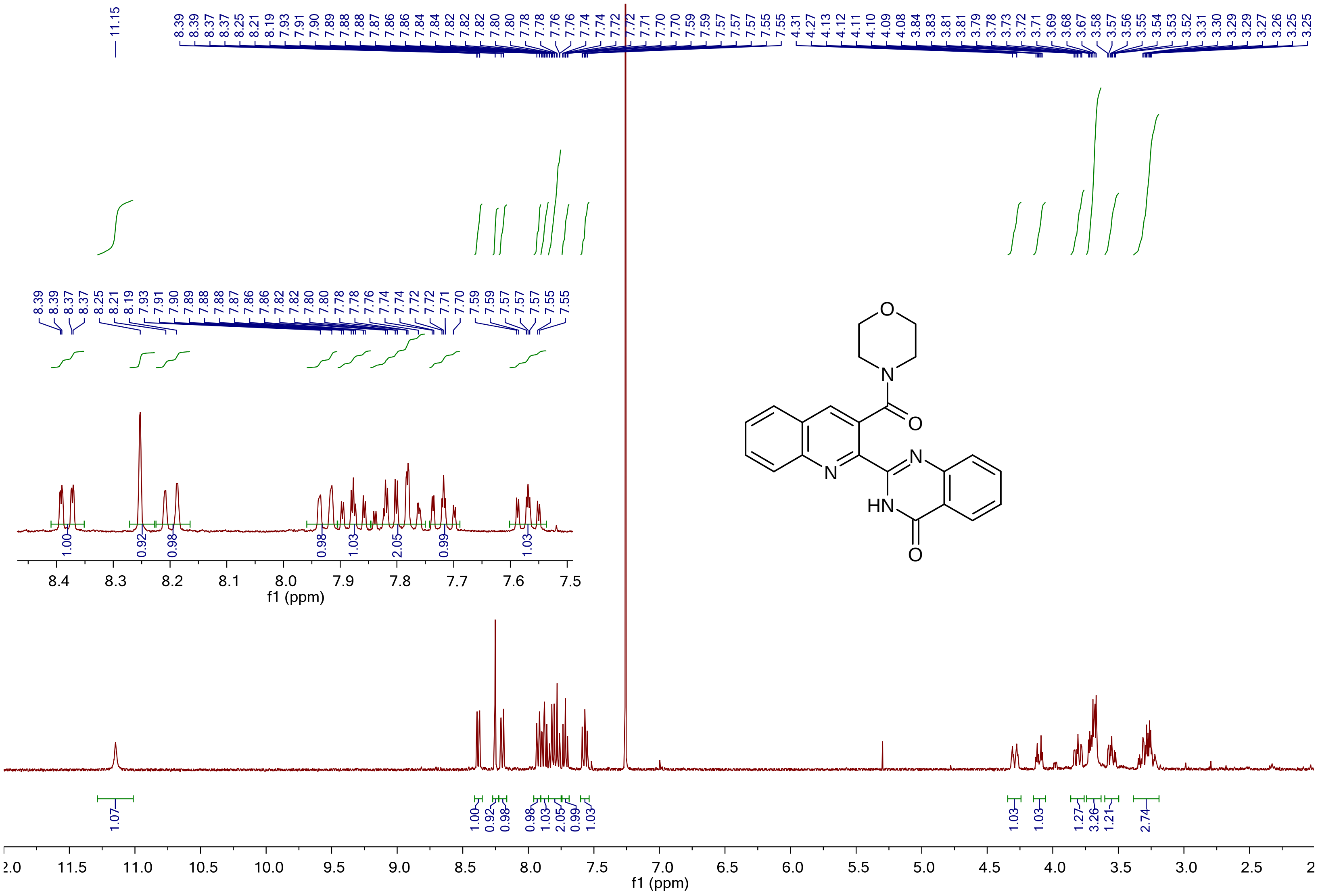

Figure S47. ^{1}H-NMR spectrum of 2-[3-(morpholin-4-ylcarbonyl)quinolin-2-yl]quinazolin-4(3*H*)-one (**10**)

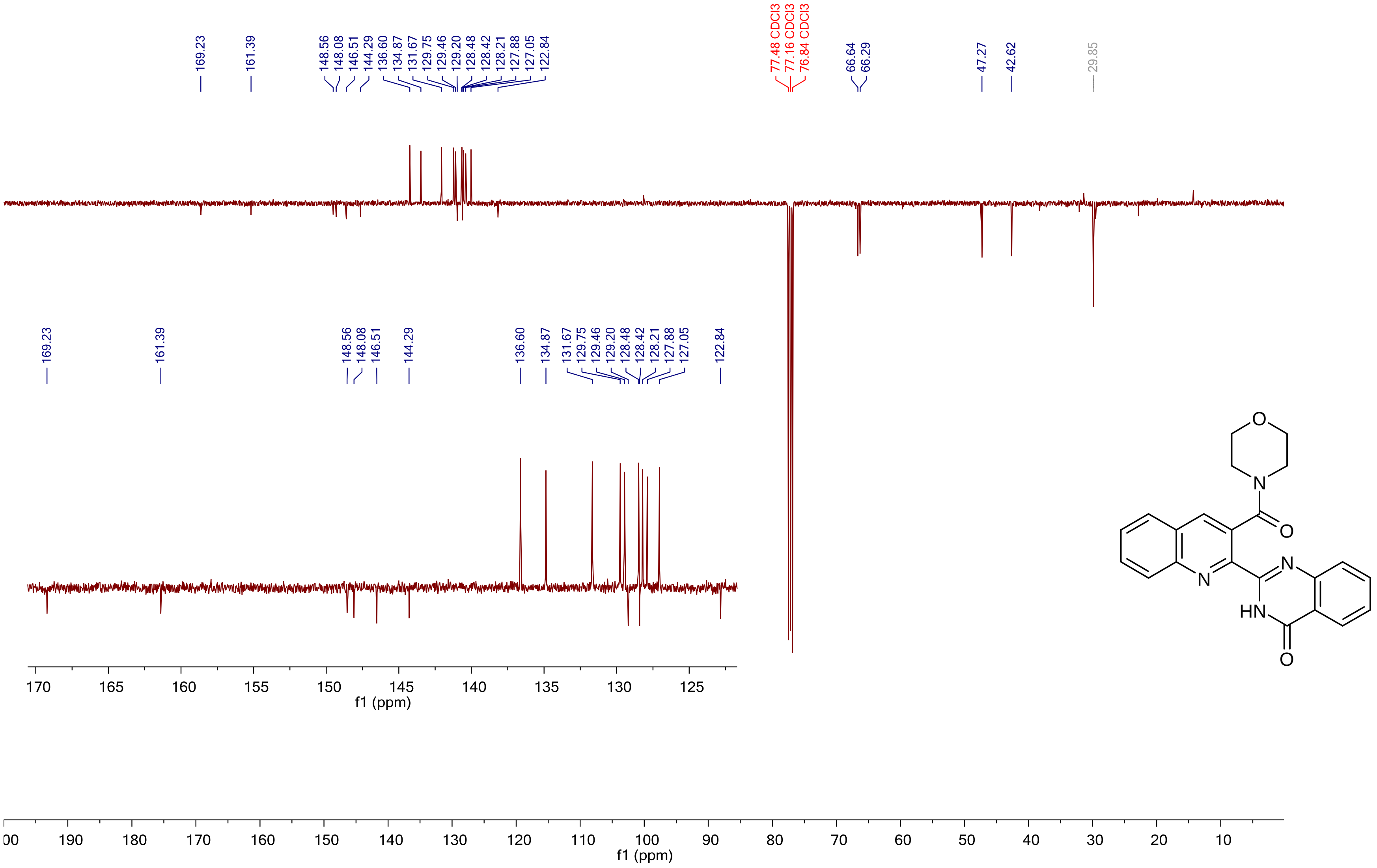

Figure S48. ^{13}C-NMR spectrum of 2-[3-(morpholin-4-ylcarbonyl)quinolin-2-yl]quinazolin-4(3*H*)-one (**10**)

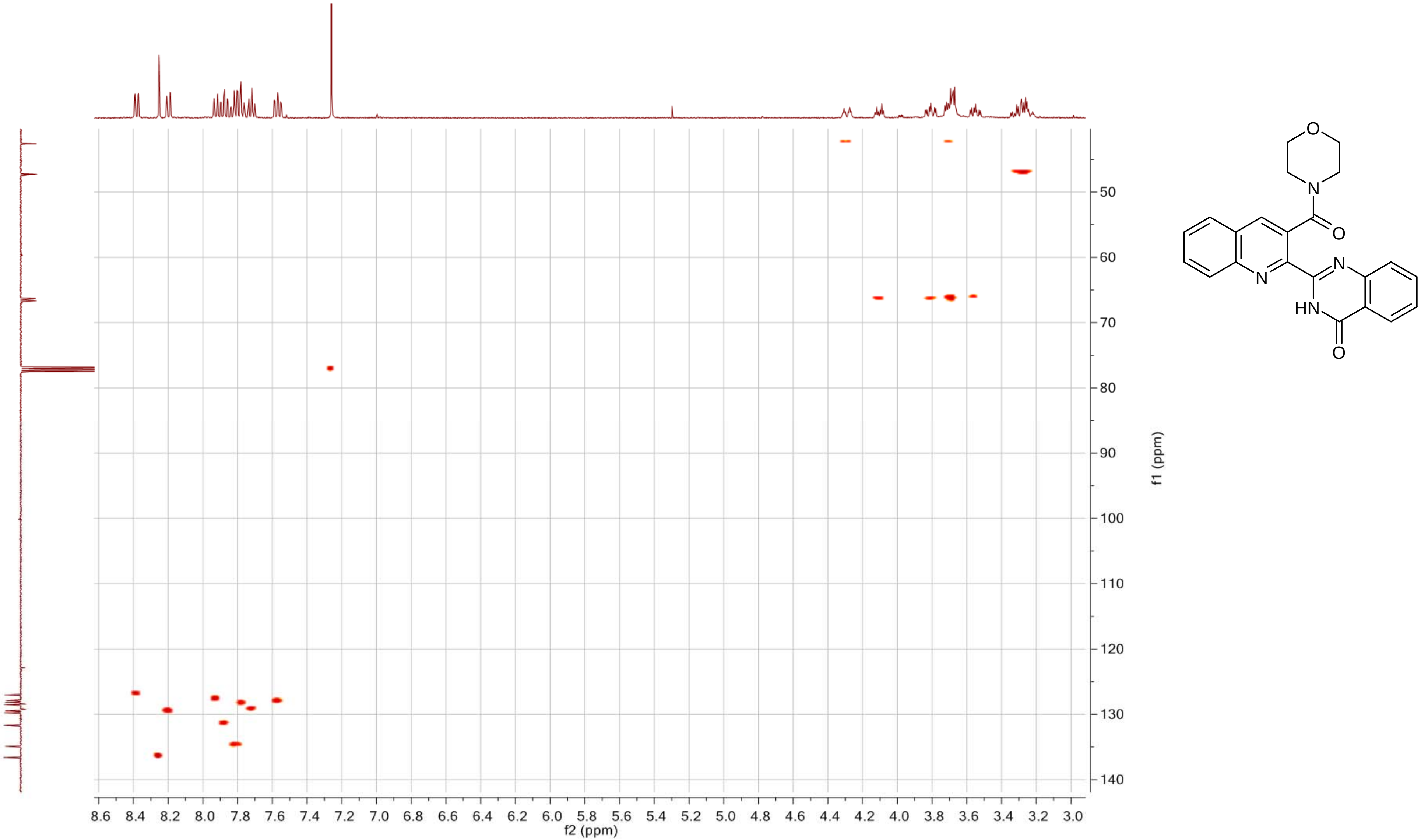

Figure S49. HSQC spectrum of 2-[3-(morpholin-4-ylcarbonyl)quinolin-2-yl]quinazolin-4(3*H*)-one (**10**)

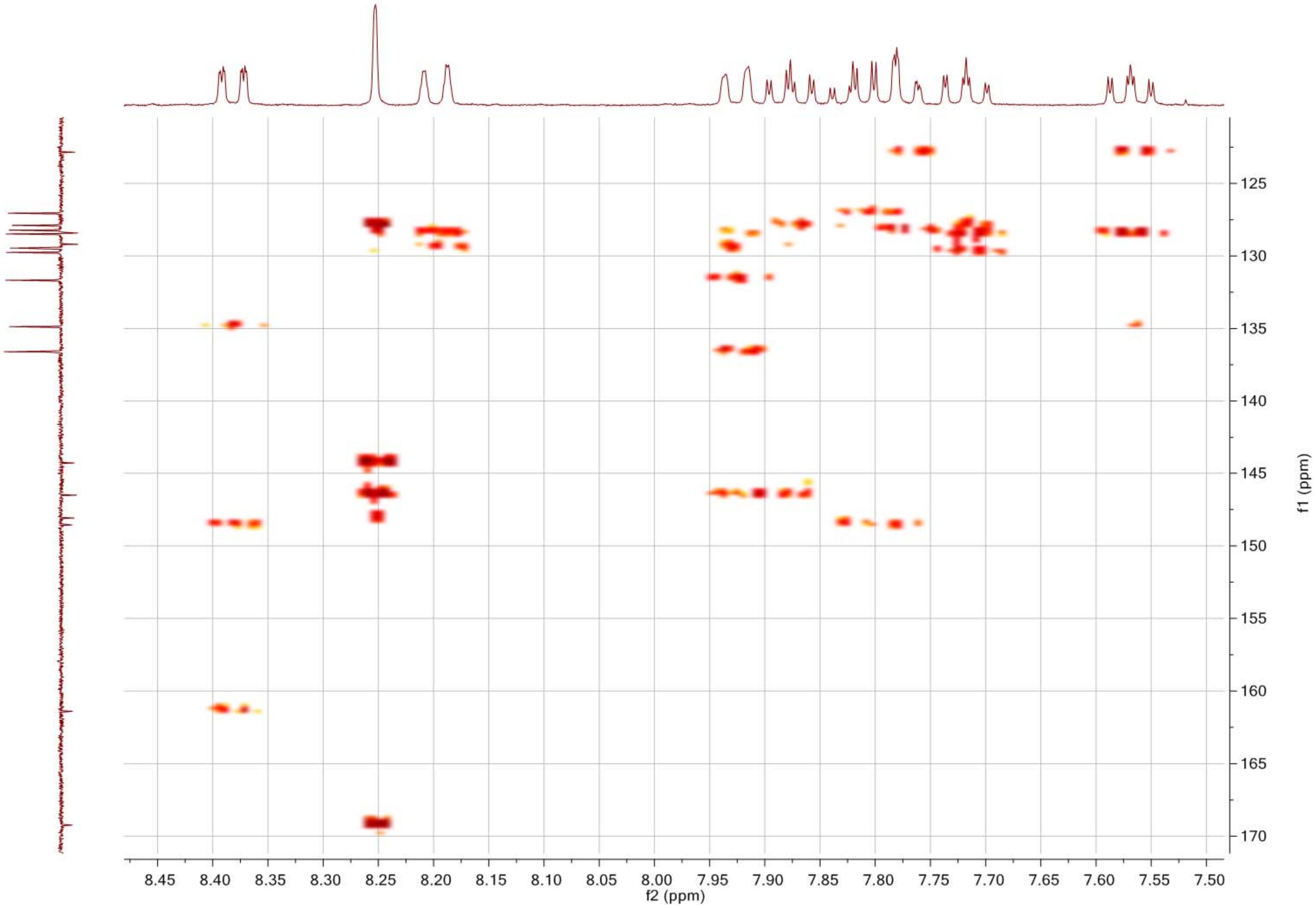

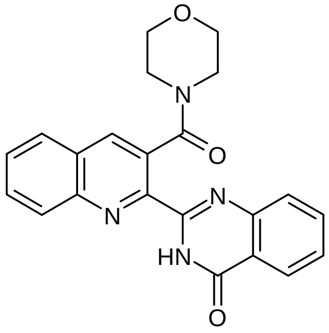

Figure S50. HMBC spectrum of 2-[3-(morpholin-4-ylcarbonyl)quinolin-2-yl]quinazolin-4(3*H*)-one (**10**)

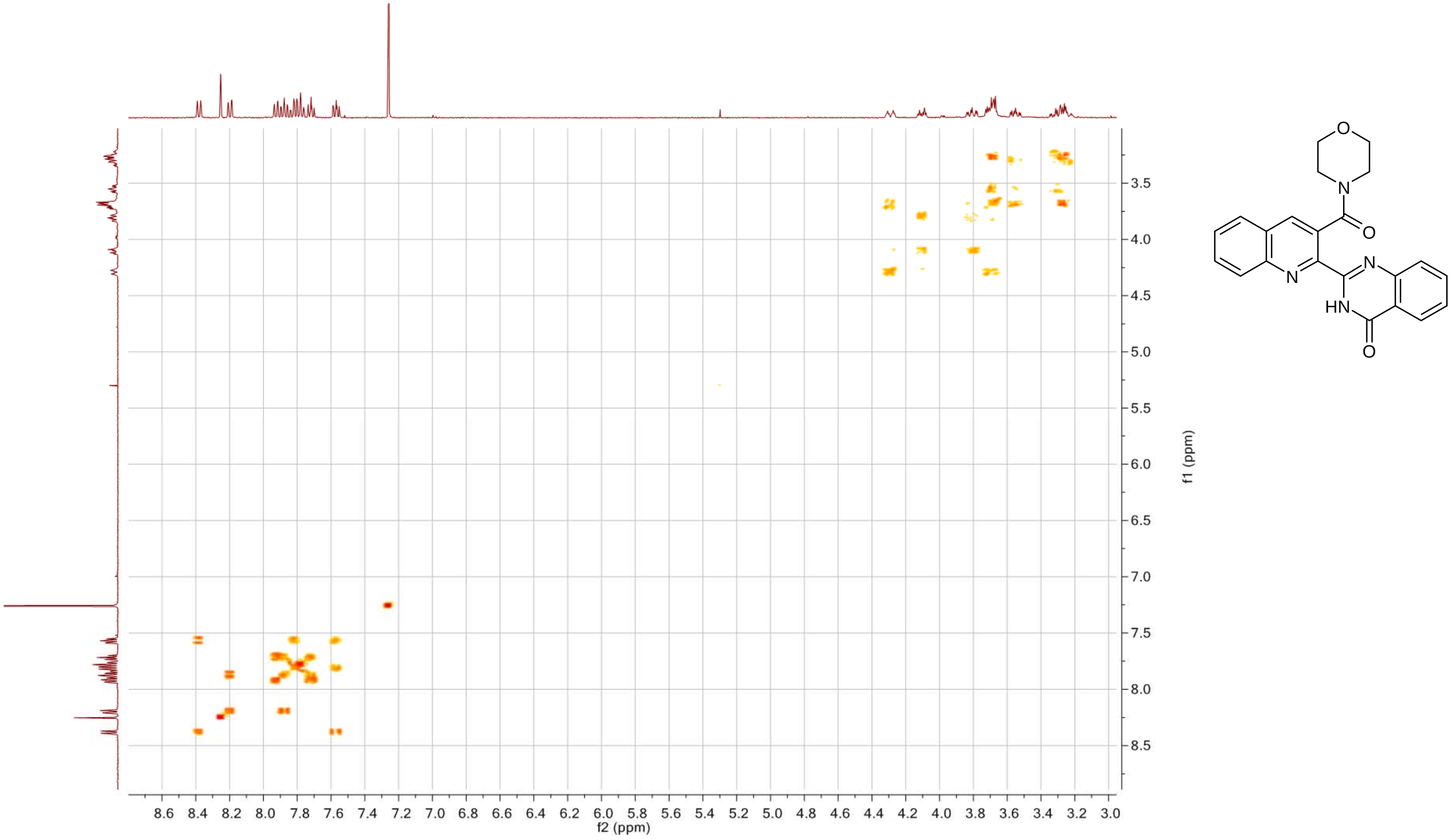

Figure S51. COSY spectrum of 2-[3-(morpholin-4-ylcarbonyl)quinolin-2-yl]quinazolin-4(3*H*)-one (**10**)

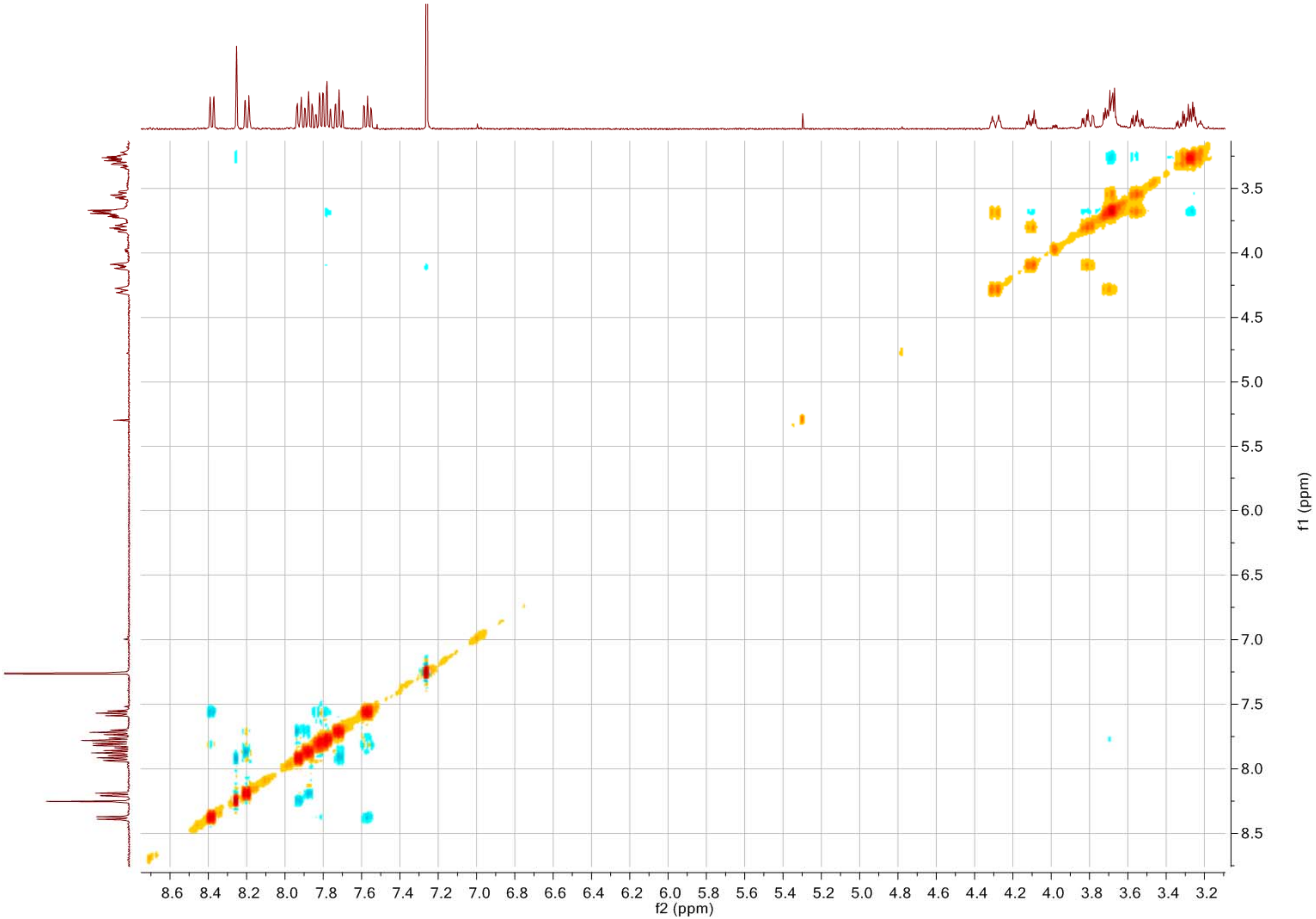

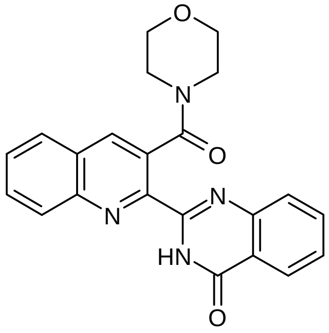

Figure S52. NOESY spectrum of 2-[3-(morpholin-4-ylcarbonyl)quinolin-2-yl]quinazolin-4(3*H*)-one (**10**)

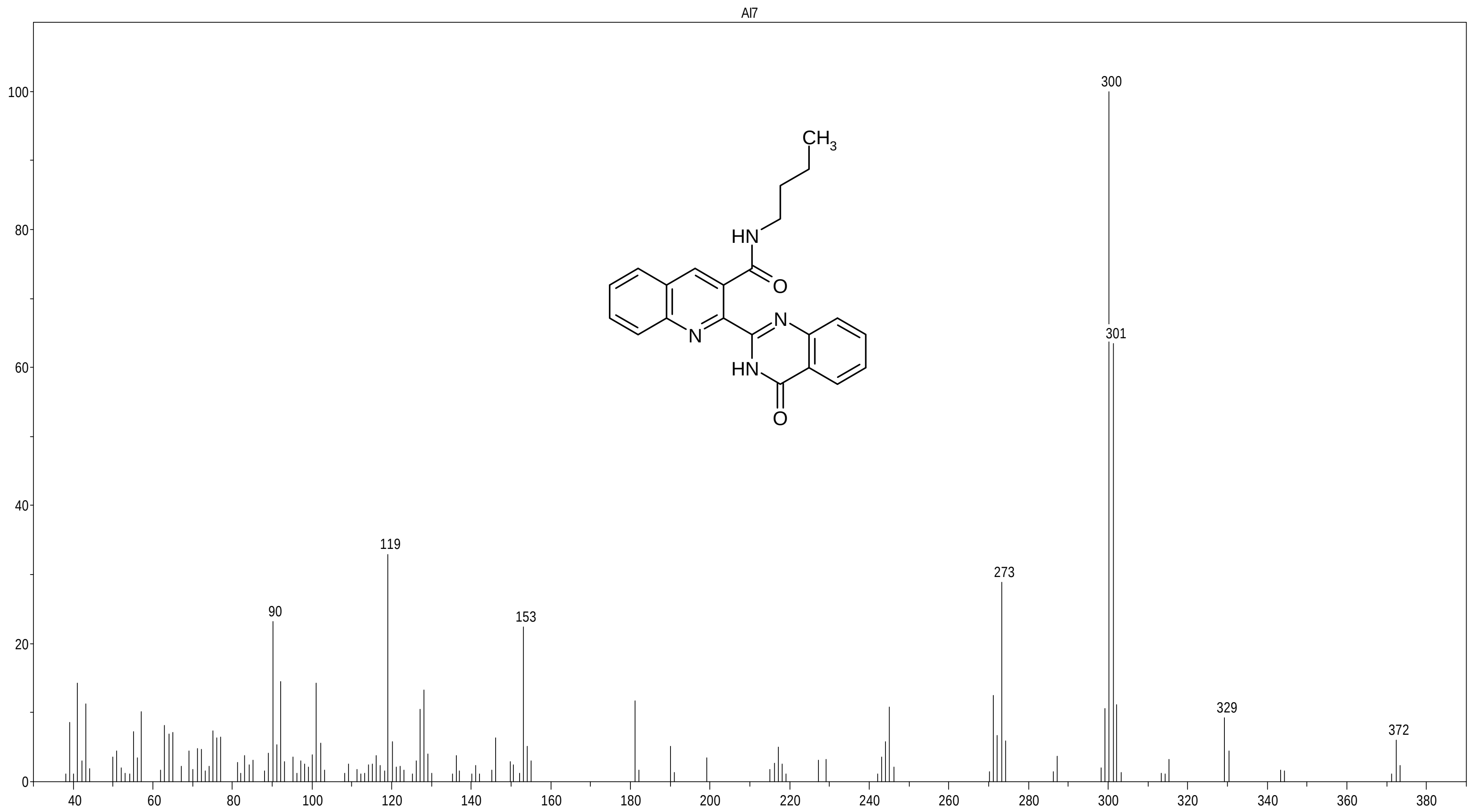

Figure S53. EI-MS of *N*-butyl-2-(4-oxo-3,4-dihydroquinazolin-2-yl)quinoline-3-carboxamide (**11**)

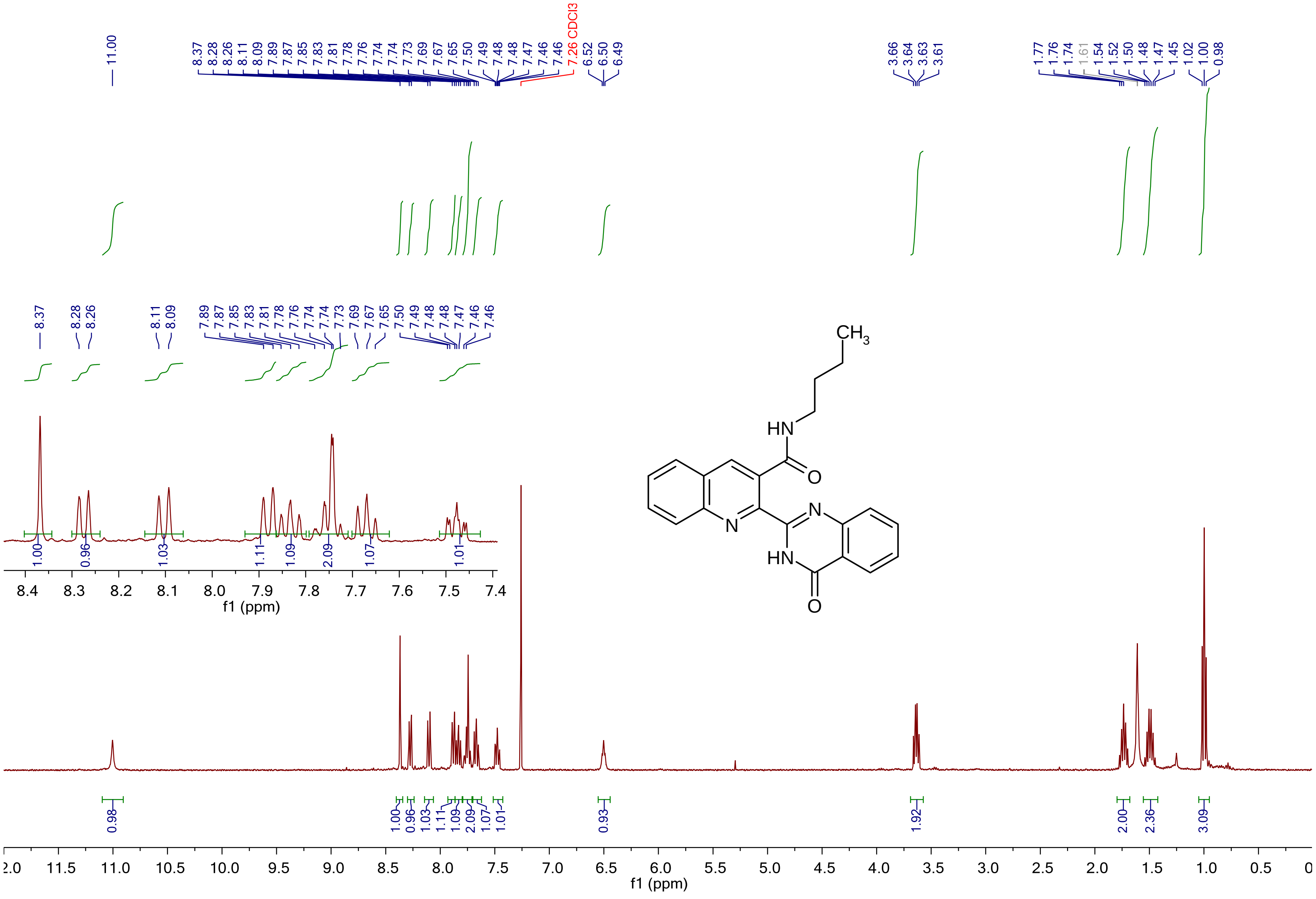

Figure S54. ^{1}H-NMR spectrum of *N*-butyl-2-(4-oxo-3,4-dihydroquinazolin-2-yl)quinoline-3-carboxamide (**11**)

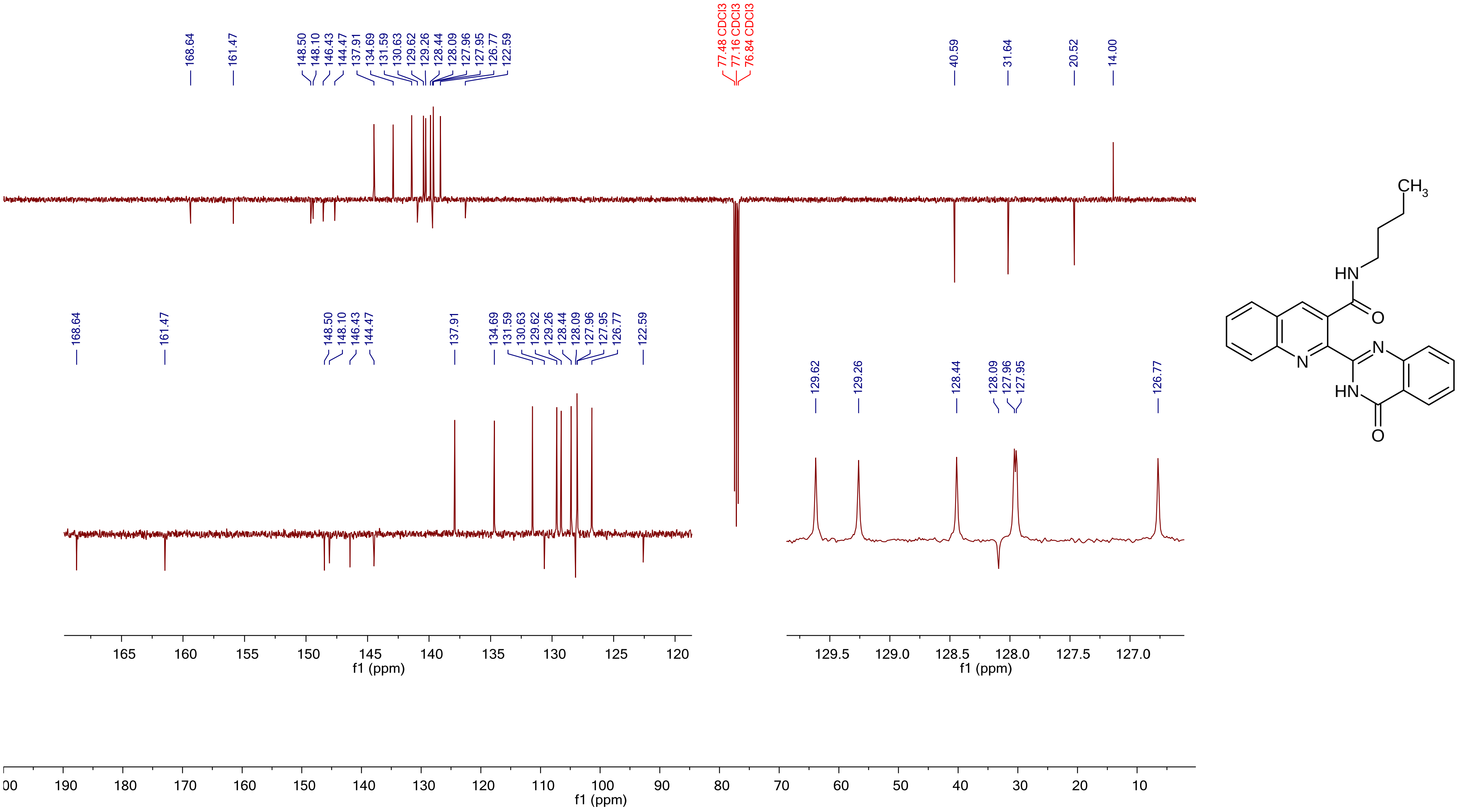

Figure S55. ^{13}C-NMR spectrum of *N*-butyl-2-(4-oxo-3,4-dihydroquinazolin-2-yl)quinoline-3-carboxamide (**11**)

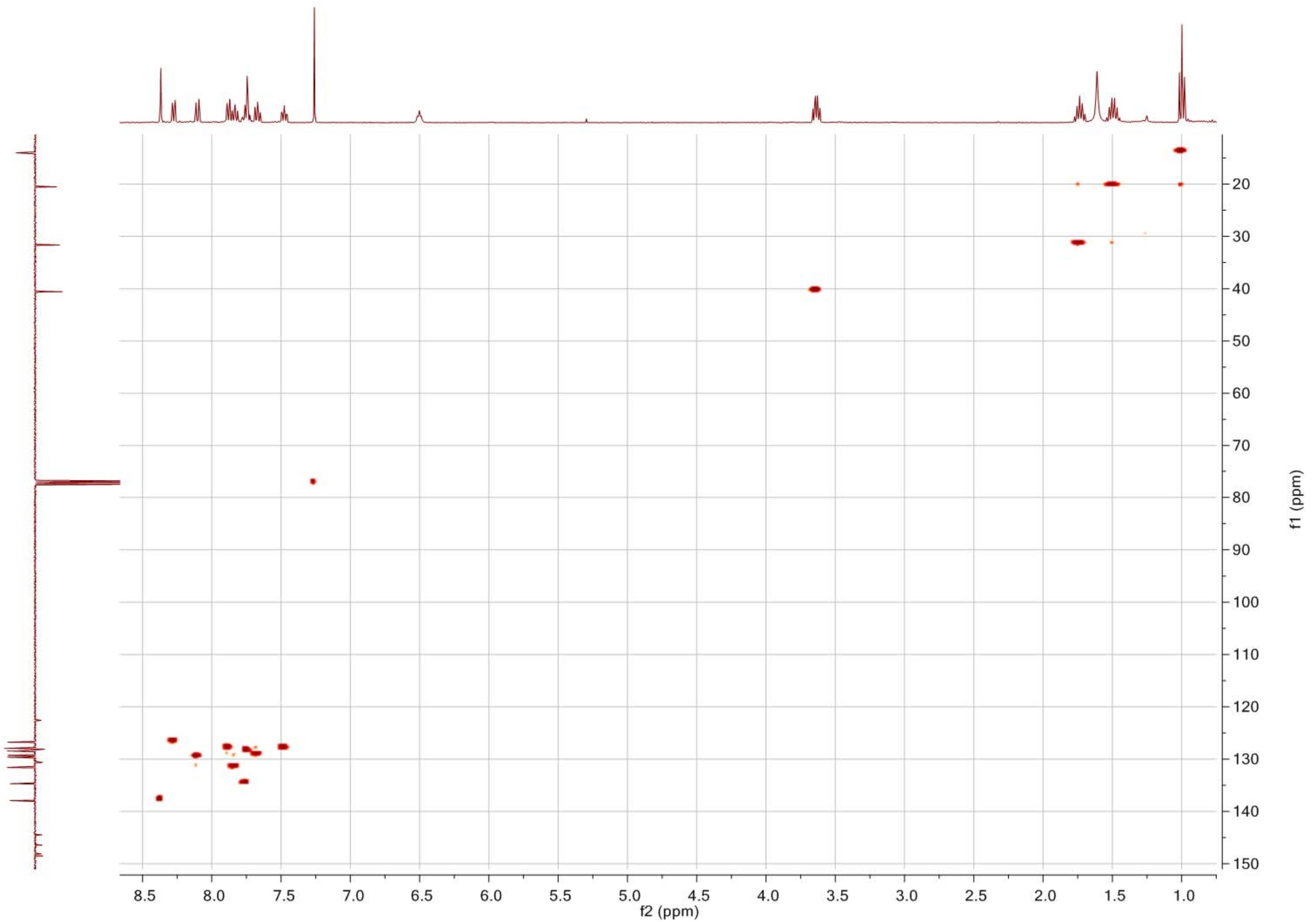

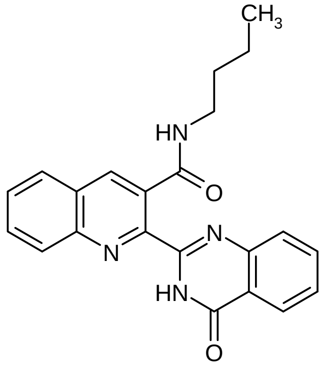

Figure S56. HSQC spectrum of *N*-butyl-2-(4-oxo-3,4-dihydroquinazolin-2-yl)quinoline-3-carboxamide (**11**)

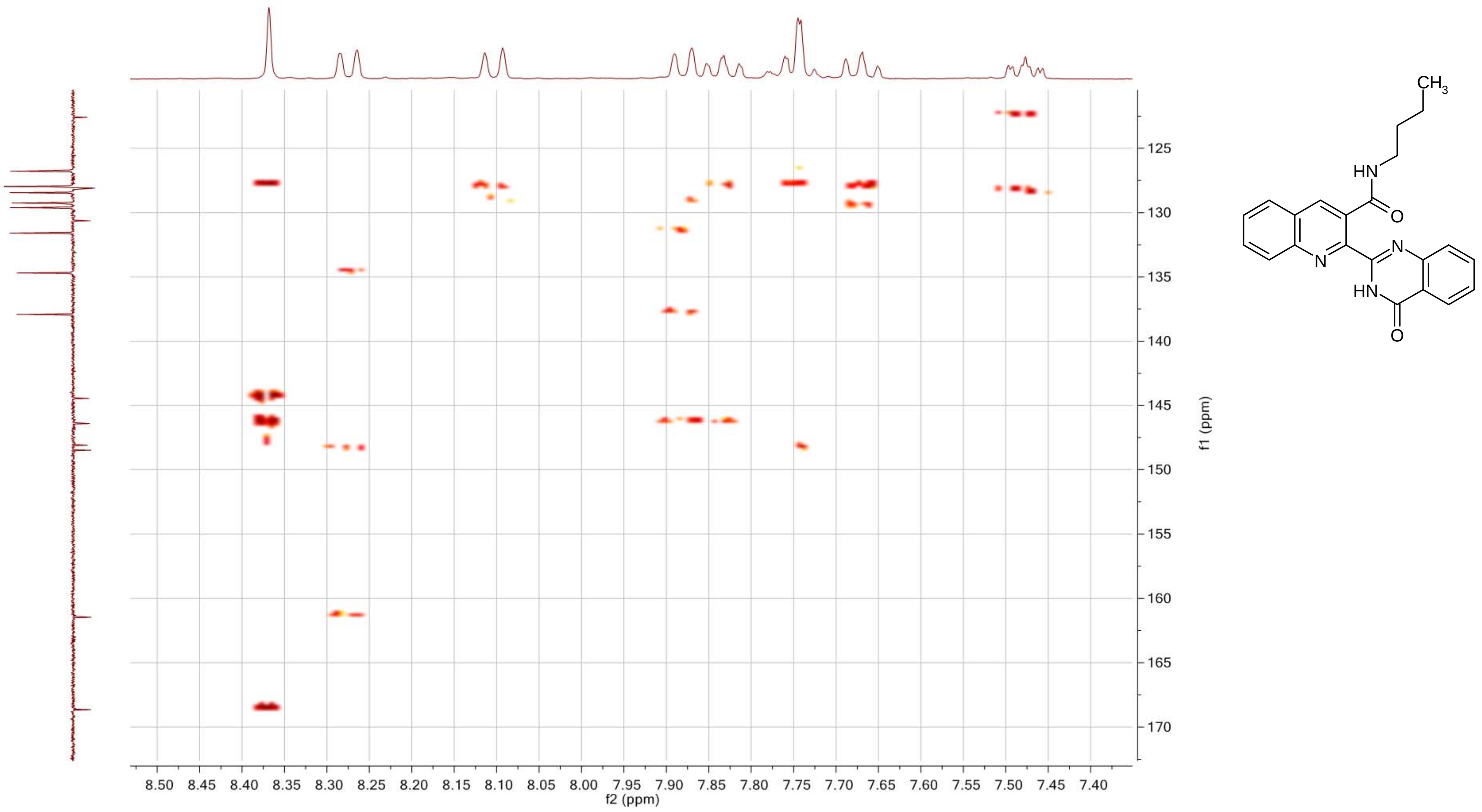

Figure S57. HMBC spectrum of *N*-butyl-2-(4-oxo-3,4-dihydroquinazolin-2-yl)quinoline-3-carboxamide (**11**)

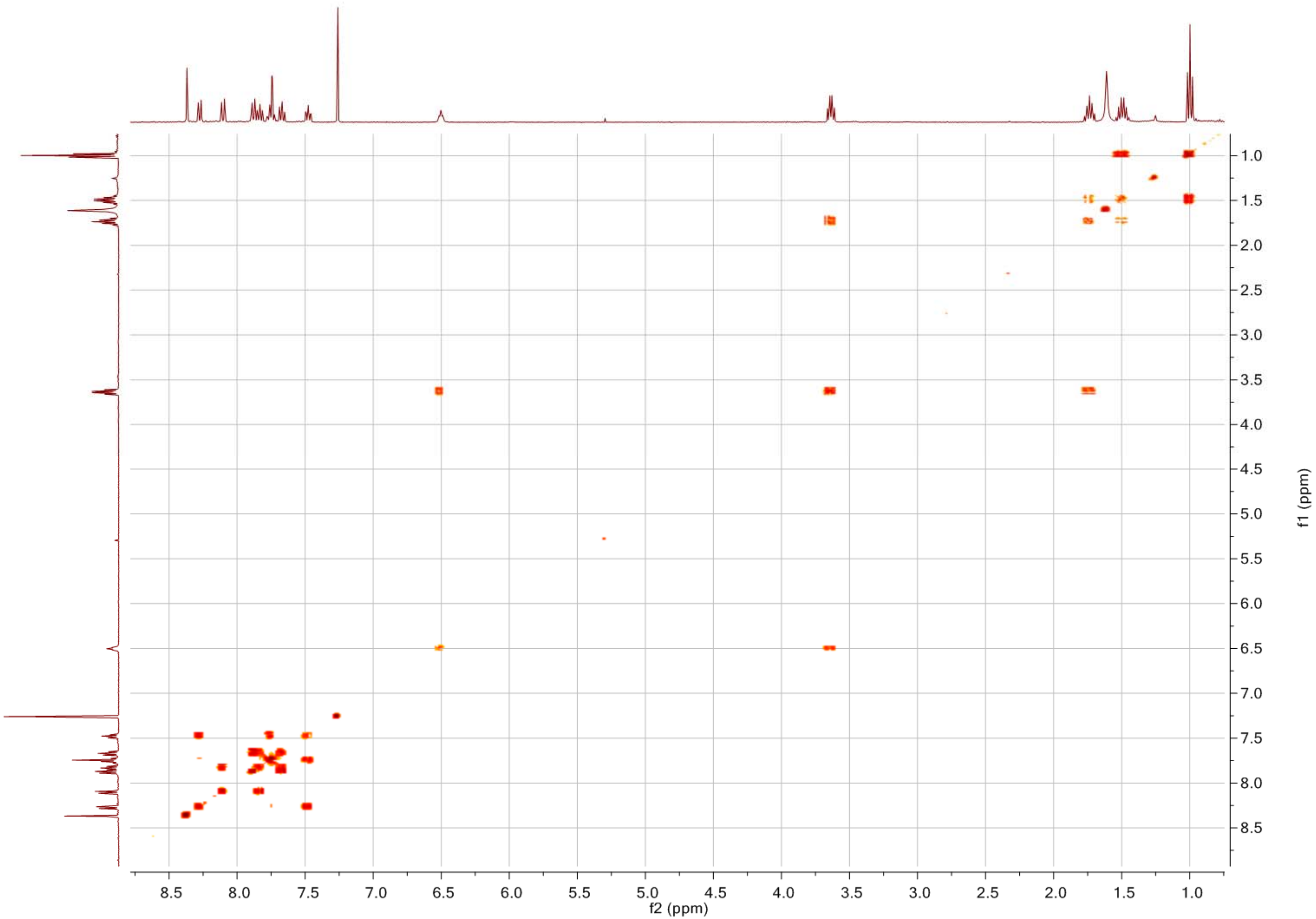

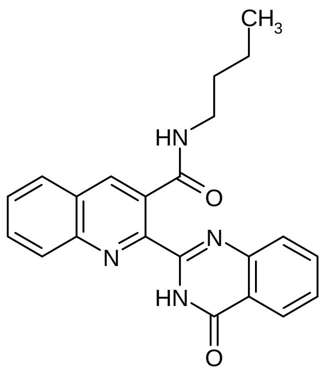

Figure S58. COSY spectrum of *N*-butyl-2-(4-oxo-3,4-dihydroquinazolin-2-yl)quinoline-3-carboxamide (**11**)

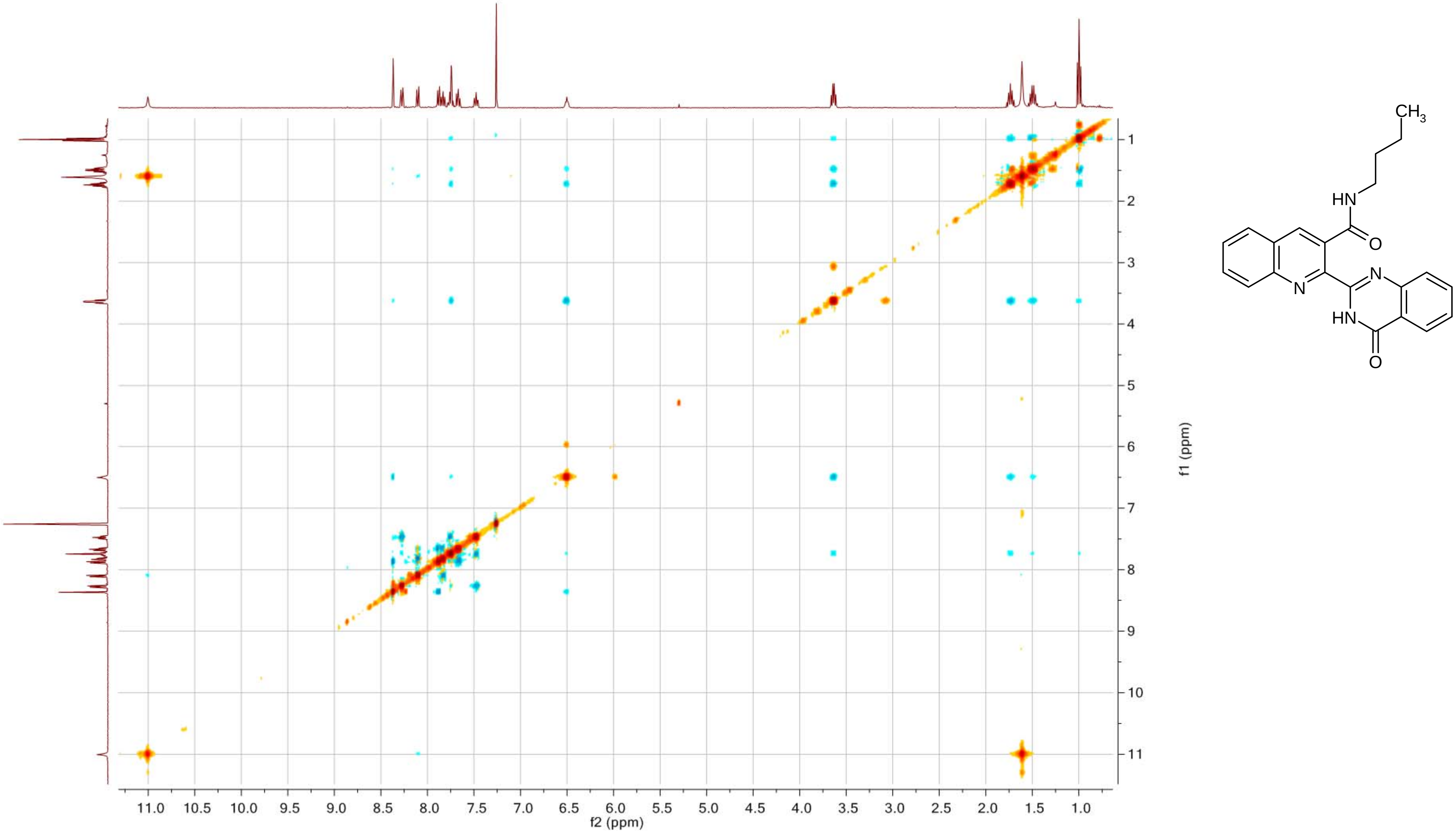

Figure S59. NOESY spectrum of *N*-butyl-2-(4-oxo-3,4-dihydroquinazolin-2-yl)quinoline-3-carboxamide (**11**)

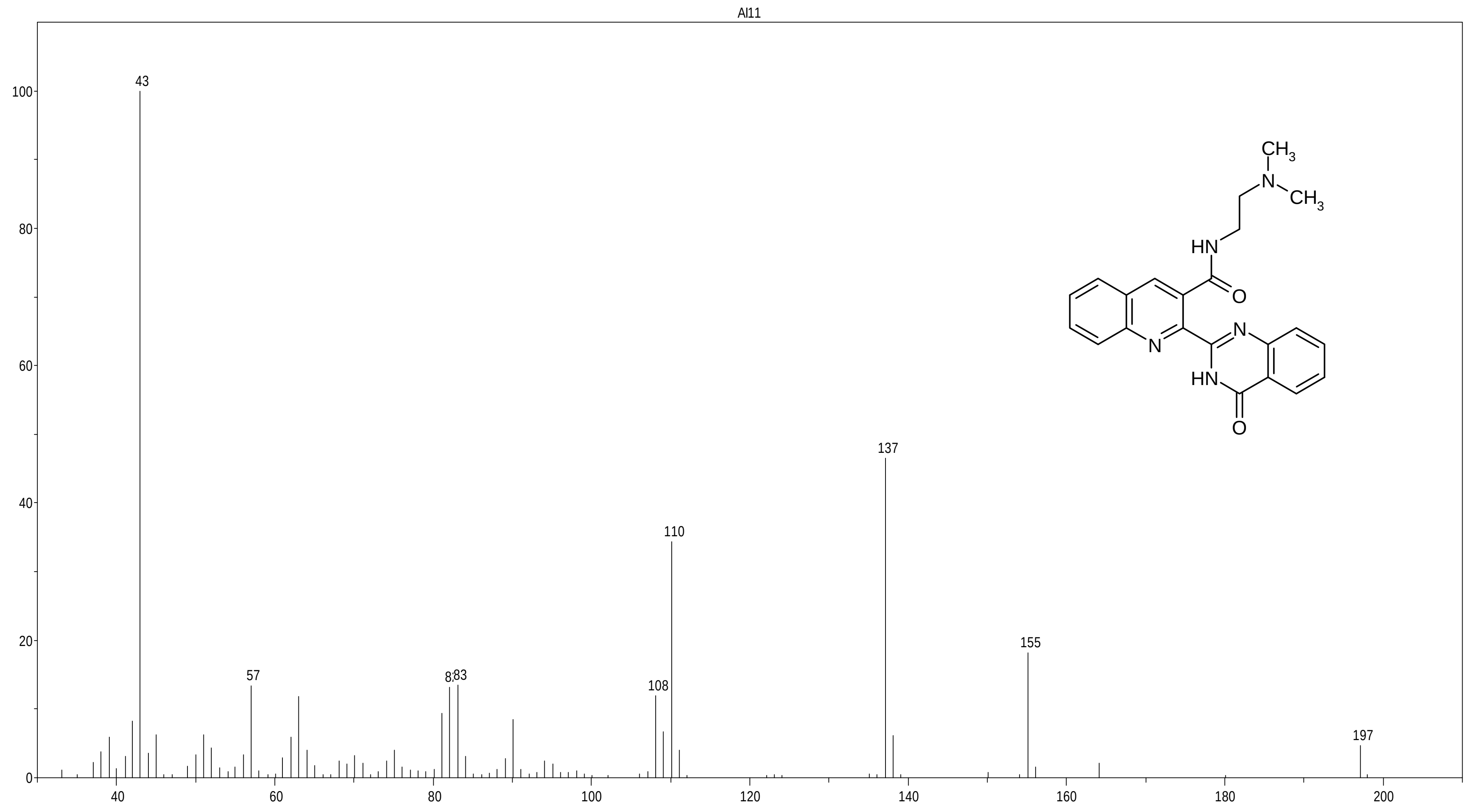

Figure S60. EI-MS of *N*-[2-(dimethylamino)ethyl]-2-(4-oxo-3,4-dihydroquinazolin-2-yl)quinoline-3-carboxamide (**12**)

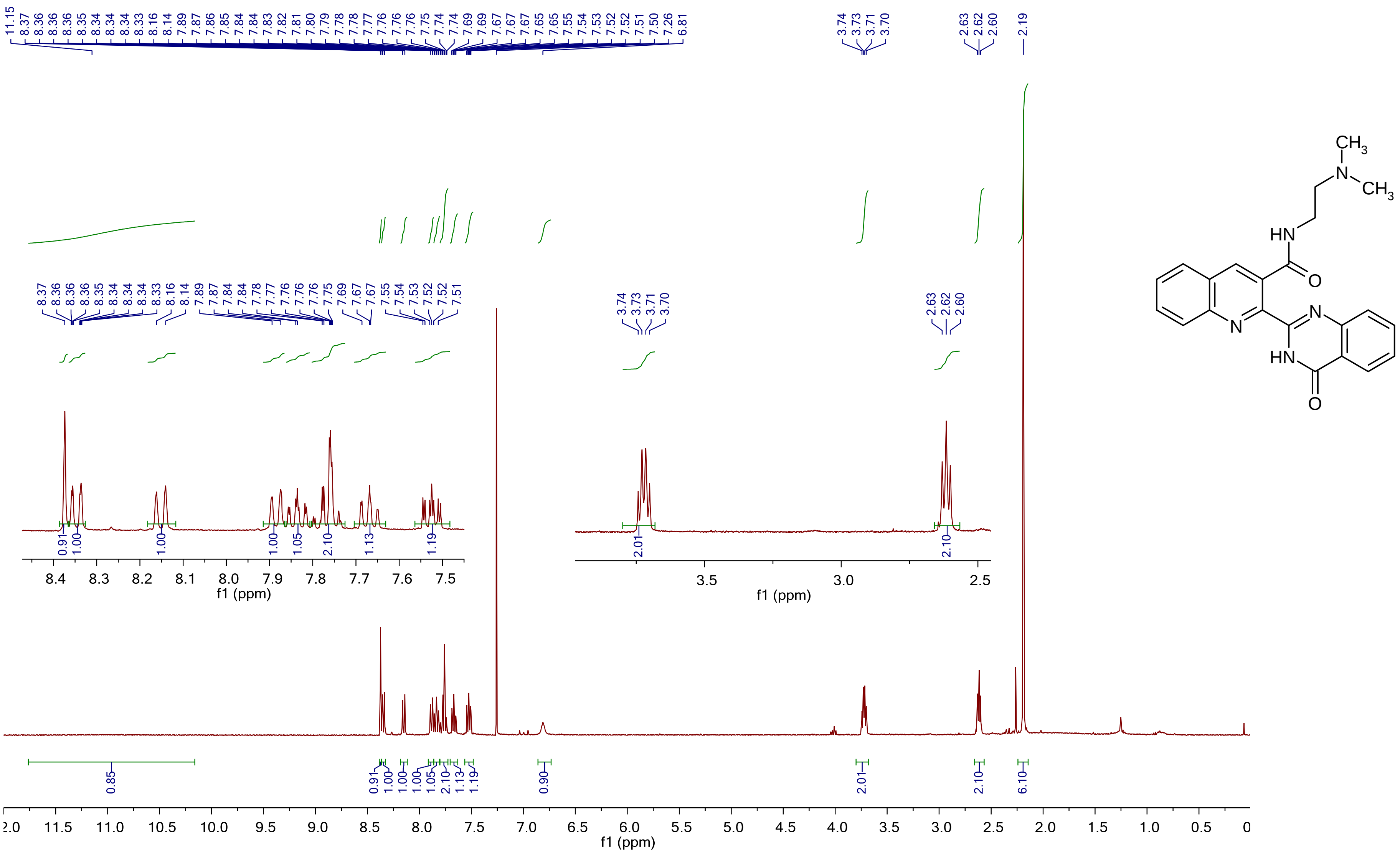

Figure S61. ^{1}H-NMR spectrum of *N*-[2-(dimethylamino)ethyl]-2-(4-oxo-3,4-dihydroquinazolin-2-yl)quinoline-3-carboxamide (**12**)

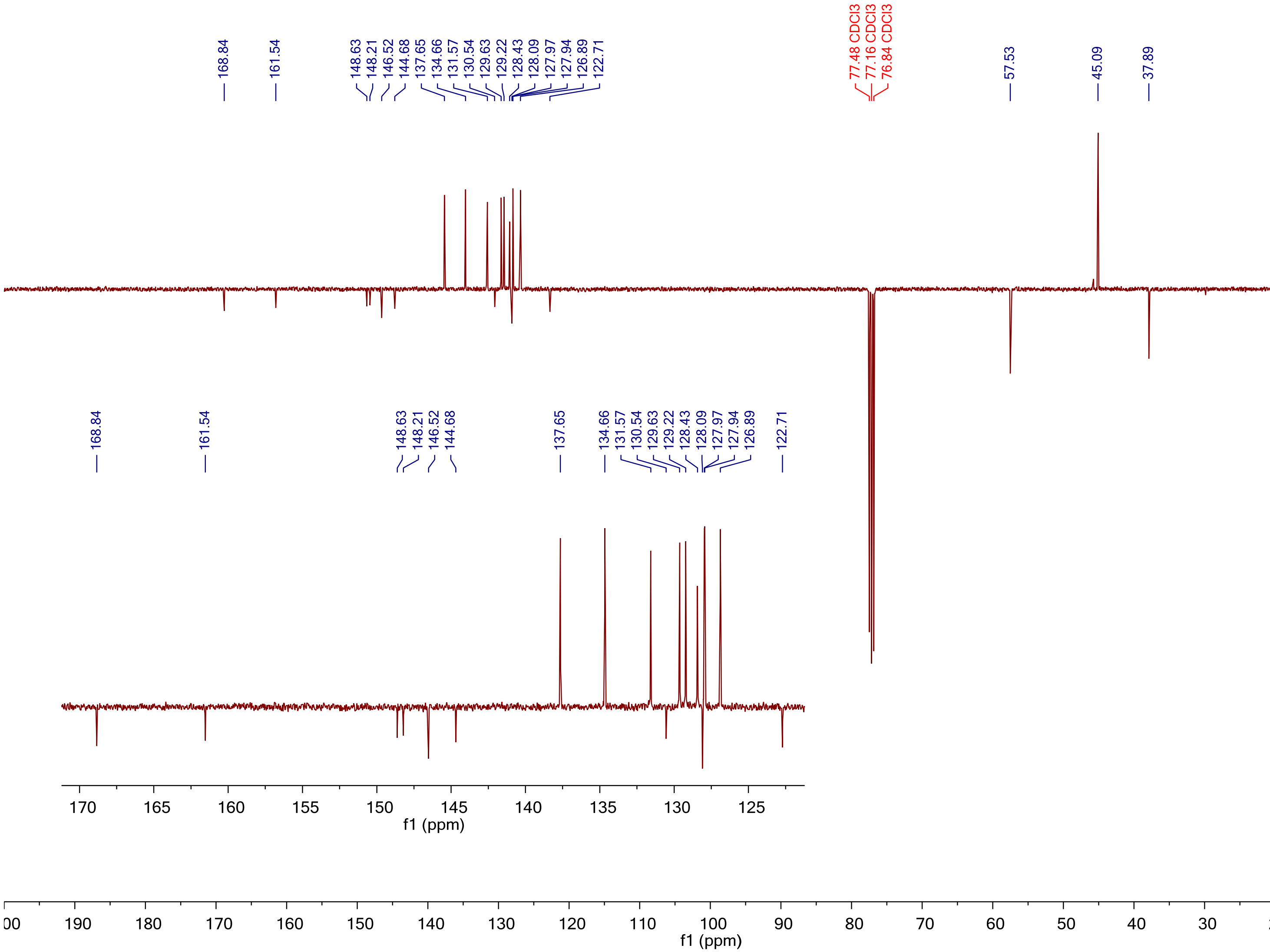

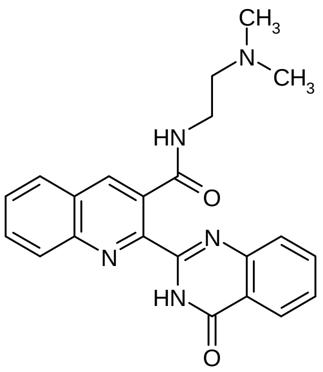

Figure S62. ^{13}C-NMR spectrum of *N*-[2-(dimethylamino)ethyl]-2-(4-oxo-3,4-dihydroquinazolin-2-yl)quinoline-3-carboxamide (**12**)

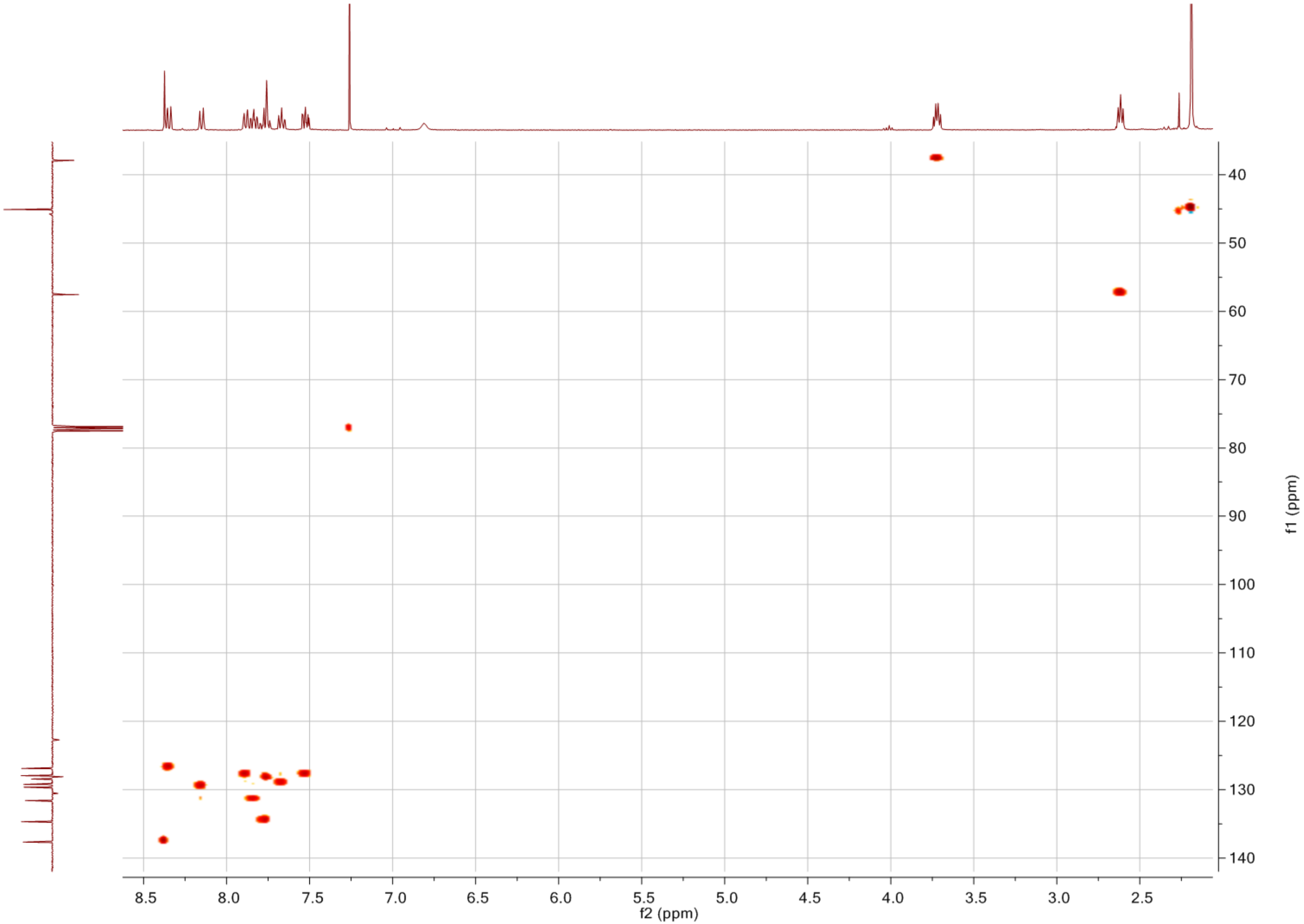

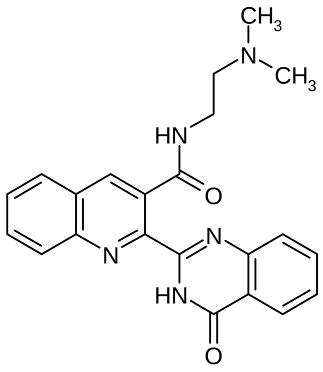

Figure S63. HSQC spectrum of *N*-[2-(dimethylamino)ethyl]-2-(4-oxo-3,4-dihydroquinazolin-2-yl)quinoline-3-carboxamide (**12**)

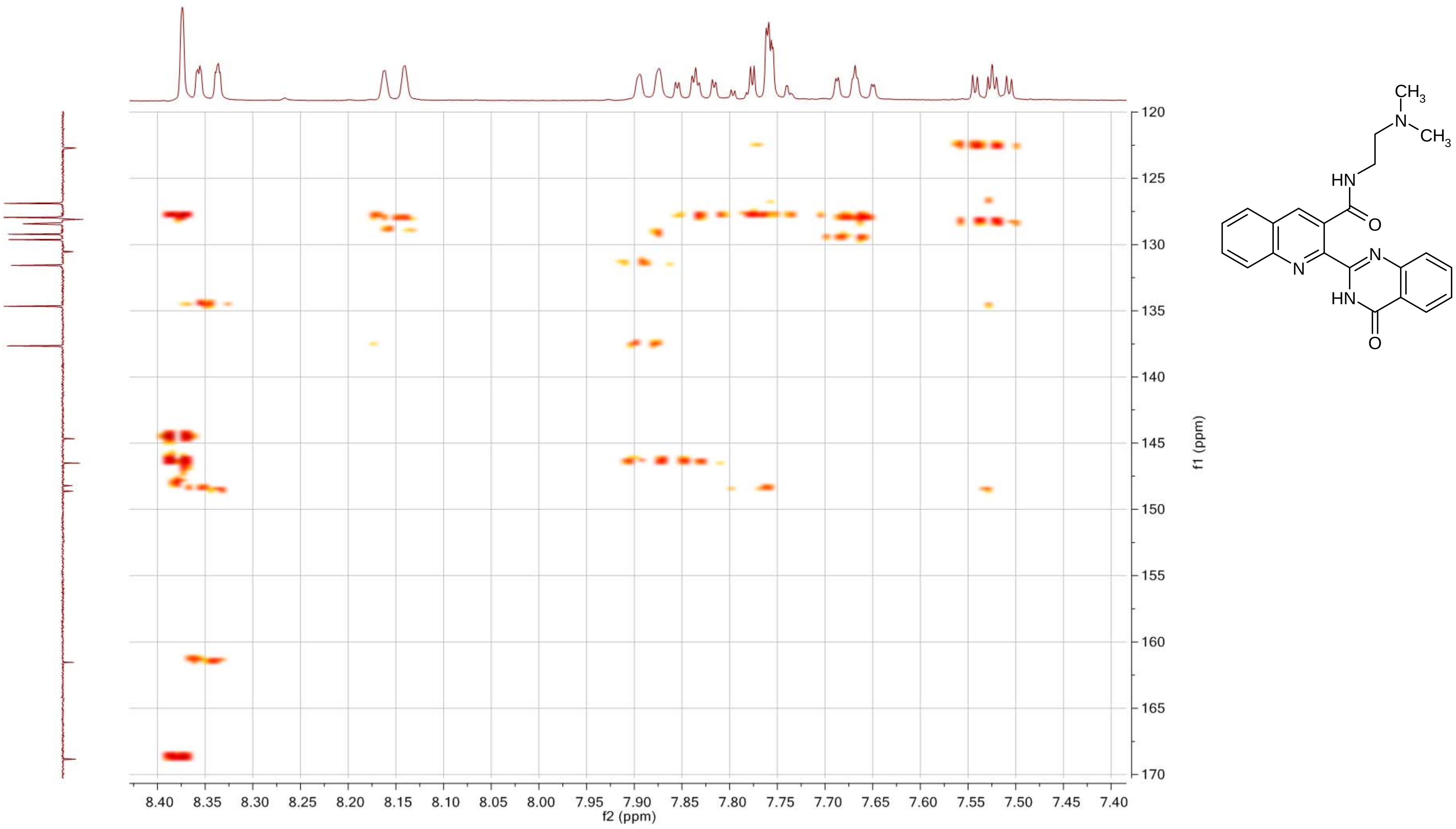

Figure S64. HMBC spectrum of *N*-[2-(dimethylamino)ethyl]-2-(4-oxo-3,4-dihydroquinazolin-2-yl)quinoline-3-carboxamide (**12**)

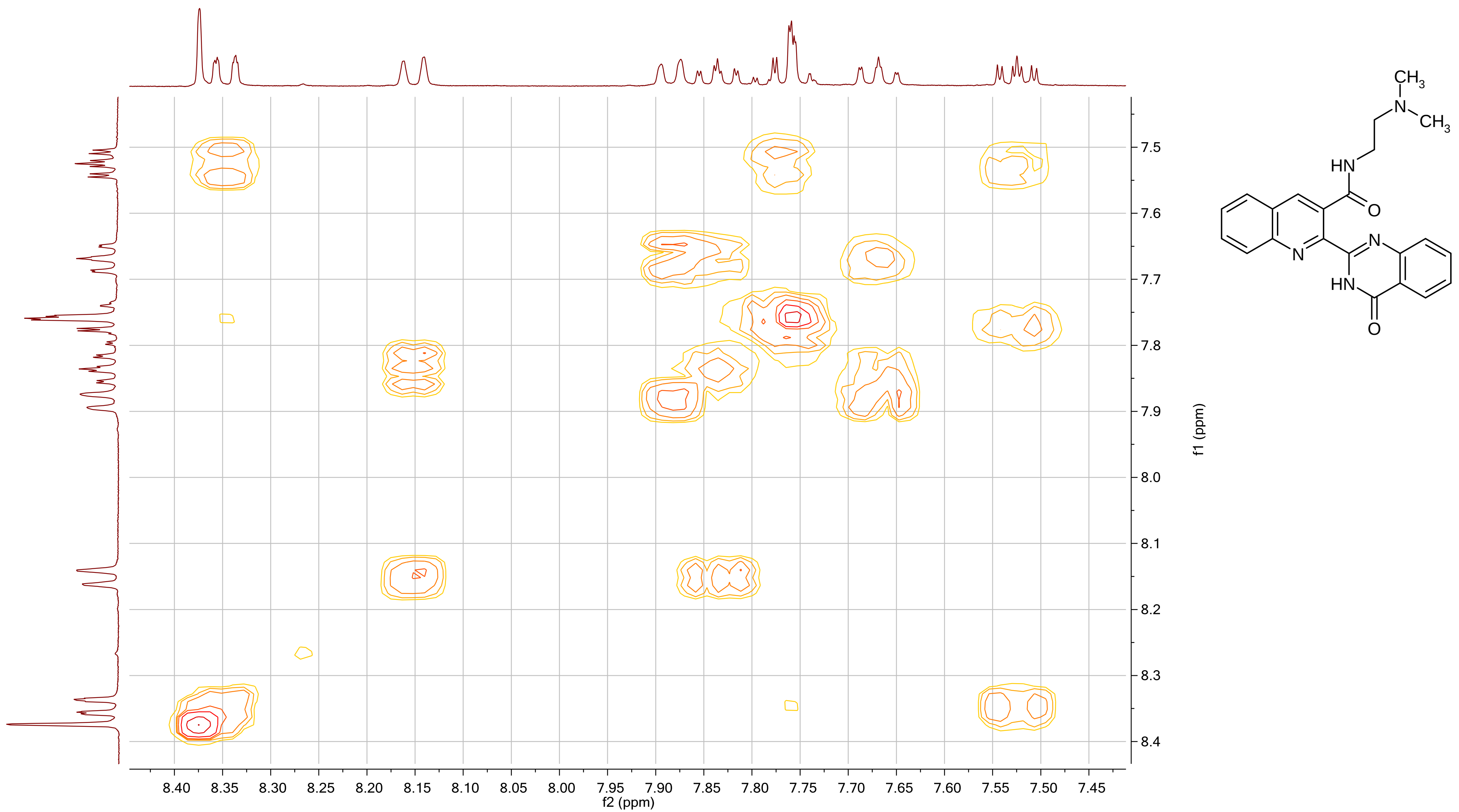

Figure S65. COSY spectrum of *N*-[2-(dimethylamino)ethyl]-2-(4-oxo-3,4-dihydroquinazolin-2-yl)quinoline-3-carboxamide (**12**)

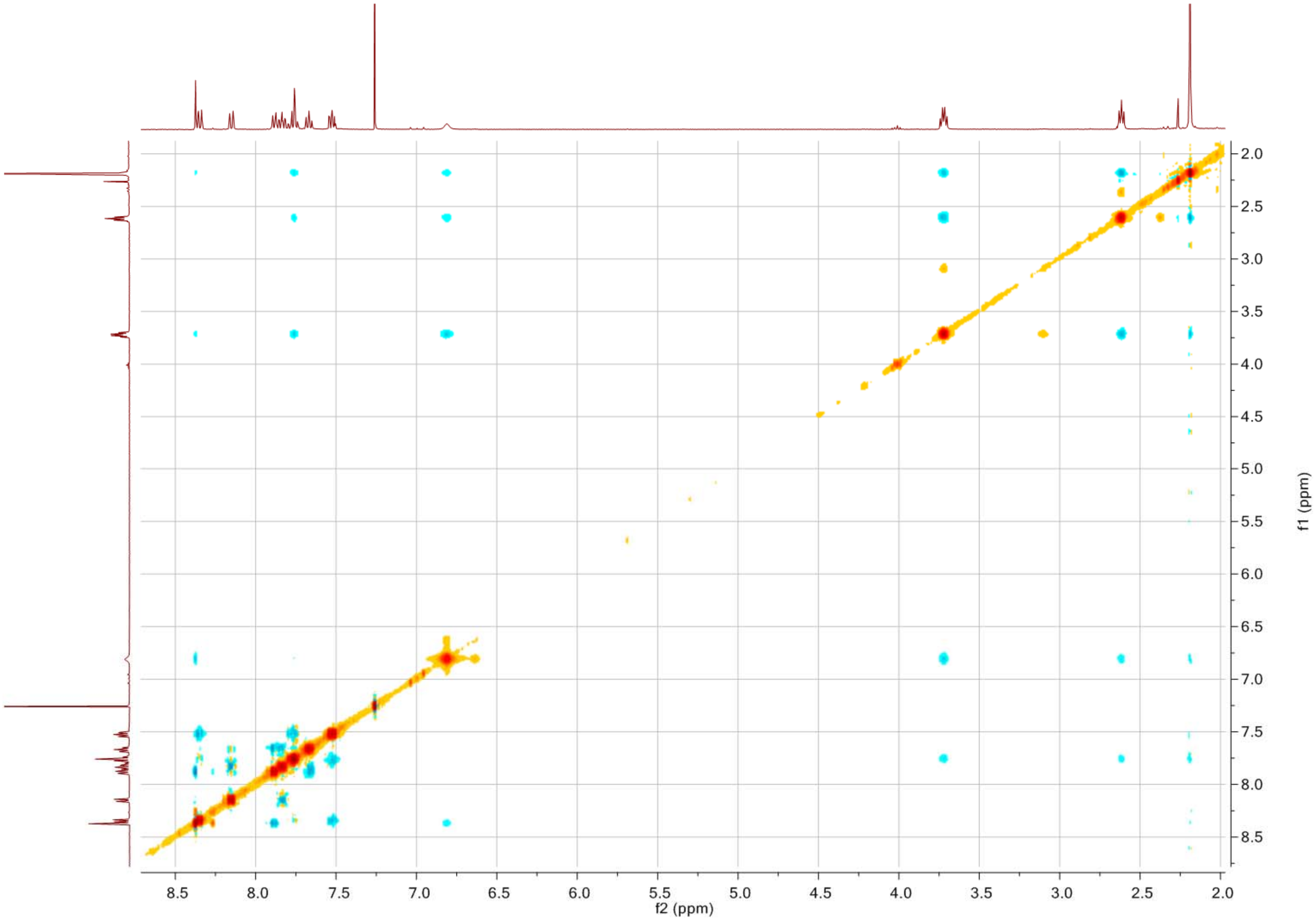

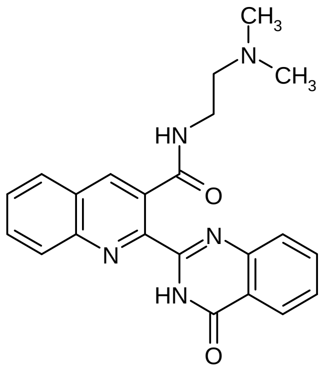

Figure S66. NOESY spectrum of *N*-[2-(dimethylamino)ethyl]-2-(4-oxo-3,4-dihydroquinazolin-2-yl)quinoline-3-carboxamide (**12**)

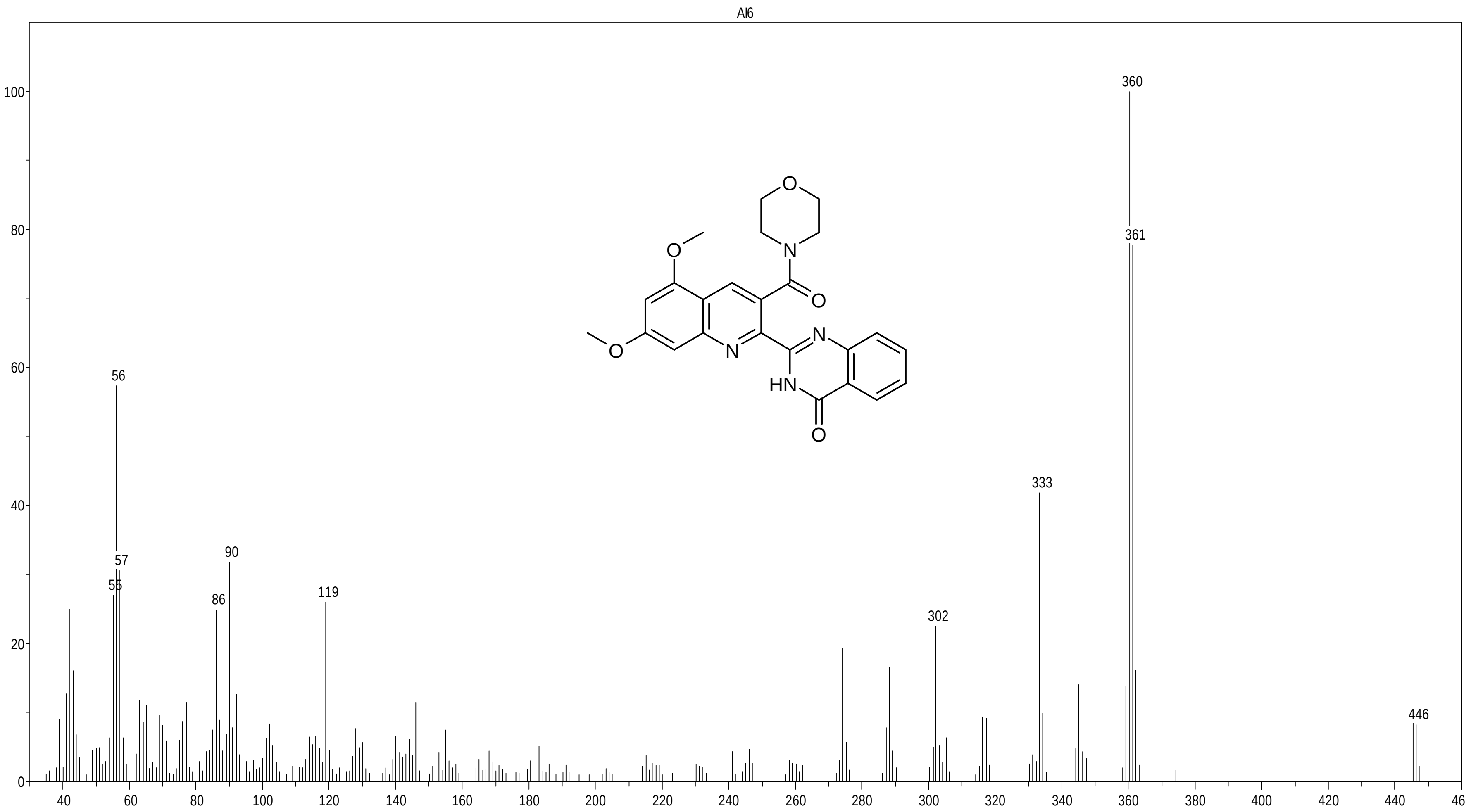

Figure S67. EI-MS of 2-[5,7-dimethoxy-3-(morpholin-4-ylcarbonyl)quinolin-2-yl]quinazolin-4(3*H*)-one (**14**)

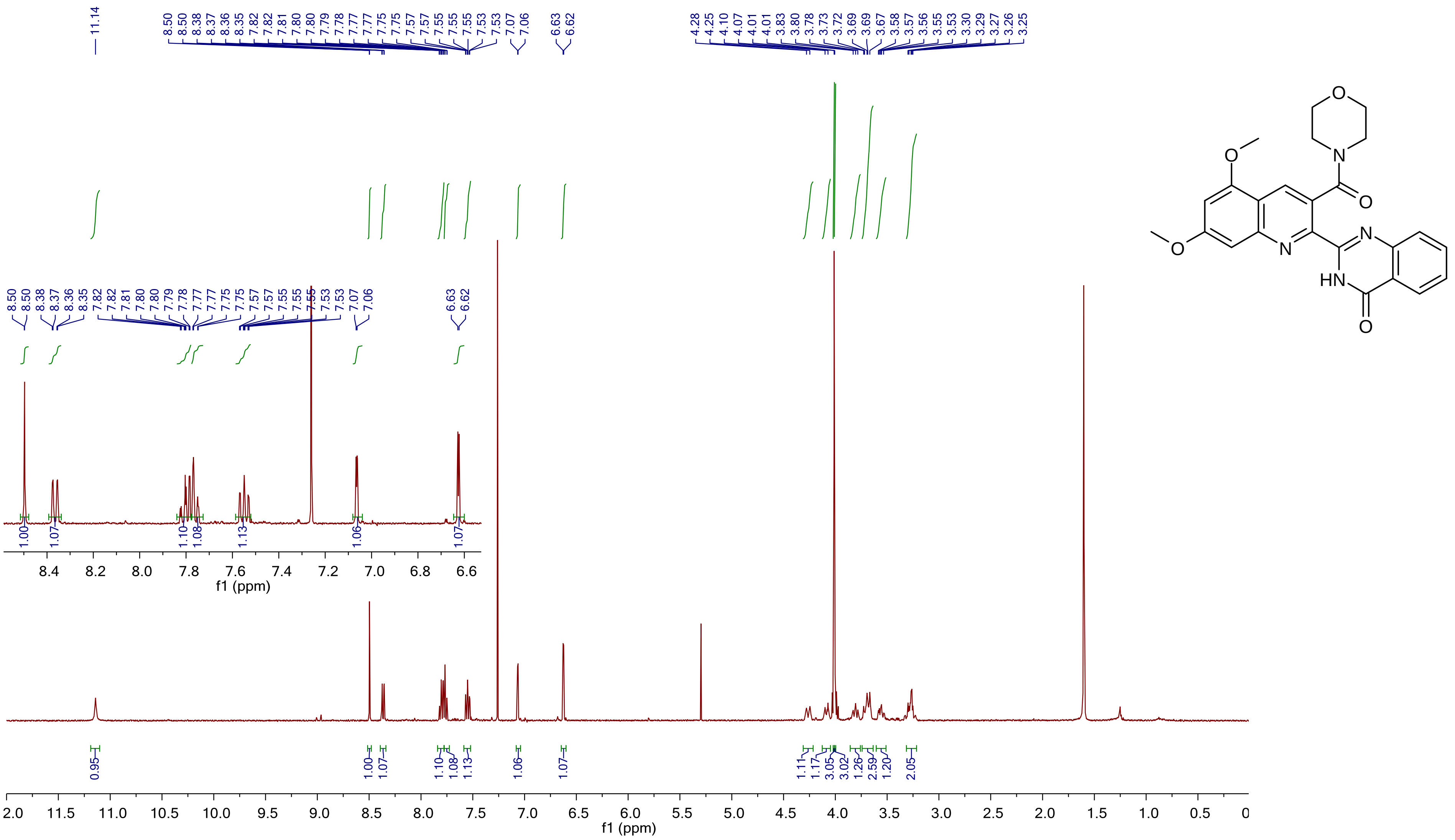

Figure S68. ^{1}H-NMR spectrum of 2-[5,7-dimethoxy-3-(morpholin-4-ylcarbonyl)quinolin-2-yl]quinazolin-4(3*H*)-one (**14**)

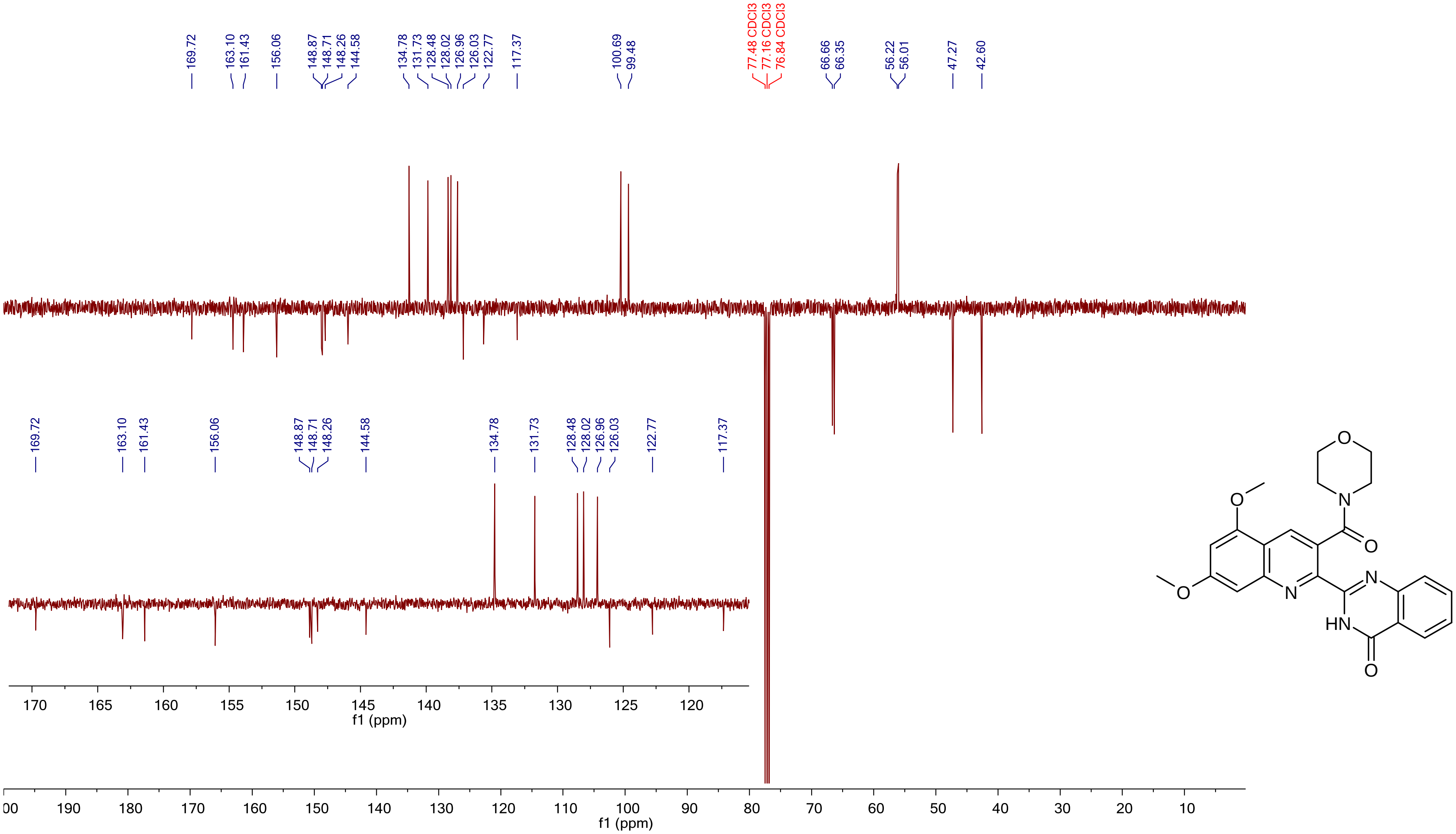

Figure S69. ^{13}C-NMR spectrum of 2-[5,7-dimethoxy-3-(morpholin-4-ylcarbonyl)quinolin-2-yl]quinazolin-4(3*H*)-one (**14**)

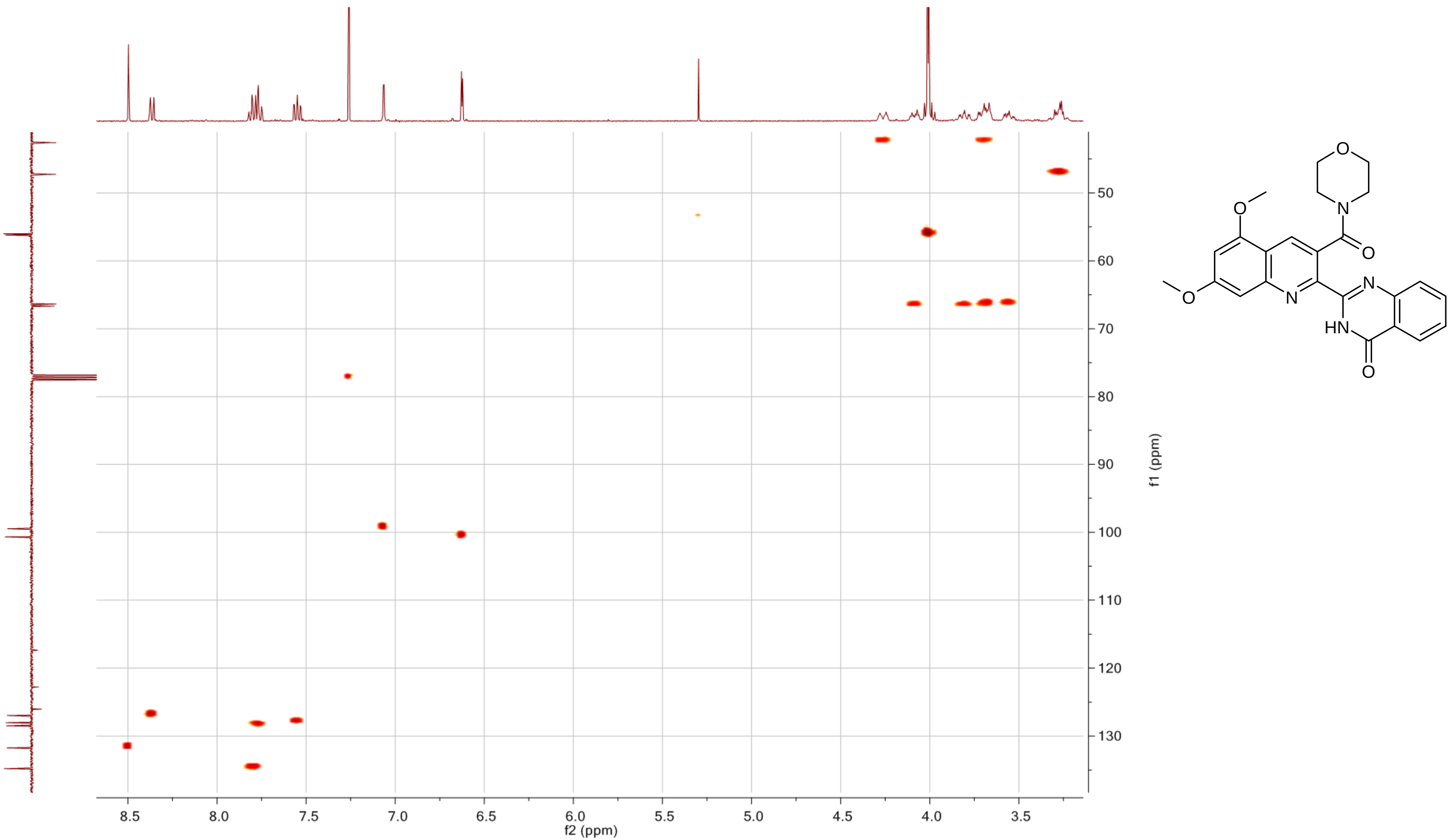

Figure S70. HSQC spectrum of 2-[5,7-dimethoxy-3-(morpholin-4-ylcarbonyl)quinolin-2-yl]quinazolin-4(3*H*)-one (**14**)

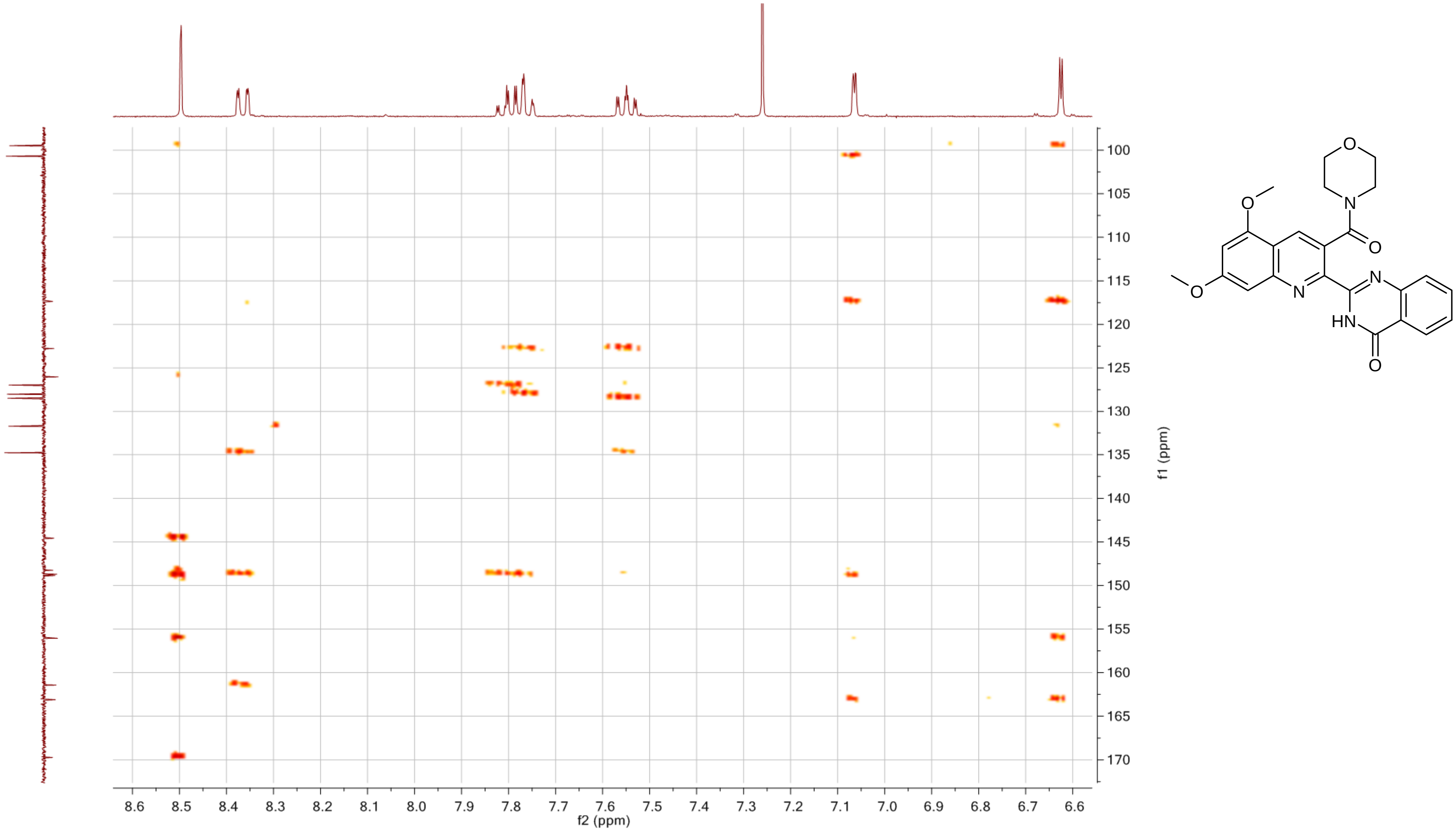

Figure S71. HMBC spectrum of 2-[5,7-dimethoxy-3-(morpholin-4-ylcarbonyl)quinolin-2-yl]quinazolin-4(3*H*)-one (**14**)

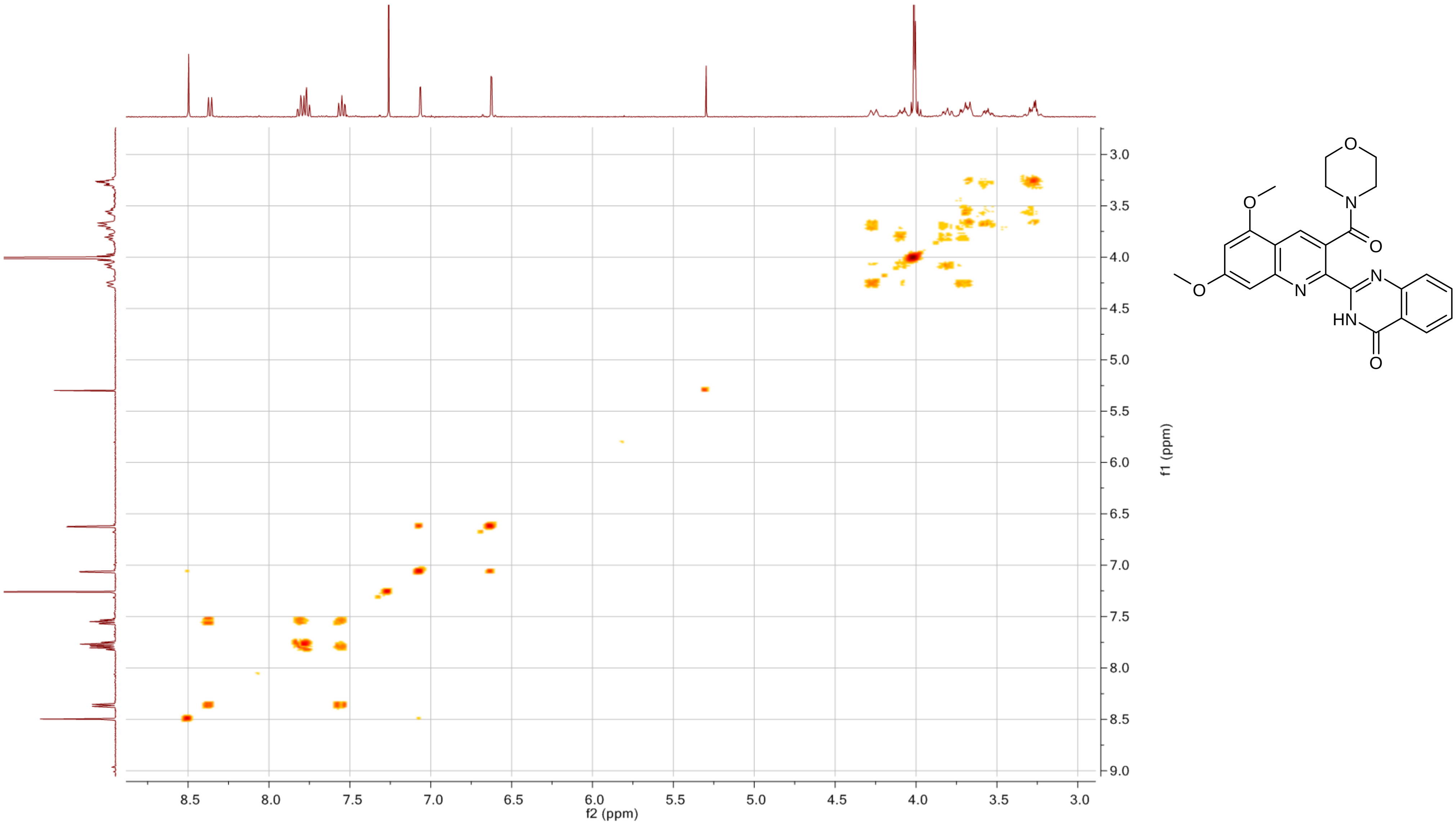

Figure S72. COSY spectrum of 2-[5,7-dimethoxy-3-(morpholin-4-ylcarbonyl)quinolin-2-yl]quinazolin-4(3*H*)-one (**14**)

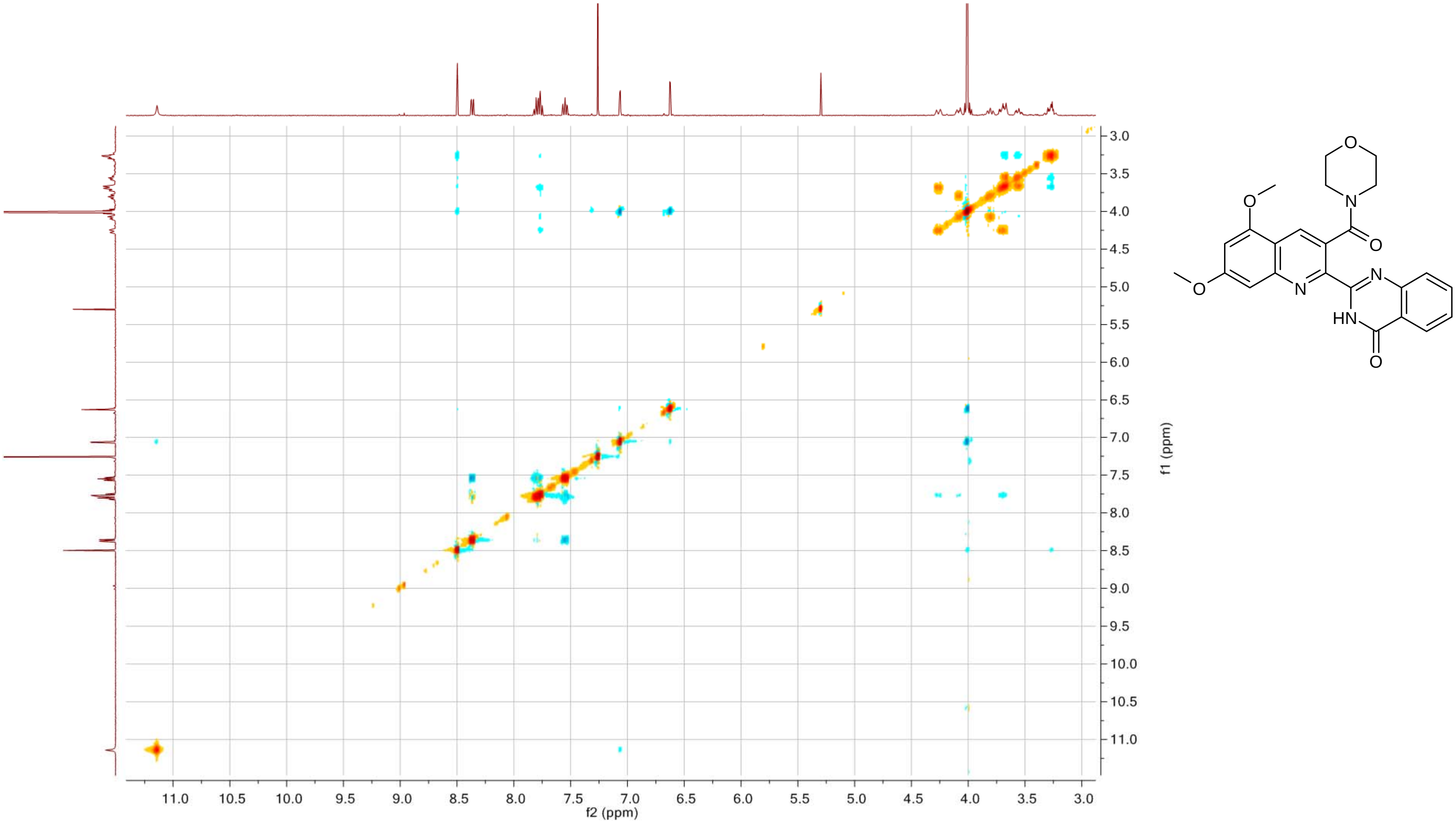

Figure S73. NOESY spectrum of 2-[5,7-dimethoxy-3-(morpholin-4-ylcarbonyl)quinolin-2-yl]quinazolin-4(3*H*)-one (**14**)
